# Computer-Assisted Proofs of Hopf Bubbles and Degenerate Hopf Bifurcations

**DOI:** 10.1007/s10884-023-10279-x

**Published:** 2023-07-04

**Authors:** Kevin Church, Elena Queirolo

**Affiliations:** 1 Université de Montréal, Montreal, Canada; 2 Technische Universität München, Munich, Germany

## Abstract

We present a computer-assisted approach to prove the existence of Hopf bubbles and degenerate Hopf bifurcations in ordinary and delay differential equations. We apply the method to rigorously investigate these nonlocal orbit structures in the FitzHugh–Nagumo equation, the extended Lorenz-84 model and a time-delay SI model.

## Introduction

The Hopf bifurcation is a classical mechanism leading to the birth of a periodic orbit in a dynamical system. In the simplest setting of an ordinary differential equation (ODE), a Hopf bifurcation requires the linearization at a fixed point to have a single pair of complex-conjugate imaginary eigenvalues. In this case, a perturbation by way of a scalar parameter would be expected, but not guaranteed, to result in a Hopf bifurcation. To prove the existence of the Hopf bifurcation, some non-degeneracy conditions must be checked. We refer the reader to the papers [6, 9, 14, 22, 28] for some classical (and more recent) background concerning the Hopf bifurcation in the context of infinite-dimensional dynamical systems. A standard reference for the ODE case is the book of Marsden & McCracken [24].

If the non-degeneracy conditions of a Hopf bifurcation are not satisfied, the bifurcation might not occur at all, or there could be other structures present that are not fully described by the usual bifurcation diagram. We refer to such cases as *degenerate Hopf bifurcations*. One way to capture these other structures is to consider the effect of varying more than a single parameter. For most mathematical models, such an exercise is perfectly natural, since few models depend on only one parameter. Moreover, distinguishing regions in parameter space that support periodic orbits is important for a thorough qualitative understanding of a model. In the following two sub-sections, we will describe two degenerate Hopf bifurcations, although there are others; see later Sect. 1.3.

### Hopf Bubbles

In a Hopf bifurcation, a single pair of complex-conjugate eigenvalues must cross the imaginary axis, as the parameter varies, in a transversal way. If the eigenvalues do not cross, the crossing is tangential, or there are other eigenvalues with zero real part, this can lead to a degenerate Hopf bifurcaiton. One particular case is where the curve of eigenvalues has a quadratic tangency with the imaginary axis. Before surveying some literature about this bifurcation pattern, let us construct a minimal working example. Consider the ODE system

1 $$\begin{aligned} \dot{x}&=\beta x - y -x(x^2+y^2+\alpha ^2) \end{aligned}$$

2 $$\begin{aligned} \dot{y}&=x + \beta y -y(x^2+y^2+\alpha ^2). \end{aligned}$$

The reader familiar with the normal form of the Hopf bifurcation should find this familiar, but might be be unnerved by the $\alpha ^2$ term, which is not present in the typical normal form. The linearization at the equilibrium (0, 0) produces the matrix

$$\begin{aligned} \left( \begin{array}{cc} \beta -\alpha ^2 &{} -1 \\ 1 &{} \beta -\alpha ^2 \end{array}\right) , \end{aligned}$$

which has the pair of complex-conjugate eigenvalues $\lambda = \beta - \alpha ^2 \pm i$. Treating $\alpha$ as being a fixed constant, we have a supercritical Hopf bifurcation as $\beta$ passes through $\alpha ^2$. Conversely, if $\beta >0$ is fixed and we interpret $\alpha$ as the parameter, there are *two* supercritical Hopf bifurcations: as $\alpha$ passes through $\pm \sqrt{\beta }$. However, something problematic happens if $\beta =0$: the eigenvalue branches $\alpha \mapsto -\alpha ^2 \pm i$ are tangent to the imaginary axis at $\alpha =0$ and do not cross at all, so the non-degeneracy condition of the Hopf bifurcation fails.

More information can be gleaned by transforming to polar coordinates. Setting $x=r\cos \theta$ and $y=r\sin \theta$ results in the equation for the radial component

$$\begin{aligned} \dot{r} = r(\beta - r^2 - \alpha ^2), \end{aligned}$$

while $\dot{\theta }=1$. There is a nontrivial periodic orbit (in fact, limit cycle) if $\beta -\alpha ^2>0$, with amplitude $\sqrt{\beta -\alpha ^2}$. Alternatively, there is a *surface of periodic orbits* described by the graph of $\beta = r^2+\alpha ^2$. For fixed $\beta >0$, the amplitude *r* of the periodic orbit, as a function of $\alpha$, is $\alpha \mapsto \sqrt{\beta -\alpha ^2}$, whose graph is half of an ellipse with semi-major axis $2\sqrt{\beta }$ and semi-minor axis $\sqrt{\beta }$.

This non-local orbit structure, characterized by the connection of two Hopf bifurcations by a one-parameter branch of periodic orbits, has been given several names in different scientific fields. In intracellular calcium, it is frequently called a *Hopf bubble* [7, 17, 25, 30]. In infectious-disease modelling, where the bifurcation typically occurs at an endemic equilibrium, the accepted term is *endemic bubble* [8, 18, 21, 26, 29]. A more general definition of a (parametrically) non-local structure called *bubbling* is given in [15]. In the present work, we will refer to the structure as a *Hopf bubble*, since that name is descriptive of the geometric picture, the bifurcation involved, and is sufficiently general to apply in different scenarios in a model-independent way.

Hopf bubbles can, in many instances, be understood as being generated by a codimension-two bifurcation; see Sect. 1.3. However, this is not to say that they are rare. Aside from the applications in calcium dynamics and infectious-disease modelling in the previous paragraph, they have been observed numerically in models of neurons [1], condensed-phase combustion [23], predator–prey models [3], enzyme-catalyzed reactions [13], and a plant-water ecosystem model [36]. A recent computer-assisted proof also established the existence of a Hopf bubble in the Lorenz-84 model [33]. We will later refer to the degenerate Hopf bifurcation that gives rise to Hopf bubbles as a *bubble bifurcation*. The literature seems to not have an accepted name for this bifurcation, so we have elected to give it one here.

### Bautin Bifurcation

Bubble bifurcations are one type of degenerate Hopf bifurcation. Another is the Bautin bifurcation, which occurs at a Hopf bifurcation whose first Lyapunov coefficient vanishes [16]. Like the bubble bifurcation, the Bautin bifurcation is a codimension-two bifurcation of periodic orbits. To illustrate this bifurcation, consider the planar normal form

3 $$\begin{aligned} \dot{x}&=\beta x - y -x(x^2+y^2)(\alpha - x^2-y^2) \end{aligned}$$

4 $$\begin{aligned} \dot{y}&=x + \beta y -y(x^2+y^2)(\alpha - x^2-y^2). \end{aligned}$$

Transforming to polar coordinates, the angular component decouples producing $\dot{\theta }=1$, while the radial component gives

$$\begin{aligned} \dot{r} = r(\beta + \alpha r^2 - r^4). \end{aligned}$$

Nontrivial periodic orbits are therefore determined by the zero set of $r\mapsto \beta +\alpha r^2 - r^4$, which defines a two-dimensional smooth manifold. See later Fig. 15 for a triangulation of (part of) this manifold.

### Degenerate Hopf Bifurcations and Multi-Parameter Continuation

The Hopf bubble and the Bautin bifurcation are far from the only degenerate Hopf bifurcations. Indeed, the class includes also the Bogdanov–Takens bifurcation, singular Hopf bifurcations [12], the Hamiltonian-Hopf bifurcation [35] and Hopf bifurcations without parameters [20], among others. The bubble bifurcation has been fully characterized by LeBlanc [18], using the center manifold reduction and normal form theory for functional differential equations [9] and a prior classification of degenerate Hopf bifurcations for ODEs by Golubitsky and Langford [11]. The study of the Bautin bifurcation goes back to 1949 with the work of Bautin [2], and a more modern derivation based on normal form theory appears in the textbook of Kuznetsov [16].

Normal form theory and centre manifold reduction are incredibly powerful, providing both the direction of the bifurcation and criticality of bifurcating periodic orbits. The drawback is that they are inherently local: it is difficult to obtain information about the persistence of limit cycles as a function of the parameter, away from the bifurcation point. In the present work, we advocate for an analysis of degenerate Hopf bifurcations in ODE and delay differential equations (DDE) by way of *two-parameter continuation*, *desingularization* and *computer-assisted proofs*. We will discuss the latter topic in the next section.

**Fig. 1:**
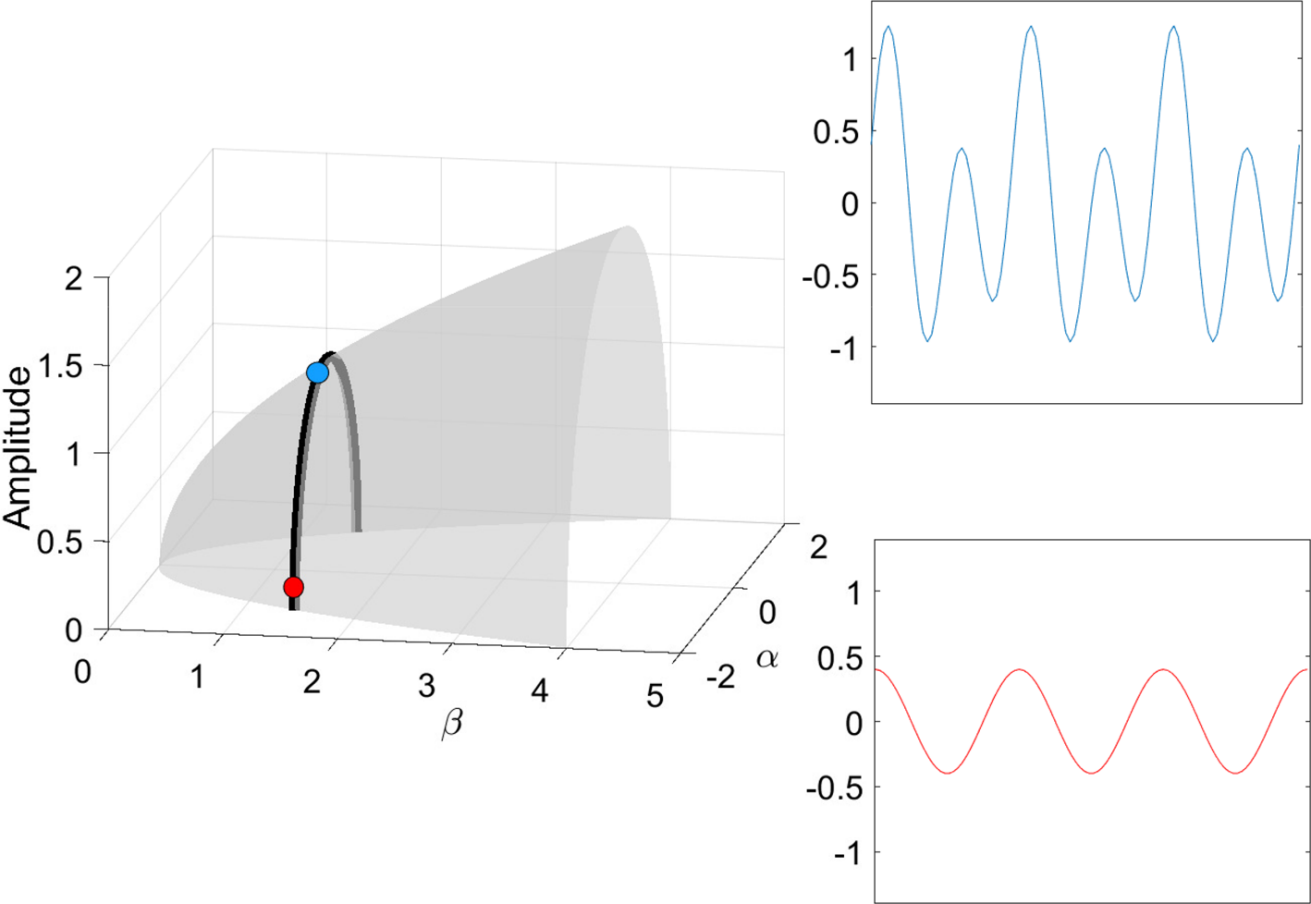
Near a Hopf bifurcation, the bifurcating periodic orbit is close to a pure cosine (red solution) with small amplitude. Far from the bifurcation, the amplitude tends to grow and more Fourier modes are represented (blue solution). In a Hopf bubble, the amplitude grows from zero and then decreases to zero as a parameter ($\alpha$ in this figure) is varied montonically. Variation of a control parameter ($\beta$) can result in a smaller range of amplitudes and admissible interval over which the periodic orbit persists. If variation of the control parameter ultimately results in a collapse of the amplitude to zero, while the admissible interval (for the periodic orbit) converges to a point, the result is a bubble bifurcation (Color figure online)

The intuition behind our continuation idea can be pictorially seen in Fig. 1, and understood analytically by way of our minimal working example, (1)–(2), and its surface of periodic orbits described by the equation $\beta = \alpha ^2+r^2$. There is an implicit relationship between two scalar parameters ($\alpha$ and $\beta$) and the set of periodic orbits of the dynamical system. At a Hopf bifurcation, one of these periodic orbits should retract onto a steady state. In other words, their amplitudes (relative to steady state) should become zero. Using a desingularization technique to topologically separate periodic orbits from steady states that they bifurcate from, we can use two-parameter continuation to numerically compute and continue periodic orbits as they pass through a degenerate Hopf bifurcation. With computer-assisted proof techniques, we can prove that the numerically-computed objects are close to true periodic orbits, with explicit error bounds. Further a posteriori analysis can then be used to prove the existence of a degenerate Hopf bifurcation based on the output of the computer-assisted proof of the continuation.

### Rigorous Numerics

Computer-assisted proofs of Hopf bifurcations have been completed in [33] using a desingularization (sometimes called blow-up) approach, in conjunction with an *a posteriori* Newton–Kantorvich-like theorem. We lean heavily on these ideas in the present work. The former desingularization idea, which we adapt to delay equations in Sect. 3.1, is used to resolve the fact that, at the level of a Fourier series, a periodic orbit that limits (as a parameter varies) to a fixed point at a Hopf bifurcation is, itself, indistinguishable from that fixed point. Our methods of computer-assisted proof are based on contraction mappings, and it is critical that the objects we prove are isolated. The desingularization idea exploits the fact that an amplitude-based change of variables can be used to develop an equivalent problem where the representative of a periodic orbit consists of a Fourier series, a fixed point and a real number. The latter real number represents a signed amplitude. This reformulation results in fixed points being spatially islolated from periodic orbits, thereby allowing contraction-based computer-assisted proofs to succeed.

The other point of inspiration in this work is *validated multi-parameter continuation*. The technique was developed in [10] for continuation in general Banach spaces, and applied to some steady state problems for PDEs. We will overview this method in Sect. 2.

### Contributions and Applications

The main contributions of the present work are

1. A general-purpose code for computing and proving two-parameter families of periodic orbits in polynomial delay differential equations;
2. Theoretical foundations behind this code, and approaches to proving Hopf bifurcation curves, bubble bifurcations, and a more broad class of degenerate Hopf bifurcations based on outputs of the computer-assisted proof;
3. Applications of this code to proving two-parameter families of periodic orbits, both far from and at degenerate Hopf bifurcations.

Equations of advanced or mixed-type delay can similarly be handled; there is no restriction whether delays are positive or negative. Ordinary differential equations can also be handled as a special case. Orbits can be proven in the vicinity of (and at) Hopf bifurcations, whether these are non-degenerate or degenerate. The first major release of the library BiValVe (**Bi**furcation **Val**idation **Ve**nture, [5]) is being made in conjunction with the present work, and builds on an earlier version of the code associated to the work [33, 34]. Some non-polynomial delay differential equations can be handled using the *polynomial embedding technique*. The existence of Hopf bifurcation curves and degenerate Hopf bifurcations can then be completed by post-processing of the output of the computer-assisted proof.

To explore the applicability of our validated numerical methods, we explore Hopf bifurcations and degenerate Hopf bifurcations in

- The extended Lorenz-84 model (ODE)
- A time-delay SI model (DDE)
- The FitzHugh–Nagumo equation (ODE)
- An ODE with complicated branches of periodic orbits (ODE)

The first two examples replicate and extend some of the analysis appearing in [18, 33] using our computational scheme. The third examples provides, to our knowledge, the first analytically verified results on degenerate Hopf bifurcations and Hopf bubbles in that equation. The final example is a carefully designed ODE that exhibits the degenerate Hopf bifurcation in addition to folding and pinching in some projections of the periodic orbit 2-manifold.

### Overview of the Paper

Section 2 serves as an overview of two-parameter continuation, both in the finite-dimensional and infinite-dimensional case. We introduce the continuation scheme for periodic orbits near Hopf bifurcations in Sect. 3 in the general case of delay differential equations. Technical bounds for the computer-assisted proofs are derived in Sect. 4. A specification to ordinary differential equations is presented in Sect. 5. In Sect. 6, we connect the computer-assisted proofs of the manifold of periodic orbits to analytical proofs of Hopf bifurcation curves, Hopf bubbles, and degenerate Hopf bifurcations. Our examples are presented in Sect. 7, and we complete a discussion and comment on future research directions in Sect. 8.

### Notation and Important Notes

In this section, we introduce some notation and conventions that will be used throughout the paper. The reader is encouraged to consult this section should they encounter an unfamiliar symbol, as there is a strong likelihood it is defined herein.

Given $n\in {\mathbb {N}}$, denote ${\mathbb {C}}^{\mathbb {Z}}_n$ the vector space of ${\mathbb {Z}}$-indexed sequences of elements of ${\mathbb {C}}^n.$ Denote $\text{ Symm }({\mathbb {C}}^{\mathbb {Z}}_n)$ the proper subspace of ${\mathbb {C}}^{\mathbb {Z}}_n$ consisting of symmetric sequences; $z\in \text{ Symm }({\mathbb {C}}^{\mathbb {Z}}_n)$ if and only if $z\in {\mathbb {C}}^{\mathbb {Z}}_n$ and $z_k=\overline{z_{-k}}$ for all $k\in {\mathbb {Z}}$. For any subspace $U\subset {\mathbb {C}}^{\mathbb {Z}}_n$ closed under (componentwise) complex conjugation, denote ${{\,\textrm{Symm}\,}}(U) = U\cap {{\,\textrm{Symm}\,}}({\mathbb {C}}^{\mathbb {Z}}_n)$. We will sometimes drop the subscript *n* on ${\mathbb {C}}^{\mathbb {Z}}_n$ when the context is clear.

Given $\nu >1$, we denote $\ell _\nu ^1({\mathbb {C}}^n)$ the subspace of ${\mathbb {C}}_n^{\mathbb {Z}}$ whose elements *z* satisfy $||z||_\nu \overset{\textrm{def}}{=}\sum _{k\in {\mathbb {Z}}}|z_k|\nu ^{|k|}<\infty$. The symbol $|\cdot |$ will always be taken to be the norm on ${\mathbb {C}}^n$ induced by the standard inner product. Denote $K_\nu ({\mathbb {C}}^n)$ the subspace of ${\mathbb {C}}_n^{\mathbb {Z}}$ whose elements *z* satisfy $||z||_{\nu ,K}\overset{\textrm{def}}{=}|z_0| + \sum _{|k|>0}(\nu ^{|k|}/|k|)|z_k|<\infty .$ Introduce a bilinear form on $\langle \cdot ,\cdot \rangle$ on $\ell _\nu ^1({\mathbb {C}})$ as follows:

$$\begin{aligned} \langle v,w\rangle = \sum _{k\in {\mathbb {Z}}}v_k\overline{w_k}. \end{aligned}$$

For $v,w\in {\mathbb {C}}^{\mathbb {Z}}_1$, define their convolution $v*w$ by

5 $$\begin{aligned} (v*w)_k = \sum _{k_1+k_2=k}v_{k_1}w_{k_2} \end{aligned}$$

whenever this series converges. Convolution is commutative and associative for sequences in $$\ell _\nu ^1({\mathbb {C}})$$. In this case, we define multiple convolutions (e.g. triple convolutions $$a*b*c$$) inductively, by associativity.

A function $g:\ell _\nu ^1({\mathbb {C}}^n)\rightarrow \ell _\nu ^1({\mathbb {C}})$ is a *convlution polynomial of degree* *q* if

$$\begin{aligned} g(z)=c_0 + \sum _{k=1}^q\sum _{p\in {\mathcal {M}}_k} c_p (z_{p_1}*\cdots *z_{p_k}), \end{aligned}$$

for $c_p\in {\mathbb {C}}$, where ${\mathcal {M}}_k$ denotes the set of *k*-element multisets of $\{1,\dots ,n\}$, and each multiset *p* is identified with the unique tuple $(p_1,\dots ,p_k)\{1,\dots ,n\}^k$ such that $p_i\le p_{i+1}$ for all $i=1,\dots ,k-1$. Analogously, $g:\ell _\nu ^1({\mathbb {C}}^n)\rightarrow \ell _\nu ^1({\mathbb {C}}^m)$ is a *convolution polynomial of degree* *q* if $z\mapsto g(z)_{\cdot ,j}\in \ell _\nu ^1({\mathbb {C}})$ is a convolution polynomial of degree *q*, for $j=1\dots ,m$.

If *X* is a vector space, $0_X$ will denote the zero vector in that space. If *X* is a metric space and $U\subset X$, we denote $U^\circ$ its interior, $\partial U$ its boundary, and $\overline{U}$ its closure.

In this work, any reference to a norm on ${\mathbb {R}}^k$ or ${\mathbb {C}}^k$ for some $k\ge 1$ should always be understood to be a *weighted supremum norm* with respect to the standard ordered basis. That is, in all cases, there will exist $w_1,\dots ,w_k>0$ such that $||(x_1,\dots ,x_k)|| =\max \{w_1|x_1|,\dots ,w_k|x_k|\}$. These weights will either be specified, or it will be clear from context that they are to be selected as part of a computer-assisted proof.

An *interval vector* in ${\mathbb {R}}^k$ for some $k\ge 1$ is a subset of the form $v = [a_1,b_1]\times \cdots \times [a_k,b_k]$ for real scalars $a_j,b_j\in {\mathbb {R}}$, $j=1,\dots ,k$. We define $||v||=\sup _{w\in v}||w||$ (see the previous paragraph; the norm on the right side is a weighted supremum norm). Similarly, an interval vector in ${\mathbb {C}}^k$ is a product $v = A_1\times \cdots \times A_k$, where each $A_j$ is a closed disc in ${\mathbb {C}}$. We define the norm of a complex interval vector as $||v|| = \sup _{w\in v}||w||$.

## Validated Two-Parameter Continuation

In this section, we review validated two-parameter continuation. Our presentation will loosely follow [10]. Some noteworthy changes compared to the references are that we work in a complexified (as opposed to strictly real) vector space, which causes some minor difficulties at the level of implementation.

We first review the continuation algorithm as it applies to finite-dimensional vector spaces in Sect. 2.1. We make comments concerning implementation in Sect. 2.2. We then describe how it is extended to general Banach spaces in Sect. 2.3. Validated continuation (i.e. computer-assisted proof) is discussed in Sect. 2.4.

### Continuation in a Finite-Dimensional Space

Let ${\mathcal {X}}$ and ${\mathcal {Y}}$ be finite-dimensional vector spaces over the field ${\mathbb {R}}$, with $\dim {\mathcal {X}}= \dim {\mathcal {Y}}+ 2$, and consider a map ${\mathcal {G}}:{\mathcal {X}}\rightarrow {\mathcal {Y}}$. We are interested in the zero set of ${\mathcal {G}}$. Given the codimension of ${\mathcal {G}}$, we expect zeroes to be in a two-dimensional manifold in ${\mathcal {X}}$.

Let $\hat{x}_0\in {\mathcal {X}}$ satisfy ${\mathcal {G}}(\hat{x}_0)\approx 0$, and suppose $D{\mathcal {G}}(\hat{x}_0)$ has two-dimensional kernel. This property is generic. Let $\{\hat{\Phi }_1,\hat{\Phi }_2\}$ span the kernel. The (approximate) tangent plane ${\mathcal {T}}_{\hat{x}_0}{\mathcal {M}}$ at $\hat{x}_0$ of the two-dimensional (approximate) solution manifold ${\mathcal {M}}$ is therefore spanned by $\{\hat{\Phi }_1,\hat{\Phi }_2\}$. If $\epsilon _1,\epsilon _2$ are small, we have

$$\begin{aligned} {\mathcal {G}}(\hat{x}_0 + \epsilon _1\hat{\Phi }_1 + \epsilon _2\hat{\Phi }_2) = {\mathcal {G}}(\hat{x}_0) + \epsilon _1 D{\mathcal {G}}(\hat{x}_0)\hat{\Phi }_1 + \epsilon _2 D{\mathcal {G}}(\hat{x}_0)\hat{\Phi }_2 + o(|\epsilon |) = {\mathcal {G}}(\hat{x}_0) + o(|\epsilon |) \end{aligned}$$

by Taylor’s theorem, provided ${\mathcal {G}}$ is differentiable at $\hat{x}_0$. We would hope that the error of ${\mathcal {G}}(\hat{x}_0)\approx 0$ is smaller than the residual $o(|\epsilon |)$ terms. If ${\mathcal {G}}$ is twice continuously differentiable, the error is improved to $O(|\epsilon |^2)$. Therefore, new candidate zeroes of ${\mathcal {G}}$ can be computed using $\hat{x}_0$ and a basis for the tangent space. This idea is at the heart of the continuation.

**Fig. 2:**
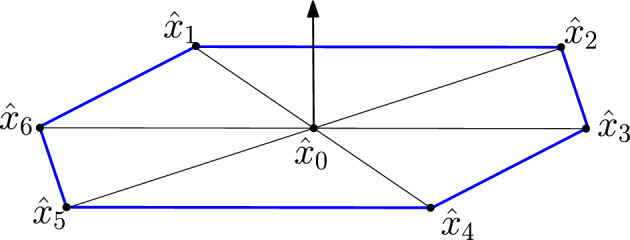
The hexagonal patch with normal vector indicated by an arrow. In this initial configuration, every node except for $\hat{x}_0$ is a boundary node. The boundary edges are blue (Color figure online)

The continuation from $\hat{x}_0$ is done by way of iterative triangulation of the manifold ${\mathcal {M}}$. First, we compute an orthonormal basis of ${\mathcal {T}}_{\hat{x}_0}{\mathcal {M}}$ by applying the Gram-Schmidt process to $\{\hat{\Phi }_1,\hat{\Phi }_2\}$; see Sect. 2.2 for some technical details. Using this orthonormal basis to define a local coordinate system, six vertices of a regular hexagon are computed around $\hat{x}_0$ at a specified distance $\sigma$ from $\hat{x}_0$; see Fig. 2. Let these vertices be denoted $\hat{x}_1$ through $\hat{x}_6$, arranged in counterclockwise order (relative to the local coordinate system) around $\hat{x}_0$. Note that this means

$$\begin{aligned} \hat{x}_i = \hat{x}_0 + \epsilon _{j,1} \hat{\Phi }_1 + \epsilon _{j,2}\hat{\Phi }_2 \end{aligned}$$

for some small $\epsilon _{j,1},\epsilon _{j,2}$, which means that ${\mathcal {G}}(\hat{x}_j)\approx 0$ for $j=1,\dots ,6$. Each of these candidate zeroes $\hat{x}_j$ are then refined by applying Newton’s method to the map

6 $$\begin{aligned} x\mapsto {\mathcal {G}}_j(x) = \left( \begin{array}{c} {\mathcal {G}}(x) \\ \langle \hat{\Phi }_1, x - \hat{x}_j\rangle \\ \langle \hat{\Phi }_2, x - \hat{x}_j\rangle \end{array}\right) . \end{aligned}$$

The two added inner product equations ensure isolation of the solution (hence quadratic convergence of the Newton iterates) and that the Newton correction is perpendicular to the tangent plane. This map can similarly be used to refine the original zero $\hat{x}_0$.

This initial hexagonal “patch” is itself formed by six triangles; see Fig. 2. The continuation algorithm proceeds by selecting one of the boundary vertices (i.e. one of the vertices $\hat{x}_1$ through $\hat{x}_6$) and attempting to “grow” the manifold further. We describe this “growth” phase below. However, first, some terminology. The vertices will now be referred to as *nodes*. An *edge* is a line segment connecting two nodes, and they will be denoted by pairs of nodes: $\{\hat{x}_i,\hat{x}_j\}$ for node $\hat{x}_i$ connected to $\hat{x}_j$. Two nodes are *incident* if they are connected by an edge. A *simplex* is the convex hull of three edges that form a triangle. Once the hexagonal patch is created, the data consists of:

- The nodes $$\hat{x}_0,\dots ,\hat{x}_6$$;
- The “boundary edges” $\{\hat{x}_1,\hat{x}_2\}, \dots ,\{\hat{x}_5,\hat{x}_6\},\{\hat{x}_6,\hat{x}_1\}$;
- The six simplices formed by triangles with $\hat{x}_0$ as one of the nodes.

Two simplices are *adjacent* if they share an edge. An *internal simplex* is a simplex that is adjacent to three other simplices, and it is a *boundary simplex* otherwise. Therefore, the six simplices of the initial patch are all considered boundary since they are adjacent to exactly two others. Similarly, an edge of a simplex can be declared boundary or internal; internal edges are those that are shared with another simplex, and boundary edges are not. A *boundary node* is any node on a boundary edge, a *frontal node* is a boundary node on an edge shared by two simplices, and an *internal node* is a node that is not a boundary node.

**Fig. 3:**
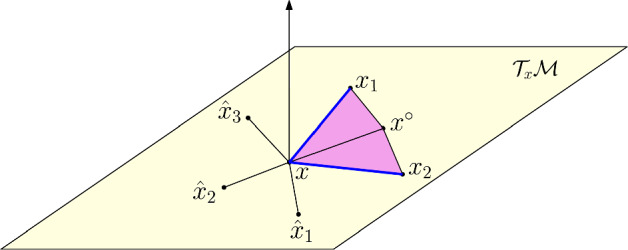
Cartoon diagram of the simplex growth phase. For visualization purposes, we think of $x_1$ and $x_2$ as being in the tangent space ${\mathcal {T}}_x{\mathcal {M}}$; in reality, these should be close to the tangent space but not strictly contained in it. The boundary edges $\{x,x_1\}$ and $\{x,x_2\}$ are blue, and these form a simplices with the interior edge $\{x,x^\circ \}$ that generates the *gap complement direction*. The predictor “fan”, after being mapped back to the tangent space, consists of the vertices $\hat{x}_1$, $\hat{x}_2$ and $\hat{x}_3$. The four new simplices that can be formed, namely $\{x,\hat{x}_1,\hat{x}_2\}$, $\{x,\hat{x}_2,\hat{x}_3\}$, $\{x,\hat{x}_1,x_2\}$ and $\{x,\hat{x}_3,x_1\}$, share the same angle at the *x* vertex

Let *x* be a chosen frontal node (see Sect. 2.2.3 for a general discussion on frontal node selection). By construction, *x* is part of (at least) three distinct edges, two of which connect to boundary nodes, and at least one that connects to an internal node. The algorithm to *grow a simplex* is as follows. See Fig. 3 for a visualization.

1. Compute an orthonormal basis for the tangent space ${\mathcal {T}}_{x}{\mathcal {M}}$.
2. Compute the *(average) gap complement direction*. Let $x^\circ _1,\dots ,x^\circ _m$ denote the list of internal nodes nodes incident to *x*. The gap complement direction is $y_c = -x + \frac{1}{m}\sum _{i=1}^m x_i^\circ$.
3. Let $x_1$ and $x_2$ denote the boundary nodes incident to *x*; form the edge directions $y_1 = x_1 - x$ and $y_2 = x_2 - x$.
4. Orthogonally project $y_c$, $y_1$ and $y_2$ onto the tangent plane ${\mathcal {T}}_x{\mathcal {M}}$. Let these projections be denoted $Py_c$, $Py_1$ and $Py_2$. Introduce a two-dimensional coordinate system on ${\mathcal {T}}_x{\mathcal {M}}$ by way of a “unitary” (see Sect. 2.2) transformation to ${\mathbb {R}}^2$. Let $\tilde{P}y_c$, $\tilde{P}y_1$ and $\tilde{P}y_2$ denote the representatives in ${\mathbb {R}}^2$.
5. In the local two-dimensional coordinate system, compute the counter-clockwise (positive) angle $\theta _1$ required to complete a rotation from $\tilde{P}y_c$ to $\tilde{P}y_1$, and the counter-clockwise (positive) angle $\theta _2$ required for rotation from $\tilde{P}y_c$ to $\tilde{P}y_2$. The *gap angle* $\gamma$ and *orientation* $\rho$ is
  $$\begin{aligned} \gamma = \max \{\theta _1-\theta _2,\theta _2-\theta _1\}, \qquad \rho = \left\{ \begin{array}{ll} 1,&{}\theta _2>\theta _1 \\ 2 &{} \theta _2<\theta _1 \end{array}\right. \end{aligned}$$
6. If $\gamma <\frac{\pi }{6}$ then *close the gap*: add the the triangle formed by the nodes $\{x,x_1,x_2\}$ to the list of simplices, flag it as a boundary simplex, flag *x* as an internal node, and conclude the growth step. Otherwise, proceed to step 7.
7. Generate the predictor “fan” in ${\mathbb {R}}^2$: let $k=\min \left\{ 1,\lfloor 3\theta /\pi \rfloor \right\}$ and define the predictors
  $$\begin{aligned} \tilde{P}\tilde{y}_j = R(j\gamma /k)\tilde{P}y_\rho , \qquad j=1,\dots ,k, \end{aligned}$$
  for $R(\theta )$ the $2\times 2$ counterclockwise rotation matrix through angle $\theta$.
8. Invert the unitary transformation and map $\tilde{P}\tilde{y}_j$ into the tangent plane ${\mathcal {T}}_x{\mathcal {M}}$; let the result be the vectors $P\tilde{y}_j$, $j=1,\dots ,k$.
9. Define predictors $\hat{x}_j = x + \sigma P\tilde{y}_j$ for $j=1,\dots ,k$, where $\sigma$ is a user-specified step size. Refine them using Newton’s method applied to (6), where $\hat{\Phi }_1$ and $\hat{\Phi }_2$ are now the orthonormal basis for ${\mathcal {T}}_x{\mathcal {M}}$.
10. Add the triangles formed by nodes $\{x,\hat{x}_1,\hat{x}_2\}$, $\{x,\hat{x}_2,\hat{x}_3\}$, $\dots$, $\{x,\hat{x}_{k-1},\hat{x}_k\}$. To the list of simplices, flag them as boundary simplices, and flag *x* as an internal node. Do the same with the triangles formed by $\{x,\hat{x}_1,*\}$ and $\{x,\hat{x}_k,*\}$, where $*$ denotes ones of $x_1$ and $x_2$, depending on orientation $\rho$.

At the end of the simplex growth algorithm, one or more boundary simplices is added to the dictionary, and one additional node will be flagged as internal. The structure of this algorithm ensures that each simplex added to the dictionary will be adjacent to exactly two others, indicating that our convention of internal vs. boundary simplices is effective. Once the growth algorithm is complete, another frontal node is selected and the growth phase is repeated. This continues until a sufficient portion of the manifold has been computed (i.e. a user-specified exit condition is reached), or until a Newton’s method correction fails.

### Comments on Implementation

Here we collect some remarks concerning implementation of the finite-dimensional continuation. These comments may be applicable for general continuation problems, but we will often emphasize our specific situation which is continuation of periodic orbits in delay differential equations.

#### Complex Rotations for the Kernel of $D{\mathcal {G}}(x)$

First, generating the kernel elements $\{\hat{\Phi }_1,\hat{\Phi }_2\}$ of $D{\mathcal {G}}(\hat{x})$ for an approximate $\hat{x}$ must be handled in a way that is appropriate to the space ${\mathcal {X}}$. In our problem, ${\mathcal {X}}$ has additional structure: it is a subset of a complexified real vector space equipped with a discrete symmetry. However, in our implementation (that is, in the environment of MATLAB) we work on a generic complex vector space without this symmetry, so when we compute kernel elements using QR decomposition, the computed vectors are not necessarily in ${\mathcal {X}}$. We fix this by performing a complex rotation to put the kernel back into ${\mathcal {X}}$. This can always be done because $\hat{\Phi }_1$ and $\hat{\Phi }_2$, as computed using QR, are always ${\mathbb {C}}$-linearly independent.

#### Orthonormal Basis for the Tangent Space

The next point concerns the “orthonormal basis” of ${\mathcal {T}}_{\hat{x}}{\mathcal {M}}$. Let us be a bit more precise. In our problem, ${\mathcal {X}}$ is a product of the form ${\mathbb {R}}^n\times {\mathcal {V}}$, where ${\mathcal {V}}\subset {\mathbb {C}}^{2M+1}$ consists of vectors $v = (v_{-M},v_{-M+1},\dots ,v_{M-1},v_M)$ such that $v=Sv$, where $Sv =(\overline{v_M},\overline{v_{M-1}},\dots , \overline{v_{-M+1}},\overline{v_{-M}})$. Consider the standard inner product $\langle \cdot ,\cdot \rangle$ on ${\mathbb {C}}^{n+2M+1}$. Once a basis $\{\hat{\Phi }_1,\hat{\Phi }_2\}$ of ${\mathcal {T}}_{\hat{x}}{\mathcal {M}}$ has been computed, these basis vectors can be interpreted as being elements of ${\mathbb {C}}^{n+2M+1}$, and we say that they are orthogonal if $\langle \hat{\Phi }_1, \hat{\Phi }_2\rangle = 0$. It is straightforward to verify that the Gram-Schmidt process applied to this basis produces yet another basis of ${\mathcal {T}}_{\hat{x}}{\mathcal {M}}$ (that is, it does not break the symmetry), and that the new basis is orthonormal with respect to $\langle \cdot ,\cdot \rangle$.

#### Node Prioritization for the Simplex Growth Algorithm

A suitable selection of a frontal node for simplex growth might not be obvious. First, nodes that are more re-entrant (i.e. have many edges incident to them) are generally given higher priority. This is because such nodes are more likely to have the simplex growth algorithm perform the *close the gap* sub-routine. We want to avoid having thin simplices, so prioritizing the closing off of re-entrant nodes takes priority. After this, we typically grow from the “oldest” boundary node.

#### Local Coordinate System in ${\mathbb {R}}^2$ for the Tangent Space

Finally, we must discuss the generation of a two-dimensional coordinate system for ${\mathcal {T}}_x{\mathcal {M}}$. If $\hat{\Phi }_1$ and $\hat{\Phi }_2$ are an orthonormal basis for the tangent space, then the map

$$\begin{aligned} y\mapsto Ly = (\hat{\Phi }_1^*y,\hat{\Phi }_2^*y) \end{aligned}$$

is invertible, where $\hat{\Phi }_i^*$ denotes the conjugate transpose of $\Phi _i$. Writing $Ly = (u,v)\in {\mathbb {C}}^2$ for $y\in {\mathcal {X}}$, one can verify that (*u*, *v*) is the unique solution of

$$\begin{aligned} y = \hat{\Phi }_1 u + \hat{\Phi }_2 v. \end{aligned}$$

In particular, the range of this map is ${\mathbb {R}}^2$ (that is, each of *u* and *v* is real) for our specific problem, where the space ${\mathcal {X}}$ is ${\mathbb {R}}^n\times {\mathcal {V}}$, with ${\mathcal {V}}$ having the symmetry described two paragraphs prior. The inverse map is

$$\begin{aligned} (u,v)\mapsto L^{-1}(u,v) = \hat{\Phi }_1 u + \hat{\Phi }_2 v. \end{aligned}$$

Moreover, this map is unitary in the sense that $\langle Ly, Lw\rangle = \langle y,w\rangle$ for $y,w\in {\mathcal {X}}$, where on the left-hand side we take the standard inner product on ${\mathbb {R}}^2$. It is for these reasons that rotations on ${\mathbb {R}}^2$ performed after the action of *L* are consistent with rotations in the tangent space relative to the gap complement direction.

### Continuation in a Banach Space

Two-parameter continuation can be introduced generally in a Banach space. Let $G:{\mathcal {X}}\rightarrow {\mathcal {Y}}$, with *X* and *Y* being Banach spaces. To connect this formulation with the one of Sect. 2.1, we suppose that there exist projection operators $\pi _{\mathcal {X}}:X\rightarrow {\mathcal {X}}$, $\pi _{\mathcal {Y}}:Y\rightarrow {\mathcal {Y}}$, and associated embeddings $i_{\mathcal {X}}:{\mathcal {X}}\hookrightarrow X$, $i_{\mathcal {Y}}:{\mathcal {Y}}\hookrightarrow Y$, such that ${\mathcal {G}}= \pi _{\mathcal {Y}}G\circ i_{\mathcal {X}}$. In other words, we interpret ${\mathcal {G}}:{\mathcal {X}}\rightarrow {\mathcal {Y}}$ to be a finite-dimensional projection of $G:X\rightarrow Y$. In what follows, we will present a connection between the approximate zeroes of ${\mathcal {G}}$ (i.e. obtained by numerical continuation) and approximate zeroes of *G*.

Abstractly, let $\hat{x}_0,\hat{x}_1,\hat{x}_2\in {\mathcal {X}}$ be the three nodes of a simplex obtained by the numerical continuation scheme for zeroes of ${\mathcal {G}}$. Then we may introduce a coordinate system on this simplex as follows: write each element $\hat{x}_s$ of this simplex as a unique linear combination

7 $$\begin{aligned} \hat{x}_s = \hat{x}_0 + s_1(\hat{x}_1 - \hat{x}_0) + s_2(\hat{x}_2-\hat{x}_0) \end{aligned}$$

for $s = (s_1,s_2)\in \Delta = \{(a,b)\in {\mathbb {R}}^2: 0\le a,b\le 1,\ 0\le a+b\le 1\}$. Let $\{\hat{\Phi }_{j,1},\hat{\Phi }_{j,2}\}$ for $j=0,1,2$ denote an orthonormal basis for the kernel of $D{\mathcal {G}}(\hat{x}_j)$. We can then form the *interpolated kernels*

$$\begin{aligned} \hat{\Phi }_{s,i} = \hat{\Phi }_{0,i} + s_1(\hat{\Phi }_{1,i} - \hat{\Phi }_{0,i}) + s_2(\hat{\Phi }_{2,i}-\hat{\Phi }_{0,i}) \end{aligned}$$

for $i=1,2$. We introduce a nonlinear map $G_s:X\rightarrow Y\times {\mathbb {R}}^2$,

8 $$\begin{aligned} G_s(x) = \left( \begin{array}{c} G(x) \\ \langle \hat{\Phi }_{s,1} ,\pi _{\mathcal {X}}x - \hat{x}_s \rangle \\ \langle \hat{\Phi }_{s,2}, \pi _{\mathcal {X}}x - \hat{x}_s \rangle \end{array}\right) . \end{aligned}$$

The objective is to prove that $G_s$ has a unique zero close to $\hat{x}_s$ for each $s\in \Delta$. If this can be proven, and in fact $G_s$ is $C^k$ for some $k\ge 1$, then one can prove [10] that the the zero set of *G* is a $C^k$, two-dimensional manifold. If this same can be proven for a collection of simplices, then the zero set is globally (i.e. on the union of the cobordant simplicial patches) a $C^k$ manifold over all patches that can be proven; that is, the transition maps between patches are $C^k$.

#### Remark 1

There is a subtle point concerning the relative orientations of the individual kernel elements $\hat{\Phi }_{0,i}$, $\hat{\Phi }_{1,i}$ and $\hat{\Phi }_{2,i}$ that are used to define the interpolation $\hat{\Phi }_{s,i}$. The *validated* continuation (see Sect. 2.4) is unstable, and can even fail outright, if the interpolated kernels $\hat{\Phi }_{s,i}$ vary too much over $s\in \Delta$. If these vectors all lived in the exact same two-dimensional tangent space, this would be very straightforward; we could simply (real) rotate and/or reflect each basis $\{\hat{\Phi }_{1,i},\hat{\Phi }_{2,i}\}$ for $i=1,2$ so that they matched $\{\hat{\Phi }_{1,0},\hat{\Phi }_{2,0}\}$ exactly. However, these vectors live in different tangent spaces, so it is not as easy. The more consistent differential-geometric way to solve the problem would be to parallel transport the tangent basis $\{\hat{\Phi }_{1,0},\hat{\Phi }_{2,0}\}$ to the other two nodes and use these as bases for the tangent spaces there. We do nothing so sophisticated. We merely perform (real) rotations or reflections of the orthonormal bases $\{\hat{\Phi }_{1,i},\hat{\Phi }_{2,i}\}$ in their respective tangent spaces (two-dimensional) for $i=1,2$ in such a way that, in norm, these are as close as possible to $\{\hat{\Phi }_{1,0},\hat{\Phi }_{2,0}\}$. In practice, this has the effect of promoting enough alignment of the bases that proofs are feasible. We emphasize that this alignment process is only needed for the validated continuation; using misaligned tangent bases is not a problem for the steps described in Sect. 2.1.

### Validated Continuation and the Radii Polynomial Approach

In order to prove the existence of a zero of a map $F:X\rightarrow Y$ or, more generally, a family of zeroes of a parameterized map $F_s:X\rightarrow Y$ for parameter *s*, some analytical machinery is needed. If an approximate zero is available, one approach is to use a contraction mapping argument, applied to a Newton-like operator initialized in a neighbourhood of the approximate zero. The *radii polynomial approach* is one such general-purpose proof strategy. It is essentially a Newton–Kantorovich theorem with an approximate derivative, approximate inverse, and domain parametrization. It will be used to connect the approximate zeroes of ${\mathcal {G}}$ with exact zeroes of *G* from the previous section. We state it generally for a family of $s\in \Delta$-dependent maps $F_s:X_1\rightarrow X_2$. We include a short proof for completeness. Some general background and applications of this method can be found in [32] and references cited therein.

#### Theorem 1

Suppose $F_s:X_1\rightarrow X_2$ is differentiable for each $s\in \Delta$, where $X_1$ and $X_2$ are Banach spaces. Let $\hat{x}_s\in X_1$ for all $s\in \Delta$. Suppose there exist for each $s\in \Delta$ a bounded linear operator $A_s^\dagger :X_1\rightarrow X_2$, a bounded and injective linear operator $A_s:X_2\rightarrow X_1$, and non-negative reals $Y_0$, $Z_0$, $Z_1$ and $Z_2=Z_2(r)$ such that

9 $$\begin{aligned} ||A_sF_s(\hat{x}_s)||&\le Y_0 \end{aligned}$$

10 $$\begin{aligned} ||I-A_sA_s^\dagger ||_{B(X_1,X_1)}&\le Z_0 \end{aligned}$$

11 $$\begin{aligned} ||A_s(DF_s(\hat{x}_s)-A_s^\dagger )||_{B(X_1,X_1)}&\le Z_1 \end{aligned}$$

12 $$\begin{aligned} ||A_s(DF_s(\hat{x}_s+\delta ) - DF_s(\hat{x}_s)) ||_{B(X_1,X_1)}&\le Z_2(r), \qquad \forall \delta \in \overline{B_r(\hat{x}_s)}\subset X_1, \end{aligned}$$

for all $s\in \Delta$, where $B_r(\hat{x}_s)$ is the closed ball of radius *r* centered at $\hat{x}_s$, and $||\cdot ||_{B(X_1,X_1)}$ denotes the induced operator norm on $X_1$. Suppose there exists $r_0>0$ such that the *radii polynomial*

$$\begin{aligned} p(r) = rZ_2(r) + (Z_1+Z_0-1)r + Y_0 \end{aligned}$$

satisfies $p(r_0)<0$. Then for each $s\in \Delta$, there is a unique $x_s\in B_{r_0}(\hat{x}_s)$ such that $F_s(x_s)=0$. If $(s,x)\mapsto F_s(x)$ and $s\mapsto A_s$ are $C^k$, then the same is true of $s\mapsto x_s$.

#### Proof

Define the Newton-like operator $T_s(x) = x - A_sF_s(x)$. We will show that $T_s$ is a contraction on $\overline{B_{r_0}(\hat{x}_s)}$, uniformly for $s\in \Delta$. First, write $x\in \overline{B_{r_0}(\hat{x}_s)}$ in the form $x = \hat{x}_s + \delta$ for some $||\delta ||_{\mathcal {X}}\le r$. Then

$$\begin{aligned} ||DT_s(x)||&=||I-A_sDF_s(x)||\\&\le ||I - A_sA_s^\dagger || + ||A_s(A_s^\dagger - DF_s (\hat{x}_s))|| + ||A_s(DF_s(\hat{x}_s) - DF_s(\hat{x}_s+\delta ))||\\&\le Z_0+Z_1+Z_2(r), \end{aligned}$$

Now, using the triangle inequality and the mean-value inequality,

$$\begin{aligned} ||T_s(x)-\hat{x}_s||&\le ||T_s(\hat{x}_s+\delta ) - T_s(\hat{x}_s)|| + ||T_s(\hat{x}_s) - \hat{x}_s||\\&\le r\sup _{t\in [0,1]}||DT_s(\hat{x}_s + t\delta )||_{B(X_1,X_1)} + ||A_sF_s(\hat{x}_s)||\\&\le (Z_0 + Z_1 + Z_2(r))r + Y_0. \end{aligned}$$

Choosing $r=r_0$, the inequality $p(r_0)<0$ implies $||T_s(x)-\hat{x}_s||<r$, so $T_s$ is a self-map on $\overline{B_{r_0}(\hat{x}_s)}$ with its range in the interior. Moreover, since $p(r_0)<0$, we get that $Z_0+Z_1+Z_2(r_0)<1$, which proves that $T_s:\overline{B_{r_0}(\hat{x}_s)}\rightarrow B_{r_0}(\hat{x}_s)$ is a contraction (uniformly in $s\in \Delta$). By the Banach fixed point theorem, $T_s$ has a unique fixed point $x_s\in B_{r_0}(\hat{x}_s)$ for each $s\in \Delta$, and $s\mapsto x_s$ is $C^k$ provided the same is true of $(s,x)\mapsto T_s(x)$. Since $A_s$ is injective, $F_s$ has a unique zero in $B_{r_0}(\hat{x}_s)$ if and only if $T_s$ has a unique fixed point there. $\square$

#### Remark 2

For our problem, injectivity of $A_s$ will always follow from a successful verification of $p(r_0)<0$. See Lemma 4.

In order to apply the theorem, the left-hand sides of the inequalities (9)–(12) are typically majorized using functional-analytic machinery. Ideally, one wants a quantity that is explicitly computable. Such upper bounds are then rigorously computed with interval arithmetic, yielding the bounds $Y_0$ through $Z_2(r)$. In our codes, we use the INTLAB [27] interval arithmetic library. The *computer-assisted proof* then consists of a certificate: the radius $r_0$, and the bounds $Y_0$ through $Z_2(r_0)$, such that $p(r_0)<0$.

Let $\hat{x}_s$ be the convex combination defined by (7) for the simplex nodes $\hat{x}_0$, $\hat{x}_1$ and $\hat{x}_2$. We will say this simplex has been *validated* if we successfully find a radius $r_0$ such that the conditions of the radii polynomial theorem are successful for the nonlinear map $G_s:X\rightarrow Y\times {\mathbb {R}}^2$.

In our implementation, we generate simplices “offline” first. This allows the workload to be distributed across several computers, since the validation step can be restricted to only a subset of the computed simplices. We do not implement a typical refinement procedure, where simplices that fail to validated are split, with more nodes added and corrected with Newton. Rather, we implement an *adaptive refinement* step, which can help with the validation if failure is primarily a result of interval over-estimation. See Sect. 4.2.1.

#### Remark 3

The operators $A_s$ and $A_s^\dagger$ have standard interpretations in terms of the nonlinear map $F_s$. The operator $A_s^\dagger :X_1\rightarrow X_2$ is expected to be an approximation of $DF_s(\hat{x}_s)$, which means that $Z_1$ is a measure of the quality of the approximation. Conversely, $A_s:X_2\rightarrow X_1$ is expected to be an approximation of the *inverse* of $A_s^\dagger$, so that $Z_0$ measures the quality of this approximation. Indirectly, $A_s$ acts as an appoximation of $DF_s(\hat{x}_s)^{-1}$.

### Globalizing the Manifold

Theorem 1 guarantees that the map from the standard simplex to the zero set of $G_s(\cdot )$ is $C^1$. There is then a natural question as to the smoothness of the manifold obtained by gluing together the images of the $C^1$ maps. This is answered in the affirmative in [10], and is primarily a consequence of the numerical data being equal on cobordant simplices.

## Continuation of Periodic Orbits Through (Degenerate) Hopf Bifurcations

In this section, we construct a nonlinear map whose zeroes will encode periodic solutions of a delay differential equation

13 $$\begin{aligned} \dot{y}(t)=f(y(t+\mu _1),\dots ,y(t+\mu _J),\alpha ,\beta ) \end{aligned}$$

for $f:({\mathbb {R}}^n)^{J+1}\times {\mathbb {R}}^2\rightarrow {\mathbb {R}}^n$, with some (positive, negative or zero) constant delays $\mu _1,\dots ,\mu _J$, and distinguished system parameters $\alpha ,\beta$. We will briefly consider ordinary differential equations in Sect. 5 as a special case. We assume that *f* is sufficiently smooth to permit further partial derivative computations.

Following [33], we use the desingularization approach to isolate periodic orbits from (potentially) nearby steady states. This approach allows us to put a large distance (in the sense of a suitable Banach space) between steady states and periodic orbits that arise from Hopf bifurcations. This is exposited in Sect. 3.1, where we also discuss some details concerning non-polynomial nonlinearities.

The next Sect. 3.2 is devoted to the development of a nonlinear map whose zeroes encode periodic orbits of our delay differential equation. In this map, periodic orbits are isolated from fixed points. We present the map abstractly at the level of a function space, and then with respect to a more concrete sequence space.

In Sect. 3.3, we lift the map of the previous section into the scope of two-parameter continuation. We develop an abstract template for the map on a relevant Banach space, define an approximate Fréchet derivative near a candidate zero of this map, and investigate some properties of the Newton-like operator. Specifically, we verify that numerical data corresponding to an approximate *real* periodic orbit, under conditional contraction of the Newton-like operator, will converge to a real periodic orbit.

### Desingularization, Polynomial Embedding and Phase Isolation

We begin by doing a “blowup” around the periodic orbit. Write $y = x+az$, for *x* a candidate equilibrium point and *a* being an auxiliary real scale parameter. Then we get the rescaled vector field

$$\begin{aligned}&\tilde{f}(z_1,\dots ,z_J,x,a,\alpha ,\beta )\\&\quad =\left\{ \begin{array}{ll} a^{-1}(f(x+a z_1,\dots ,x+a z_J,\alpha ,\beta )-f(x,\dots ,x,\alpha ,\beta )),&{} a\ne 0\\ \sum _{j=0}^J d_{y_j}f(x,\dots ,x,\alpha ,\beta )z_j,&{}a=0. \end{array}\right. \end{aligned}$$

We impose $||z||=1$, so that *a* behaves like the relative norm-amplitude of the periodic orbit. The vector field above is $C^{k}$ provided the original function *f* is $C^k$ and *x* is an equilibrium point; that is, $f(x,\dots ,x,\alpha ,\beta )=0$. At this stage we can summarize by saying that our goal is to find a pair (*x*, *z*) such

$$\begin{aligned} f(x,\dots ,x,\alpha ,\beta )&=0\\ \dot{z}(t)&= \tilde{f}(z(t+\mu _1), \dots ,z(t+\mu _J),x,a,\alpha ,\beta ),\\ ||z||&=1 \end{aligned}$$

where *z* is $\omega$-periodic for an unknown period $\omega$; equivalently, the frequency of *z* is $\psi = \frac{2\pi }{\omega }$.

#### Remark 4

It is a common strategy in rigorous numerics, especially for periodic orbits, that time is re-scaled so that the period appears as a parameter in the differential equation. We do not do that here, since this would have the effect of dividing every delay $\mu _j$ by the period. This causes its own set of problems.

In what follows, it will be beneficial for the vector field $\tilde{f}$ to be polynomial. This is because we will make use of a Fourier spectral method, and polynomial nonlinearities in the function space translate directly to convolution-type nonlinearities on the sequence space of Fourier coefficients. While we can make due with non-polynomial nonlinearities, it is greatly simplifies the computer-assisted proof if they are polynomial. To fix this, we generally advocate the use of the *polynomial embedding* technique. The idea is that many analytic, non-polynomial functions are themselves solutions of polynomial ordinary differential equations. The reader may consult [31] for a brief survey of this idea in the context of delay differential equations. See Sect. 7.2 for a specific example.

Applying the polynomial embedding procedure always introduces additional scalar differential equations. If we need to introduce *m* extra scalar equations to get a polynomial vector field, this will also introduce *m* *natural boundary conditions* that fix the initial conditions of the new components. As a consequence, we need to bring in *m* *unfolding parameters* to balance the system. This is accomplished in a problem-specific way; see [31] for some general guidelines and a discussion on the need of these extra unfolding parameters. By an abuse of notation, we assume $\tilde{f}$ is polynomial (that is, the embedding has already been performed), and we write it as

$$\begin{aligned} \tilde{f}(z_1,\dots ,z_J,x,a,\alpha ,\beta ,\eta ), \end{aligned}$$

where $\eta \in {\mathbb {R}}^m$ is a vector representing the unfolding parameter, and we now interpret $\tilde{f}:({\mathbb {R}}^{n+m})^{J+1} \times {\mathbb {R}}^2\rightarrow {\mathbb {R}}^{n+m}$. We then write the natural boundary condition corresponding to the polynomial embedding as

$$\begin{aligned} \theta _{BC}(z(0),x,a,\alpha ,\beta ,\eta )=0\in {\mathbb {R}}^m. \end{aligned}$$

It can also be useful to eliminate non-polynomial parameter dependence from the vector field, especially if the latter has high-order polynomial terms with respect to the state variable *z*. This can often be accomplished by introducing extra scalar variables. For example, if $\tilde{f}$ is

$$\begin{aligned} \alpha e^{-px}z_1 - \beta z_2^2 + \eta _1 \end{aligned}$$

and we want to eliminate the non-polynomial term $e^{-px}$ from the vector field, then we can introduce a new variable $\eta _2$ and impose the equality constraint $0=\eta _2-e^{-px}$. The result is that the vector field becomes

$$\begin{aligned} \alpha \eta _2z_1 - \beta z_2^2 + \eta _1. \end{aligned}$$

Since this operation introduces new variables and additional constraints, we incorporate the constraints as extra components in the natural boundary condition function $\theta _{BC}$ of the polynomial embedding. Since this type of operation will introduce an equal number of additional scalar variables *and* boundary conditions, we will neglect them from the dimension counting.

#### Remark 5

If we want to formalize the embedding process for parameters, we can introduce differential equations for them. Indeed, in the example above, we have $\dot{\eta }_2=0$, and this differential equation can be added to the list of differential equations that result from polynomial embeddings of the original state variable *z*. In this way, we can understand *m* as the total embedding dimension. It should be remarked, however, that in numerical implementation, objects like $\eta _2$ really are treated as scalar quantities.

The final thing we need to take into account is that every periodic orbit is equivalent to a one-dimensional continuum by way of phase shifts. Since our computer-assisted approach to proving periodic orbits is based on Newton’s method and contraction maps, we need to handle this lack of isolation. This can be done by including a *phase condition*. In this paper we will make use of an *anchor condition*; we select a periodic function $\hat{z}$ having the same period as *z*, and we require that

$$\begin{aligned} \int \langle z(s),\hat{z}'(s)\rangle ds = 0. \end{aligned}$$

### Zero-Finding Problem

We are ready to write down a zero-finding problem for our rescaled periodic orbits. First, combining the work of the previous sections, we must simultaneously solve the equations

14 $$\begin{aligned} {\left\{ \begin{array}{ll} \dot{z} = \tilde{f}(z(t+\mu _1),\dots ,z(t+\mu _J),x,a,\alpha ,\beta ,\eta ),&{} \quad \text {(delay differential equations)}\\ \Vert z\Vert = 1, &{}\quad \text {(amplitude condition of scaled orbit)}\\ \int \langle z(s),\hat{z}'(s)\rangle ds=0, &{}\quad \text {(anchor condition)}\\ f(x,\dots ,x,\alpha ,\beta )=0, &{}\quad (x\ \text {is a steady state})\\ \theta _{BC}(z(0),x,a,\alpha ,\beta ,\eta )=0.&{} \quad \text {(embedding boundary condition)} \end{array}\right. } \end{aligned}$$

At this stage, the period $\omega$ of the periodic orbit is implicit. In passing to the spectral representation, we will make it explicit. Define the frequency $\psi = \frac{2\pi }{\omega }$ and expand *z* in Fourier series:

15 $$\begin{aligned} z(t)=\sum _{k\in {\mathbb {Z}}}z_k e^{ik\psi t}. \end{aligned}$$

Recall that for a real (as opposed to complex-valued) periodic orbit, $z_k\in {\mathbb {C}}^{n+m}$ will satisfy the symmetry $z_k = \overline{z_{-k}}$. To substitute (15) into the differential equation in (14), we must examine how time delays transform under Fourier series. Observe

$$\begin{aligned} z(t+\mu ) = \sum _{k\in {\mathbb {Z}}}z_ke^{ik\psi \mu }e^{ik\psi t}, \end{aligned}$$

which means that at the level of Fourier coefficients, a delay of $\mu$ corresponds to a complex rotation

16 $$\begin{aligned} z_k\mapsto (\zeta _\mu (\psi )z)_k\overset{\textrm{def}}{=}e^{ik\psi \mu }z_k. \end{aligned}$$

Note that this operator is linear on $C^{\mathbb {Z}}_{n+m}$ and bounded on $\ell _\nu ^1({\mathbb {C}}^{n+m})$.

Define $$\zeta (\psi ):\ell _\nu ^1({\mathbb {C}}^{n+m})\rightarrow \ell _\nu ^1({\mathbb {C}}^{n+m})^J$$ by

$$\begin{aligned} \zeta (\psi )z = (\zeta _{\mu _1}(\psi )z,\dots ,\zeta _{\mu _J}(\psi )z). \end{aligned}$$

Similarly, we define the derivative *K* on ${\mathbb {C}}^{\mathbb {Z}}$ as $(Kz)_k = kz_k$. We extend this operator to ${\mathbb {C}}^{\mathbb {Z}}_n$ componentwise. Since $\tilde{f}$ is polynomial, subsituting (15) into the first equation of (14) will result in an equation of the form

$$\begin{aligned} \psi iKz = \textbf{f}(\zeta (\psi )z,x,a,\alpha ,\beta ,\eta ) \end{aligned}$$

for a function $\textbf{f}:({\mathbb {C}}^{\mathbb {Z}}_{n+m})^{J}\times {\mathbb {R}}^n \times {\mathbb {R}}\times {\mathbb {R}}^2\times {\mathbb {R}}^m\rightarrow ({\mathbb {C}}^{\mathbb {Z}}_{n+m})$ being a (formal) vector polynomial with respect to Fourier convolution, in the arguments $({\mathbb {C}}^{\mathbb {Z}}_{n+m})^{J}$. Observe that we have abused notation and identified the function *z* in (15) with its sequence of Fourier coefficients. As an example, the nonlinearity $z\mapsto z(t)^2z(t+\mu )$ is transformed in Fourier to the nonlinearity

$$\begin{aligned} z\mapsto (z*z)*(\zeta _\mu (\psi )z). \end{aligned}$$

Now, let $\hat{z}$ be an approximate periodic orbit. We can define new amplitude and phase conditions as functions of *z* and the numerical data $\hat{z}$; see [33]. Then, define a map $G:X\times {\mathbb {R}}^2\rightarrow U$, with $X = \ell _\nu ^1({\mathbb {C}}^{n+m})\times {\mathbb {R}}^n\times {\mathbb {R}}\times {\mathbb {R}}\times {\mathbb {R}}^m$, and $U=K_\nu ({\mathbb {C}}^{n+m})\times {\mathbb {R}}^n\times {\mathbb {C}}\times {\mathbb {C}}\times {\mathbb {C}}^m$, by

17 $$\begin{aligned} G(z,x,a,\psi ,\eta ,(\alpha ,\beta ))&=\left( \begin{array}{c} -\psi iKz + \textbf{f}(\zeta (\psi )z,x,a,\alpha ,\beta ,\eta ) \\ f(x,\dots ,x,\alpha ,\beta ) \\ \langle z,K^2\hat{z}\rangle - 1 \\ \langle z,iK\hat{z}\rangle \\ \theta _{BC}(\sum _k z_k,x,a,\alpha ,\beta ,\eta ) \end{array}\right) . \end{aligned}$$

Let *X* be equipped with the norm

18 $$\begin{aligned} ||(z,x,a,\psi ,\eta )||=\max \{||z||_\nu ,|x|,|a|,|\psi |,|\eta | \}, \end{aligned}$$

where all space norms are selected a priori *and could be distinct*. That is, we allow for the possibility of a refined weighting^1^ of the norms being used. Then *X* is a Banach space, and the same is true for *U* when equipped with an analogous norm, replacing $||\cdot ||_\nu$ with $||\cdot ||_{\nu ,K}$.

It will sometimes be convenient to compute norms on the ${\mathbb {R}}^{n+m+4}$-projection of *X*. In this case, if $u=(z,y)\in X$ and $y\in {\mathbb {R}}^{n+m+4}$ is represented (ismorphically) as $y=(x,a,\psi ,\eta )\in {\mathbb {R}}^n\times {\mathbb {R}}\times {\mathbb {R}}\times {\mathbb {R}}^m$, then we define $||y||=\max \{|x|,|a|,|\psi |,|\eta |\}$, where any weighting is, again, implicit. Then $||(z,y)||= \max \{||z||_\nu ,||y||\}$.

Introduce $V=\text{ Symm }(\ell _\nu ^1({\mathbb {C}}^{n+m}))\times {\mathbb {R}}^n \times {\mathbb {R}}\times {\mathbb {R}}\times {\mathbb {R}}^m$. Any zero of *F* in the space *V* uniquely corresponds to a *real* periodic orbit of (13) by way of the Fourier expansion (15) and the blow-up coordinates $y = x+az$. Moreover, the restriction of *F* to *V* has range in $W={{\,\textrm{Symm}\,}}(K_\nu ({\mathbb {C}}^{n+m}))\times {\mathbb {R}}^n\times {\mathbb {R}}\times {\mathbb {R}}\times {\mathbb {R}}^m$. Each of *V* and *W* are Banach spaces over the reals, and so from this point on we work with the restriction $F:V\rightarrow W$.

### Finite-Dimensional Projection

To set up the rigorous numerics and the continuation, we need to define projections of *V* and *W* onto suitable finite-dimensional vector spaces. Let $\pi ^M:\ell _\nu ^1({\mathbb {C}}^{n+m})\rightarrow \ell _\nu ^1({\mathbb {C}}^{n+m})$ denote the projection operator

$$\begin{aligned} (\pi ^Mz)_k=\left\{ \begin{array}{ll} z_k,&{}|k|\le M \\ 0 &{} |k|>M, \end{array}\right. \end{aligned}$$

and $\pi ^\infty = I_{\ell _\nu ^1({\mathbb {C}}^{n+m})}-\pi ^M$ its complementary projector. Consider the finite-dimensional vector space

$$\begin{aligned} V^M\overset{\textrm{def}}{=}\pi ^M \big ({{\,\textrm{Symm}\,}}({\mathbb {C}}^{\mathbb {Z}}_{n+m})\big )\times {\mathbb {R}}^n\times {\mathbb {R}}\times {\mathbb {R}}\times {\mathbb {R}}^m, \end{aligned}$$

and extend the projection to a map $\pi ^M:V\rightarrow V^M$ as follows:

$$\begin{aligned} \pi ^M(z,x,a,\psi ,\eta ) = (\pi ^M z,x,a,\psi ,\eta ). \end{aligned}$$

Now define a map ${\mathcal {G}}:V^M\times {\mathbb {R}}^2\rightarrow V^M$

19 $$\begin{aligned} {\mathcal {G}}(z,x,a,\psi ,\eta ,(\alpha ,\beta ))&=\pi ^M G(z,x,a,\psi , \eta ,(\alpha ,\beta )). \end{aligned}$$

${\mathcal {G}}$ is well-defined and smooth, and we have ${\mathcal {G}}= \pi ^M G\circ i_{V^M}$, where $i_{V^M}:V^M\hookrightarrow V$ is the natural inclusion map. Therefore, this definition of the projection of *G* is consistent with the abstract set-up of Sect. 2.3.

### A Reformulation of the Continuation Map

It will be convenient to identify $V\times {\mathbb {R}}^2$ and $V^M\times {\mathbb {R}}^2$ respectively with isomorphic spaces

$$\begin{aligned} V\times {\mathbb {R}}^2&\sim \pi ^M\big ({{\,\textrm{Symm}\,}}\big (\ell _\nu ^1({\mathbb {C}}^{n+m})\big )\big ) \times {\mathbb {R}}^{n+m+4}\times \pi ^\infty \big ({{\,\textrm{Symm}\,}}\big (\ell _\nu ^1 ({\mathbb {C}}^{n+m})\big )\big )\overset{\textrm{def}}{=}\Omega \\ V^M\times {\mathbb {R}}^2&\sim \pi ^M\big ({{\,\textrm{Symm}\,}}\big (\ell _\nu ^1({\mathbb {C}}^{n+m})\big )\big ) \times {\mathbb {R}}^{n+m+4}\overset{\textrm{def}}{=}\Omega ^M \end{aligned}$$

There is a natural embedding $(z^M,\rho )\mapsto (z^M,\rho ,0)$ of $\Omega ^M$ into $\Omega$. The isomorphism of *V* with $\Omega$ is given by

$$\begin{aligned} (z,x,a,\psi ,\eta ,(\alpha ,\beta ))\mapsto \big (\pi ^M z, (x,a,\psi ,\eta ,\alpha ,\beta ),\pi ^\infty z \big ). \end{aligned}$$

$\Omega ^M$ is finite-dimensional, and as such our language will sometimes reinforce this by describing matrices whose columns are elements of $\Omega ^M$. This should be understood “up to isomorphism”. The purpose of the isomorphism of $V\times {\mathbb {R}}^2$ with $\Omega$ is to symbolically group all of the finite-dimensional objects together.

Given $\hat{u}_j = (\hat{z}_j,\hat{\rho }_j)\in \Omega ^M$ for $j=0,1,2$, let $\hat{\Phi }_j$ be a matrix whose columns are a basis for the kernel of $DF^M(\hat{z}_j,\hat{\rho }_j)$, and are therefore elements of $V^M\times {\mathbb {R}}^2\sim \Omega ^M$. For $s\in \Delta$, let $\hat{u}_s$ and $\hat{\Phi }_s$ be the usual interpolations of the elements $\hat{u}_j$ and bases $\hat{\Phi }_j$ for $j=0,1,2$.

The continuation map $G_s$ of (8) could now be defined for our periodic orbit function *G*. However, it will be convenient in our subsequent discussions concerning the radii polynomial approach to re-interpret the codomain of $G_s$ as being

$$\begin{aligned} \tilde{\Omega }\overset{\textrm{def}}{=}\pi ^M\text{ Symm }(K_\nu ({\mathbb {C}}^{n+m})) \times {\mathbb {R}}^{n+m+4}\times \pi ^\infty \text{ Symm }(K_\nu ({\mathbb {C}}^{n+m})). \end{aligned}$$

Specifically, this will make it a bit easier to define an approximate inverse of $DG_s(\hat{u}_s)$. The codomain of $G_s$ is

$$\begin{aligned} W\times {\mathbb {R}}^2&= (\text{ Symm }(K_\nu ({\mathbb {C}}^{n+m}))\times {\mathbb {R}}^n \times {\mathbb {R}}\times {\mathbb {R}}\times {\mathbb {R}}^m)\times {\mathbb {R}}^2\\&\sim \text{ Symm }(K_\nu ({\mathbb {C}}^{n+m}))\times {\mathbb {R}}^{n+m+4}\\&\sim \tilde{\Omega }, \end{aligned}$$

where the isomorphisms can be realized by permuting the relevant components of $G_s$ and splitting the Fourier space into direct sums. For $(z^M,\rho ,z^\infty )\in \Omega$, a suitable isomorphic representation of $G_s$ is given by ${\mathcal {F}}_s:\Omega \rightarrow \tilde{\Omega }$,

20 $$\begin{aligned} {\mathcal {F}}_s(z^M,\rho ,z^\infty )&=\left( \begin{array}{c} -\psi i K z^M + \pi ^M\textbf{f}(\zeta (\psi )(z^M+z^\infty ),\rho ) \\ \textbf{J}_s(z^M,\rho ,z^\infty )\\ -\psi i Kz^\infty + \pi ^\infty \textbf{f}(\zeta (\psi )(z^M+z^\infty ),\rho ) \end{array}\right) \overset{\textrm{def}}{=}\left( \begin{array}{c} {{\mathcal {F}}}_s^{(1)} \\ {{\mathcal {F}}}_s^{(2)}\\ {{\mathcal {F}}}_s^{(3)} \end{array}\right) , \end{aligned}$$

21 $$\begin{aligned} \textbf{J}_s(z^M,\rho ,z^\infty )&=\left( \begin{array}{c} f(x,\dots ,x,\alpha ,\beta ) \\ \langle z^M,K^2\hat{u}_s\rangle - 1 \\ \langle z^M , iK \hat{u}_s\rangle \\ \theta _{BC}(\sum _k (z^M_k+z^\infty _k),x,a,\alpha ,\beta ,\eta ) \\ \langle \hat{\Phi }_{s,1}, u^M\rangle - \hat{c}_{s,1}\\ \langle \hat{\Phi }_{s,2}, u^M\rangle - \hat{c}_{s,2} \end{array}\right) , \ \ u^M = \left( \begin{array}{c} z^M \\ \rho \end{array}\right) \in \Omega ^M, \end{aligned}$$

where $\rho = (x,a,\psi ,\eta ,\alpha ,\beta )$, and the $\hat{c}_{s,j}$ for $j=1,2$ are interpolations

22 $$\begin{aligned} \hat{c}_{s,j}=\langle \hat{\Phi }_{0,j},\hat{u}_0\rangle + s_1(\langle \hat{\Phi }_{1,j},\hat{u}_1\rangle -\langle \hat{\Phi }_{0,j},\hat{u}_0\rangle ) + s_2(\langle \hat{\Phi }_{2,j},\hat{u}_2\rangle - \langle \hat{\Phi }_{0,j},\hat{u}_0\rangle ). \end{aligned}$$

#### Remark 6

Strictly speaking, the final two components of $\textbf{J}_s$ are not the “standard” ones from (8). The inner product $\langle \Phi _{s,j},\pi _{\mathcal {X}}x - \hat{x}_s\rangle$ in the latter results in quadratic terms in *s*, while the ones in (21) are *s*-linear. The impact of this change is that, theoretically, the Newton correction is not strictly in the direction orthogonal to the interpolation of the tangent planes. This change has no theoretical bearing on the validated continuation, and is done only for ease of computation: it is easier to compute derivatives of linear functions than nonlinear ones.

#### Remark 7

We have abused notation somewhat, since now we interpret $\textbf{f}$ as a map

$$\begin{aligned} \textbf{f}:{{\,\textrm{Symm}\,}}(\ell _\nu ^1({\mathbb {C}}^{n+m}))\times {\mathbb {R}}^{n+m+4} \rightarrow {{\,\textrm{Symm}\,}}(K_\nu ({\mathbb {C}}^{n+m})). \end{aligned}$$

It acts trivially with respect to the variable $\psi$. Also, we emphasize that $G_s$ depends on the numerical interpolants $\hat{u}_s$ and $\hat{\Phi }_s$.

Since Theorem 1 is stated with respect to general Banach spaces, the validated continuation approach applies equally to the representation ${\mathcal {F}}_s:\Omega \rightarrow \tilde{\Omega }$ of $G_s:V\rightarrow W$. The only thing we need to do is specify a compatible norm on $\Omega$. This is straightforward: for $(z^M,\rho ,z^\infty )\in \Omega$ and $\rho = (x,a,\psi ,\eta ,\alpha ,\beta )$, a suitable norm is

$$\begin{aligned} ||(z^M,\rho ,z^\infty )||_\Omega =\max \{||z^M+z^\infty ||_\nu ,|x|,|a|,|\psi |,|\eta |,|\alpha |,|\beta |\}, \end{aligned}$$

where the norms on the components of $\rho$ are the same^2^ as the ones appearing in (18). With this choice, $||\cdot ||_\Omega$ is equivalent to the induced max norm on $X\times {\mathbb {R}}^2$, with *X* equipped with the norm (18) and ${\mathbb {R}}^2$ the $\infty$-norm.

### Construction of $A_s^\dagger$ and $A_s$

Write $\hat{u}_s = (\hat{z}_s,\hat{x}_s,\hat{\psi }_s,\hat{a}_s, \hat{\alpha }_s,\hat{\beta }_s)$, for $s\in \Delta$. Denote the three vertices of $\Delta$ as $s_0 = (0,0)$, $s_1 = (1,0)$ and $s_2 =(0,1)$. Introduce an approximation of ${\mathcal {F}}_s$ as follows:

23 $$\begin{aligned} \tilde{{\mathcal {F}}}_s(z^M,\rho ,z^\infty )&=\left( \begin{array}{c} -\psi i Kz^M + \pi ^M\textbf{f}(\zeta (\psi )z^M,\rho ) \\ \textbf{J}_s(z^M,\rho ,z^\infty )\\ -\psi iKz^\infty \end{array}\right) \overset{\textrm{def}}{=}\left( \begin{array}{c} \tilde{{\mathcal {F}}}_s^{(1)} \\ \tilde{{\mathcal {F}}}_s^{(2)}\\ \tilde{{\mathcal {F}}}_s^{(3)} \end{array}\right) . \end{aligned}$$

Formally, $\tilde{{\mathcal {F}}}_s$ approximates ${\mathcal {F}}_s$ in the Fourier tail by neglecting the nonlinear terms, leaving only the part coming from the differentiation operator.

#### Proposition 2

$D\tilde{{\mathcal {F}}}_s(\hat{u}_s)$ has the representation

24 $$\begin{aligned} D\tilde{{\mathcal {F}}}_s(\hat{u}_s)&=\left( \begin{array}{ccc} D_1\tilde{{\mathcal {F}}}_s^{(1)}(\hat{u}_s)&{} D_2\tilde{{\mathcal {F}}}_s^{(1)}(\hat{u}_s)&{}0 \\ D_1\tilde{{\mathcal {F}}}_s^{(2)}(\hat{u}_s) &{} D_2\tilde{{\mathcal {F}}}_s^{(2)}(\hat{u}_s) &{} D_3\tilde{{\mathcal {F}}}_s^{(2)}(\hat{u}_s) \\ 0&{}0&{}D_3\tilde{{\mathcal {F}}}_s^{(3)}(\hat{u}_s) \end{array}\right) \overset{\textrm{def}}{=}A_s^\dagger . \end{aligned}$$

#### Proof

Since ${\mathcal {F}}_s^{(1)}$ does not depend on $z^\infty$, the upper-right block of $D{\mathcal {F}}_S(\hat{u}_s)$ is the zero map. Similarly, ${\mathcal {F}}_s^{(3)}$ does not depend on either $z^M$ or $\rho$, so the finite-dimensional blocks in the $z^\infty$ (bottom) row are zero. $\square$

The upper left $2\times 2$ block is equivalent to a finite-dimensional matrix operator. In particular, $D_2\tilde{{\mathcal {F}}}_s^{(2)}(\hat{u}_s)$ is real. Also,

$$\begin{aligned} D_3\tilde{{\mathcal {F}}}_s^{(3)}(\hat{u}_s) = -\hat{\psi }_s iK\pi ^\infty \end{aligned}$$

is invertible, with

$$\begin{aligned} D_3\tilde{{\mathcal {F}}}_s^{(3)}(\hat{u}_s)^{-1} =i(\hat{\psi }_s)^{-1}(K\pi ^\infty )^{-1}, \end{aligned}$$

where $((K\pi ^\infty )^{-1}z)_k \overset{\textrm{def}}{=}\frac{1}{k}z_k$ for $|k|\ge M+1$. Suppose we can explicitly compute $S_j\in {\mathbb {R}}^{(m+n+4)\times (m+n+4)}$ and $P_j,Q_j,R_j$ complex matrices for $j=0,1,2$ such that

25 $$\begin{aligned} \left( \begin{array}{cc} P_j&{}Q_j\\ R_j&{}S_j \end{array}\right) \left( \begin{array}{cc} D_1\tilde{{\mathcal {F}}}_{s_j}^{(1)}(\hat{u}_{s_j})&{} D_2\tilde{{\mathcal {F}}}_{s_j}^{(1)}(\hat{u}_{s_j})\\ D_1\tilde{{\mathcal {F}}}_{s_j}^{(2)}(\hat{u}_{s_j}) &{} D_2\tilde{{\mathcal {F}}}_{s_j}^{(2)}(\hat{u}_{s_j}) \end{array}\right) \approx I_{\Omega ^M}. \end{aligned}$$

We can then prove the following lemma.

#### Lemma 3

For $s=(s^{(1)},s^{(2)})\in \Delta$, define matrix interpolants $P_s=P_1 + s^{(1)}(P_2-P_1) + s^{(2)}(P_3-P_1)$, and analogously define interpolants $Q_s$, $R_s$ and $S_s$. Introduce a family of operators $A_s$ as follows:

26 $$\begin{aligned} A_s&=\left( \begin{array}{ccc} P_s&{}Q_s&{}-Q_sD_3\tilde{{\mathcal {F}}}_s^{(2)}(\hat{u}_s)i (\hat{\psi }_s)^{-1}(K\pi ^\infty )^{-1} \\ R_s&{}S_s&{}-S_sD_3\tilde{{\mathcal {F}}}_s^{(2)}(\hat{u}_s)i (\hat{\psi }_s)^{-1}(K\pi ^\infty )^{-1} \\ 0&{}0&{}i(\hat{\psi }_s)^{-1}(K\pi ^\infty )^{-1} \end{array}\right) . \end{aligned}$$

Suppose for $j=1,2,3$, $S_j$ is real and, as maps, $P_j$, $Q_j$ and $R_j$ are equivalent to

$$\begin{aligned}&P_j\,:\,\pi ^M({{\,\textrm{Symm}\,}}({\mathbb {C}}^{\mathbb {Z}}_{n+m}))\rightarrow \pi ^M({{\,\textrm{Symm}\,}}({\mathbb {C}}^{\mathbb {Z}}_{n+m}))\\&Q_j\,:\,{\mathbb {R}}^{m+n+4}\rightarrow \pi ^M({{\,\textrm{Symm}\,}}({\mathbb {C}}^{\mathbb {Z}}_{n+m}))\\&R_j\,:\,\pi ^M({{\,\textrm{Symm}\,}}({\mathbb {C}}^{\mathbb {Z}}_{n+m}))\rightarrow {\mathbb {R}}^{n+m+4}. \end{aligned}$$

Then $A_s:\tilde{\Omega }\rightarrow \Omega$ is well-defined.

#### Proof

One can show $A_s:\tilde{\Omega }\rightarrow \Omega$ is well-defined using the fact that each of $P_s$, $Q_s$ and $R_s$ is a real convex combination of maps to/from appropriate symmetric spaces, and noticing that $iK\pi ^\infty$ (and hence its inverse) satisfy the symmetry $(iK\pi ^\infty z)_k = \overline{(iK\pi ^\infty z)_{-k}}$ $\square$

The point here is that, due to (25), we have

$$\begin{aligned} A_{s_j}\approx D\tilde{{\mathcal {F}}}_{s_j}(\hat{u}_{s_j})^{-1} \approx (A^\dagger _{s_j})^{-1} \end{aligned}$$

for $j=0,1,2$, and if the interpolation points $\hat{u}_j$ are close together, we expect $D\tilde{{\mathcal {F}}}_s(\hat{u}_{s_j})$ to be invertible for all $s\in \Delta$, and $D\tilde{{\mathcal {F}}}_s(\hat{u}_{s_j})^{-1}\approx A_s$.

#### Remark 8

Checking the conditions of Lemma 3 amounts to verifying conjugate symmetries of the matrices $P_j$, $Q_j$ and $R_j$. Numerical rounding makes this a nontrivial task, so we generally post-process the numerically computed matrices to impose these symmetry conditions.

## Technical Bounds for Validated Continuation of Periodic Orbits

Based on the previous section, we define a Newton-like operator $T_s:\Omega \rightarrow \Omega$

27 $$\begin{aligned} T_s(u) = u - A_s{\mathcal {F}}_s(u), \end{aligned}$$

for $s\in \Delta$. As described in Sect. 2.4, our goal is to prove that $T_s$ is a uniform (for $s\in \Delta$) contraction in a closed ball centered at $\hat{u}_s$ using Theorem 1. If $A_s$ can be proven (uniformly in *s*) injective, this will prove the existence of a unique zero of ${\mathcal {F}}_s(\cdot )$ close to $\hat{u}_s$, thereby validating the simplex and proving the smooth patch of our manifold.

In Sect. 4.1, we will demonstrate how the bound $Z_0$ of the radii polynomial approach can be used to obtain a proof of uniform (in *s*) injectivity of the operator $A_s$. We then provide some detailed discussion concerning general-purpose implementation of the bounds *Y* and *Z* in Sect. 4.2 through to Sect. 4.5.

### Injectivity of $A_s$

The injectivity of $A_s$ is a consequence of the successful identification of bounds $Z_0$ of Theorem 1 and the negativity of the radii polynomial. In particular,

#### Lemma 4

Suppose $||I-A_sA_s^\dagger ||_{B(\Omega ,\Omega )}\le Z_0$ for all $s\in \Delta$, with the operators $A_s$ and $A_s^\dagger$ of Sect. 3.5. If $Z_0<1$, then $A_s$ is injective for $s\in \Delta$.

#### Proof

First, observe $A_s$ has non-trivial kernel if and only if there exists $u\in \Omega ^M$ such that $A_s u = 0$. This is a consequence of the structure of the operator and injectivity of $(K\pi ^\infty )^{-1}$. Therefore, it suffices to verify the injectivity of the restriction $\textbf{A}_s = A_s|_{\Omega ^M}$. Define $\textbf{A}_s^\dagger = A_s^\dagger |_{\Omega ^M}$. By definition of the norm on $\Omega$, we have $||I-\textbf{A}_s\textbf{A}_s^\dagger ||_{B(\Omega ^M,\Omega ^M)}\le Z_0<1$ for all $s\in \Delta$. By Neumann series, it follows that $\textbf{A}_s\textbf{A}_s^\dagger$ is boundedly invertible, which implies $\textbf{A}_s$ is surjective. Since $\Omega ^M$ is finite-dimensional, $\textbf{A}_s$ is also injective. $\square$

#### Corollary 5

If the radii polynomial satisfies $p(r_0)<0$ for some $r_0>0$, then $A_s$ is injective for $s\in \Delta$.

### The Bound $Y_0$

To begin, expand the product $A_s{\mathcal {F}}_s(\hat{u}_s)$. We get

$$\begin{aligned} A_s{\mathcal {F}}_s(\hat{u}_s)&=\left( \begin{array}{c} P_s{\mathcal {F}}_s^{(1)}(\hat{u}_s) + Q_s{\mathcal {F}}_s^{(2)}(\hat{u}_s) - Q_sD_3\tilde{{\mathcal {F}}}_s^{(2)}(\hat{u}_s)i(\hat{\psi }_s)^{-1} (K\pi ^\infty )^{-1}{\mathcal {F}}_s^{(3)}(\hat{u}_s) \\ R_s{\mathcal {F}}_s^{(1)}(\hat{u}_s) + S_s{\mathcal {F}}_s^{(2)} (\hat{u}_s) - S_sD_3\tilde{{\mathcal {F}}}_s^{(2)}(\hat{u}_s)i (\hat{\psi }_s)^{-1}(K\pi ^\infty )^{-1}{\mathcal {F}}_s^{(3)}(\hat{u}_s)\\ i(\hat{\psi }_s)^{-1}(K\pi ^\infty )^{-1}{\mathcal {F}}_s^{(3)}(\hat{u}_s) \\ \end{array}\right) \end{aligned}$$

Remark that ${\mathcal {F}}_s^{(3)}(\hat{u}_s)$ has range in a finite-dimensional subspace of $\Omega$; specifically, it will be in the part of $\Omega$ such that the components in ${\mathbb {C}}^{\mathbb {Z}}_{n+m}$ with index (in absolute value) greater than $Md+1$ are zero, where *d* is the maximum degree of the (convolution) polynomial $\textbf{f}$. As such, $A_s{\mathcal {F}}_s(\hat{u}_s)$ is explicitly computable.

In practice, we must compute an enclosure of the norm $||A_s{\mathcal {F}}_s(\hat{u}_s)||$ for all $s\in \Delta$. This is slightly less trivial. We accomplish this using a first-order Taylor expansion with remainder. For the function $(s,u)\mapsto {\mathcal {F}}_s(u)$, denote by $\partial _s$ the Fréchet derivative with respect to *s*, and *D* the derivative with respect to *u*. Given the interpolants $\hat{u}_s=(\hat{v}_s,\hat{\rho }_s)$ and $\hat{\Phi }_{s,j}$, let $\hat{u}'$ and $\hat{\Phi }_{j}'$ denote their Fréchet derivatives with respect to *s*. Also, denote $\hat{c}_{s,j}'= \partial _s \hat{c}_{s,j}$, for $\hat{c}_{s,j}$ defined in (22). Note that these derivatives are constant, since the interpolants are linear in *s*. Then

$$\begin{aligned} {\mathcal {F}}_s(\hat{u}_s) = {\mathcal {F}}_{s_0}(\hat{u}_0) + \big (\partial _s{\mathcal {F}}_{s_0}(\hat{u}_0) + D{\mathcal {F}}_{s_0}(\hat{u}_0)\hat{u}'\big )s + \frac{1}{2}{\mathcal {R}}, \end{aligned}$$

where the remainder term ${\mathcal {R}}$ will be elaborated upon momentarily. The product of the linear-order terms with $A_s$ results in a function that is componentwise quadratic in $s\in \Delta$, for which we can efficiently compute an upper bound on the norm. The derivative $D{\mathcal {F}}_s(\hat{u}_s)$ is implementable, and will be further discussed in Sect. 4.3. As for the $\partial _s$ term,

$$\begin{aligned} \partial _s{\mathcal {F}}_s(u)&=\left( \begin{array}{c} 0\\ \partial _s\textbf{J}_s(u)\\ 0 \end{array}\right) , \ \ \partial _s\textbf{J}_s(u)=\left( \begin{array}{c} 0 \\ \langle z,K^2\hat{z}'\rangle \\ \langle z,iK\hat{z}'\rangle \\ 0 \\ \langle \hat{\Phi }_1',u\rangle - \hat{c}_{s,1}'\\ \langle \hat{\Phi }_2',u\rangle - \hat{c}_{s,2}' \end{array}\right) , \end{aligned}$$

where $$u=(z,\rho )$$ and $$\hat{u}_s = (\hat{z}_s,\hat{\rho }_s)$$.

The remainder ${\mathcal {R}}$ is bounded by the norm of the second Fréchet derivative of $s\mapsto {\mathcal {F}}_s(\hat{u}_s)$, uniformly over $s\in \Delta$. For each $s\in \Delta$, let this second derivative be denoted $\textbf{D}^2{\mathcal {F}}_s(\hat{u}_s)$. Then

28 $$\begin{aligned} \textbf{D}^2{\mathcal {F}}_s(\hat{u}_s)&=\partial _s^2{\mathcal {F}}_s(\hat{u}_s)[e_1,e_2] + \partial _s D{\mathcal {F}}_s(\hat{u}_s)[\hat{u}'e_1,e_2] + D\partial _s {\mathcal {F}}_s(\hat{u}_s)[e_1,\hat{u}' e_2]\nonumber \\&\quad +D^2{\mathcal {F}}_s(\hat{u}_s)[\hat{u}'e_1,\hat{u}'e_2]. \end{aligned}$$

where $e_1$ and $e_2$ denote the first and second (factor) projection maps on ${\mathbb {R}}^2\times {\mathbb {R}}^2$. The derivative $D^2{\mathcal {F}}_s$ will be discussed in Sect. 4.3. At this stage, we need only mention that it acts bilinearly on $\hat{u}$, and the latter is proportional in norm to the step size $\sigma$ of the continuation scheme, so the $D^2{\mathcal {F}}_s$ term will be order $\sigma ^2$. As for the derivatives involving $\partial _s$, most of the components are zero as evidenced by the previous calculation of $\partial _s{\mathcal {F}}_s(z,\rho )$, and it suffices to compute the relevant derivatives of $\textbf{J}_s$. We have

$$\begin{aligned} \partial _s^2\textbf{J}_s(\hat{u}_s)[t_1,t_2]&=0 ,\ \ D\partial _s\textbf{J}_s(\hat{u}_s)[t,h]=\left( \begin{array}{c} 0 \\ \langle h_z,K^2\hat{z}'t\rangle \\ \langle h_z,iK\hat{z}'t\rangle \\ 0 \\ \langle \hat{\Phi }_1't,h\rangle \\ \langle \hat{\Phi }_2't,h\rangle \end{array}\right) , \end{aligned}$$

with $(t,h)\in {\mathbb {R}}^2\times \Omega$, $h = (h_z,h_\rho )$. In terms of the step size, $\partial _s^2\textbf{J}_s(\hat{u}_s)$ is order $\sigma ^2$, while $D\partial _s{\mathcal {F}}_s(\hat{u}_s)$ is order $\sigma$. However, in (28), the latter term is multiplied by $\hat{z}' = O(\sigma )$. Therefore, as expected, the remainder ${\mathcal {R}}$ is quadratic with respect to step size. We therefore compute

$$\begin{aligned} Y_0\ge ||A_s({\mathcal {F}}_s(\hat{u}_s) - 2^{-1}{\mathcal {R}})|| +\frac{1}{2}||A_s{\mathcal {R}}||. \end{aligned}$$

Since $||R||=O(\sigma ^2)$, the bound can be tempered quadratically by reducing the step size. The caveat is that if $||A_s||$ and/or ${\mathcal {F}}_s$ has large quadratic terms, it might still be necessary to take small steps.

#### Remark 9

Directly computing the norm $||A_s{\mathcal {R}}||$ would require a general-purpose implementation of the second derivative $D^2{\mathcal {F}}_s(\hat{u}_s)$; see (28). As we have stated previously, such an implementation would be rather complicated. Therefore, in practice, we perform another level of splitting; namely, we use the bound

$$\begin{aligned} ||A_s {\mathcal {R}}||\le ||A_s\left( \textbf{D}^2{\mathcal {F}}_s(\hat{u}_s) - D^2{\mathcal {F}}_s(\hat{u}_s)[\hat{u}'e_1,\hat{u}'e_2] \right) || + ||A_sD^2{\mathcal {F}}_s(\hat{u}_s)[\hat{u}'e_1,\hat{u}'e_2]||. \end{aligned}$$

The first term on the right of the inequality is explicitly implementable using only the derivatives of $\textbf{J}_s$ and the finite blocks of $A_s$. For the second term, we use the bound $||A_s||\cdot ||D^2{\mathcal {F}}_s(\hat{u}_s)[\hat{u}'e_1,\hat{u}'e_2]||$ which, while not optimal, is implementable and good enough for our purposes.

#### Adaptive Refinement

In continuation, the size of the $Y_0$ bound is severely limited by the step size. To distribute computations, we often want to compute the manifold first and then validate patches *a posteriori*. However, once the manifold has been computed, adjusting and re-computing patches of the manifold with smaller step sizes becomes complicated due to the need to ensure that cobordant simplices have matched data, as discussed in Sect. 2.5. When the $Y_0$ bound is too large due to interval arithmetic over-estimation, we can circumvent this by using *adaptive refinement* on the relevant simplex. Formally, we subdivide the simplex into four, using the interpolated zeroes at the nodes of the original simplex to define the data at the nodes of the four new ones. The result is that cobordant data still matches, allowing for globalization of the manifold. The advantage of this approach is that it can be safely done in a distributed manner; adaptively refining one simplex does not require re-validating any of its neighbours.

#### The Bound $Z_0$

The product $A_sA_s^\dagger$ is block diagonal. Indeed, $(I-A_sA_s^\dagger )|_{\pi ^\infty \Omega } = 0$, whereas

$$\begin{aligned} (I-A_sA_s^\dagger )|_{\Omega ^M} = I_{\Omega ^M} - \left( \begin{array}{cc} P_s&{}Q_s\\ R_s&{}S_s \end{array}\right) \left( \begin{array}{cc} D_1\tilde{{\mathcal {F}}}_{s}^{(1)}(\hat{u}_{s})&{} D_2\tilde{{\mathcal {F}}}_{s}^{(1)}(\hat{u}_{s})\\ D_1\tilde{{\mathcal {F}}}_{s}^{(2)}(\hat{u}_{s}) &{} D_2\tilde{{\mathcal {F}}}_{s}^{(2)}(\hat{u}_{s}) \end{array}\right) . \end{aligned}$$

Therefore, to compute $Z_0$ it suffices to find an upper bound, uniformly in *s*, for the norm of the above expression as a linear map from $\Omega ^M\rightarrow \Omega ^M$. Interpreted as matrices, $P_s$, $Q_s$, $R_s$ and $S_s$ are interpolants of other explicit matrices. However, the derivatives $D_i\tilde{{\mathcal {F}}}_s^{(j)}(\hat{u}_s)$, while evaluated at interpolants, are themselves “nonlinear” in *s*.

##### Remark 10

The implementation of $||(I-A_sA_s^\dagger )|_{\Omega ^M}||$ is influenced by the way in which the dependence on *s* is handled. For example, *s* can be treated as a vector interval and the norm can be computed “in one step” using interval arithmetic, then we take the interval supremum to get a bound. This can result in some wrapping (over-estimation). One way to control the wrapping is to cover $\Delta$ in a mesh of balls, compute the norm $||(I-A_sA_s^\dagger )|_{\Omega ^M}||$ for *s* replaced with each of these interval balls, and take the maximum. Still another way is to carefully compute a Taylor expansion with respect to *s*, although this task has a few technical issues due to the fact that ${\mathcal {F}}_s(\cdot )$ is generally only $C^1$. We therefore only consider the “in one step” approach in our implementation.

### A Detour: Derivatives of Convolution-Type Delay Polynomials, and Implications to the Regularity of ${\mathcal {F}}_s$

In our implementation of the $Z_1$ and $Z_2$ bounds, we will need to represent various partial derivatives of the map $(z,\rho )\mapsto \textbf{f}(\zeta (\psi )z,\rho )$, where $\rho = (x,a,\psi ,\eta ,\alpha ,\beta )$. This can quickly become notationally cumbersome. Therefore, in this section we will elaborate on the structure of the derivatives of convolution terms of the following map:

29 $$\begin{aligned} \Theta (z,\rho )\overset{\textrm{def}}{=}\rho ^{\textbf{m}}\prod _{p=1}^d \left( e^{i\psi (\rho )\mu _{j_p}K}z_{c_p}\right) \end{aligned}$$

for $z\in \ell _\nu ^1({\mathbb {C}}^{n+m})$ and $\rho \in {\mathbb {R}}^{m+n+4}$. Here, $\psi (\rho )$ denotes the frequency component of $\rho$. In this section, *z* will be indexed with the convention $z=(z_1,\dots ,z_{n+m})$, where $z_q\in \ell _\nu ^1({\mathbb {C}})$ for $q=1,\dots ,n+m$. The objects (29) can be interpreted as individual terms of $\textbf{f}_1$ through $\textbf{f}_{n+m}$. The product symbol indicates iterated convolution, while we remind the reader that $(e^{i\psi \mu K}z)_k = e^{i\psi \mu k}z_k$. Here, $d\in {\mathbb {N}}$ is the (polynomial power) order of the term, the indices $c_p\in \{1,\dots ,n+m\}$ specify which factors of $\ell _\nu ^1({\mathbb {C}}^{n+m})$ are involved in the multiplication, while $j_p\in \{1,\dots ,J\}$ indicates which delays are associated to each of them. Finally, there is a multi-index $\textbf{m}$ for multi-index power $\rho ^\textbf{m}$. Importantly, the multi-index $\textbf{m}$ is trivial in the frequency ($\psi$) component, and the latter only enters $\textbf{f}$ in the form of the delay mapping $\zeta (\psi )$.

#### On the Codomain of $\Theta$

The range of $\Theta$ is $\ell _\nu ^1({\mathbb {C}})$, but we will take an alternate codomain, explicitly defining $\Theta :\ell _\nu ^1({\mathbb {C}}^{n+m})\times {\mathbb {R}}^{n+m+4}\rightarrow K_\nu ({\mathbb {C}})$. This is a very deliberate choice, which we make for the following reasons. First, recall that ${\mathcal {F}}_s$ has range in $\tilde{\Omega }$, which has (in the Fourier components) factors of $K_\nu ({\mathbb {C}})$ due to the presence of the differentiation operator. Therefore, in the scope of the $Z_1$ and $Z_2$ bounds, it is correct to expand the codomain of $\Theta$ to the larger space $K_\nu ({\mathbb {C}})$. Second, if the codomain of $\Theta$ is instead taken to be $\ell _\nu ^1({\mathbb {C}})$, then $\Theta$ would not be differentiable. Indeed, as a simple example, consider the nonlinear map $g(z,\rho )=e^{i\psi (\rho )K}z$ with $g:\ell _\nu ^1({\mathbb {C}})\times {\mathbb {R}}\rightarrow \ell _\nu ^1({\mathbb {C}})$ and $\psi (\rho )=\rho$. This function is an instance of the class $\Theta$. If *g* were differentiable, then necessarily we would have

$$\begin{aligned} DG(z,\rho )h = e^{i\psi (\rho )K}h_1 + Ke^{i\psi (\rho )K}zh_2 \end{aligned}$$

for $h=(h_1,h_2)\in \ell _\nu ^1({\mathbb {C}})\times {\mathbb {R}}$. However, $DG(z,h):\ell _\nu ^1({\mathbb {C}})\times {\mathbb {R}}\rightarrow \ell _\nu ^1({\mathbb {C}})$ is generally unbounded, since one can select $h=(0,1)$ and construct *z* such that $||Kz||_\nu =\infty$ (note that $||Kz||_\nu = ||Ke^{i\psi (\rho )K}z||_\nu$).

#### On the Regularity of $\Theta$

Before proceeding to the main result on differentiability of $\Theta$, we need one preliminary result about the existence of the convolution product $z*Kz$ for $z\in \ell _\nu ^1({\mathbb {C}})$. While the result is elementary (it is closely related to the Leibniz law) and simple to prove, we were unable to find a complete rigorous proof in the literature, so we provide one here.

##### Lemma 6

If $z\in \ell _\nu ^1({\mathbb {C}})$, then $z*Kz\in K_\nu ({\mathbb {C}})$ with $||z*Kz||_{\nu ,K}\le \frac{1}{2}||z||_\nu ^2$.

##### Proof

For each $k\in {\mathbb {Z}}$, $(z*Kz)_k$ exists. This is a consequence of *Kz* being summable. Indeed, assume without loss of generality that $||z||_\nu =1$. Then $|z_j|\le \nu ^{-|j|}\le 1$ for each *j*, and

$$\begin{aligned} (z*Kz)_k=\sum _{j=-\infty }^\infty jz_jz_{k-j}, \end{aligned}$$

which converges uniformly since $|jz_jz_{k-j}|\le |j|\nu ^{-|j|}$ and $\nu >1$. It is then straightforward to verify using the definition of the convolution that $(z*Kz)_k = k(z*z) - (z*Kz)_k$, from which it follows that $(z*Kz) = \frac{k}{2}(z*z)$, and therefore $||(z*Kz)||_{\nu ,K} \le \frac{1}{2}||z*z||_\nu \le \frac{1}{2}||z||_\nu ^2<\infty$. $\square$

The following lemma can now be proven by means of a long, tedious bookkeeping exercise, which we omit.

##### Lemma 7

If $q\in \{1,\dots ,n+m\}$, then for $z\in \ell _\nu ^1({\mathbb {C}}^{n+m})$ and $h\in \ell _\nu ^1({\mathbb {C}})$,

$$\begin{aligned} \frac{d}{dz_q}\Theta (z,\rho )h&=\rho ^\textbf{m}\sum _{\begin{array}{c} r=1 \\ c_r=q \end{array}}^d (e^{i\psi (\rho )\mu _{j_r}K}h)*\prod _{\begin{array}{c} p=1 \\ p\ne r \end{array}}^d e^{i\psi (\rho )\mu _{j_p}K}z_{c_p}\\ \frac{d}{d\psi (\rho )}\Theta (z,\rho )&=\rho ^\textbf{m}\sum _{r=1}^d \left( \prod _{\begin{array}{c} p=1 \\ p\ne r \end{array}}^d e^{i\psi (\rho ) \mu _{j_p}K}z_{c_p}\right) *\left( iK\mu _{j_r}e^{i\psi (\rho ) \mu _{j_r}K}z_{c_r}\right) \\ \frac{d}{dz_q}\left[ \frac{d}{d\psi (\rho )}\Theta (z,\rho )\right] h&=\rho ^\textbf{m}\sum _{\begin{array}{c} r=1 \\ c_r=q \end{array}}^d(iK\mu _{j_r} e^{i\psi (\rho )\mu _{j_r}K}h)*\prod _{\begin{array}{c} p=1 \\ p\ne r \end{array}}^d e^{i\psi (\rho )\mu _{j_p}K}z_{c_p}\\&\quad +\rho ^\textbf{m}\sum _{\begin{array}{c} r=1 \\ c_r=q \end{array}}^d (e^{i\psi (\rho )\mu _{j_r}K}h)*\sum _{\begin{array}{c} p=1 \\ p\ne r \end{array}}^d \left( \prod _{\begin{array}{c} \xi =1 \\ \xi \ne r,p \end{array}}^de^{i\psi (\rho ) \mu _{j_\xi }K}z_{c_\xi }\right) \\ {}&\quad * (iK\mu _{j_p}e^{i\psi (\rho ) \mu _{j_p}K}z_{c_p}) \end{aligned}$$

Also, $\frac{d}{d\psi (\rho )}\left[ \frac{d}{dz_q}\Theta (z,p)h\right] = \frac{d}{dz_q}\left[ \frac{d}{d\psi (\rho )}\Theta (z,\rho )\right] h$. If $z\in {\mathbb {C}}_{\mathbb {Z}}^{n+m}$ is band-limited, $\frac{d^2}{d\psi (\rho )^2}\Theta (z,\rho )$ exists and

$$\begin{aligned} \frac{d^2}{d\psi (\rho )^2}\Theta (z,\rho )&=\rho ^\textbf{m}\sum _{r=1}^d \left( \sum _{\begin{array}{c} p=1\\ p\ne r \end{array}}^d \left( \prod _{\begin{array}{c} q=1 \\ q\ne p,r \end{array}}^d e^{i\psi (\rho )\mu _{j_q}K}z_{c_q} \right) * (iK\mu _{j_p}e^{i\psi (\rho )\mu _{j_p}K}z_{c_p})\right. \\&\quad \left. *(iK\mu _{j_r}e^{i\psi (\rho )\mu _{j_r}K}z_{c_r})\right) \\&\quad +\rho ^\textbf{m}\sum _{r=1}^d\left( \prod _{\begin{array}{c} p=1 \\ p\ne r \end{array}}^d e^{i\psi (\rho )\mu _{j_p}K}z_{c_p}\right) *\left( -K^2\mu _{j_r}^2 e^{i\psi (\rho )\mu _{j_r}K}z_{c_r}\right) . \end{aligned}$$

Finally, if $q_1,q_2\in \{1,\dots ,n+m\}$ and $h_1,h_2\in \ell _\nu ^1({\mathbb {C}})$, then

$$\begin{aligned}&\frac{d^2}{dz_{q_2}dz_{q_1}}\Theta (z,p)[h_1,h_2]\\&\quad =\rho ^\textbf{m}\sum _{\begin{array}{c} r=1 \\ c_r=q_1 \end{array}}^d (e^{i\psi (\rho )\mu _{j_r}K}h_1)* \left( \sum _{\begin{array}{c} p=1 \\ c_p = q_2 \\ p\ne r \end{array}}^d (e^{i\psi (\rho )\mu _{j_p}K}h_2)* \prod _{\begin{array}{c} \xi =1 \\ \xi \ne r,p \end{array}}^d e^{i\psi (\rho )\mu _{j_\xi }K}z_{c_\xi }\right) \end{aligned}$$

##### Remark 11

The requirement that *z* be band-limited really is necessary for the existence of $\frac{d^2}{d\psi (\rho )^2}\Theta (z,p)$. Indeed, if $z\in \ell _\nu ^1({\mathbb {C}}^{n+m})$, then $Kz_j\in K_\nu ({\mathbb {C}})$ but $K^2 z$ is not, and the latter terms appear in the second derivative.

#### On the Regularity of ${\mathcal {F}}_s$ and invertibility of $D{\mathcal {F}}_s$

Before moving on to the $Z_1$ and $Z_2$ bound calculations, let us remind ourselves of the following corollary to Theorem 1, which we state somewhat informally: if the hypotheses of theorem are satisfied for the map $F_s:X\rightarrow Y$ (whose zeroes, parameterized by *s*, we wish to prove), then $DF_s(x_s)$ is boundedly invertible. This can be proven by means of a Neumann series argument; see later the proof of Proposition 12 where this is done. In other words, if the computer-assisted proof is successful, then $DF_s(x_s)$ must be boundedly invertible.

Now, recall that our map of interest, ${\mathcal {F}}_s:\Omega \rightarrow \tilde{\Omega }$, has domain $\Omega$ whose “Fourier series sequences” are in $\ell _\nu ^1({\mathbb {C}})$, and codomain $\tilde{\Omega }$ with “Fourier series sequences” in $K_\nu ({\mathbb {C}})$. Importantly, the range of ${\mathcal {F}}_s$ is *not* in $\Omega$, because ${\mathcal {F}}_s$ contains a differentiation operator. By Lemma 7 and the subsequent remark, we know that ${\mathcal {F}}_s$ is $C^1$, but is *not* twice differentiable. As will be demonstrated in subsequent sections, this formulation of the map ${\mathcal {F}}_s$ results in successful computer-assisted proofs, which implies that $D{\mathcal {F}}_s(x_s):\Omega \rightarrow \tilde{\Omega }$ is boundedly invertible.

We might now ask what happens if we embed the codomain of ${\mathcal {F}}_s$ into a space of “lower regularity”. For example, consider an embedding $j_p:\tilde{\Omega }\hookrightarrow \Omega _p$, where

$$\begin{aligned} \Omega _p = \pi ^M\text{ Symm }(K_\nu ^p({\mathbb {C}}^{n+m}))\times {\mathbb {R}}^{n+m+4} \times \pi ^\infty \text{ Symm }(K_\nu ^p(C^{n+m})), \end{aligned}$$

and the space $K_\nu ^p({\mathbb {C}}^{n+m})$ is the subspace of ${\mathbb {C}}^{\mathbb {Z}}_{n+m}$ whose elements *z* satisfy $||z||_{\nu ,p}\overset{\textrm{def}}{=}|z_0| + \sum _{|k|>0}(\nu ^{|k|}/|k|^p)|z_k|<\infty$. Note that $\Omega =\Omega _0$, $\tilde{\Omega } = \Omega _1$, the strict inclusion $\Omega _p\subset \Omega _{p+1}$ holds, and at the level of the Fourier space factors, $K_\nu ^p$ can be interpreted as the “space of *p*th derivatives” of $\ell _\nu ^1$. It is possible to prove the following.

##### Lemma 8

$j_p\circ {\mathcal {F}}_s$ is $C^p$, for any $p\ge 1$.

However, if $p>1$, previous remarks demonstrate that $Dj_p\circ {\mathcal {F}}_s(x_s):\Omega _0\rightarrow \Omega _p$ will generally *not* be boundedly invertible. In other words, at the level of the computer-assisted proof, nothing can be gained by embedding the codomain of ${\mathcal {F}}_s$ in a space of lower regularity.

### The Bound $Z_1$

To have a hope at deriving a $Z_1$ bound, we will first determine the structure of the operator $D{\mathcal {F}}_s(\hat{u}_s) - A_s^\dagger$. Represented as an “infinite block matrix”, most blocks are zero. One can verify that

30 $$\begin{aligned} D{\mathcal {F}}_s(\hat{u}_s)-A_s^\dagger&=\left( \begin{array}{ccc} 0&{}0&{}\textbf{Z}_1^{(1,3)} \\ 0&{}0&{}0\\ \textbf{Z}_1^{(3,1)}&{}\textbf{Z}_1^{(3,2)}&{}\textbf{Z}_1^{(3,3)} \end{array}\right) , \end{aligned}$$

with the individual terms $\textbf{Z}_1^{(i,j)}$ being the operators

$$\begin{aligned} \textbf{Z}_1^{(1,3)}&=\pi ^MD_1\textbf{f}(\zeta (\hat{\psi }_s)\hat{z}_s, \hat{\rho }_s)\zeta (\hat{\psi }_s)\pi ^\infty \\ \textbf{Z}_1^{(3,1)}&=\pi ^\infty D_1\textbf{f}(\zeta (\hat{\psi }_s) \hat{z}_s,\hat{\rho }_s)\zeta (\hat{\psi }_s)\pi ^M\\ \textbf{Z}_1^{(3,2)}&=\pi ^\infty D_1\textbf{f}(\zeta (\hat{\psi }_s) \hat{z}_s,\hat{\rho }_s)\zeta '(\hat{\psi }_s)\hat{z}_s\psi (\cdot ) + \pi ^\infty D_2\textbf{f}(\zeta (\hat{\psi }_s)\hat{z}_s,\hat{\rho }_s)\\ \textbf{Z}_1^{(3,3)}&=\pi ^\infty D_1\textbf{f}(\zeta (\hat{\psi }_s) \hat{z}_s,\hat{\rho }_s)\zeta (\hat{\psi }_s)\pi ^\infty , \end{aligned}$$

and $\psi :{\mathbb {R}}^{n+m+4}\rightarrow {\mathbb {R}}$ once again denoting the frequency component projection. Computing $Z_1$ requires precomposing (30) with $A_s$, which has the structure (26).

The development of general-purpose, implementable $Z_1$ bounds (and, subsequently, the $Z_2$ bounds) will be made easier if we introduce *interval unit vectors*. In one of the spaces ${\mathbb {R}}^k$ or ${\mathbb {C}}^k$ (for some dimension *k*), the interval unit vector is the unique interval vector *V* with the property that $||V||=1$, and for any other interval vector *v* with $||v||=1$, the inclusion $v\subseteq V$ is satisfied. We remind the reader that the norm on ${\mathbb {R}}^k$ or ${\mathbb {C}}^k$ is always taken to be a weighted supremum norm, so the unit interval vector will depend on the weights.

#### Virtual Padding

For $h\in \Omega$ with $||h||_\Omega \le 1$, denote

31 $$\begin{aligned} g = A_s(D{\mathcal {F}}_s(\hat{u}_s) - A_s^\dagger )h, \ \ g=(g^M,g^\rho ,g^\infty ). \end{aligned}$$

Computing $Z_1$ is equivalent to a uniform (in *h*) bound for $||g||_\Omega$. The tightness of this bound is determined by two levels of computation:

- Some finite norm computations that are done on the computer;
- Theoretical bounds, which are inversely proportional to the dimension of the object on which the finite norm computations are done.

By default, the size of the finite computation is linear in *M*, the number of modes. This might suggest that explicitly increasing *M* – that is, padding our solution with extra zeros and re-computing everything with more modes – is the only way to improve the bounds. Thankfully, this is not the case.

**Fig. 4:**
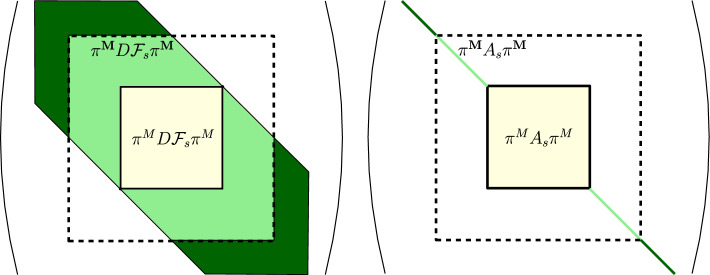
Visual depiction of the virtual padding process as it applies to the operators $D{\mathcal {F}}_s$ and $A_s$, left and right. When we do not do virtual padding ($\textbf{M}=M$), the size of the objects we store in memory on the computer for matrix operations are strictly determined by the dimension of the numerical zero. These objects are the inner boxes in the infinite matrices above, and are depicted in pale yellow. The part outside of this box is controlled analytically. When we use nontrivial virtual padding $(\textbf{M}>M$), we store a larger amount of information in the computer for matrix operations; this is the outer dashed line box. Once again, the region outside the box is controlled analytically. Regions in white represent zeroes, while dark green represents the infinite part of the matrix, and light green a finite part of the matrix that is analytically controlled if there is no virtual padding (Color figure online)

Intuitively, $A_s(D{\mathcal {F}}_s(\hat{u}_s-A_s^\dagger )$ is an “infinite matrix”, for which we have a canonical numerical center determined by the pre- and post- composition with the projection operator onto $\pi ^M{{\,\textrm{Symm}\,}}(\ell _\nu ^1({\mathbb {C}}^{n+m}))\times {\mathbb {R}}^{n+m+4}$. This is determined by the number of modes *M* we have specified in our numerical zero. However, there is no reason to only compute this much of the infinite matrix explicitly; we could instead choose $\textbf{M}\ge M$ and compute the representation of this operator on $\pi ^\textbf{M}{{\,\textrm{Symm}\,}}(\ell _\nu ^1({\mathbb {C}}^{n+m}))\times {\mathbb {R}}^{n+m+4}$. The result is that a larger portion of $A_s(D{\mathcal {F}}_s(\hat{u}_s-A_s^\dagger )$ is stored in the computer’s memory. The advantage of doing this is that the explicit computations of norms are generally much tighter than theoretically guaranteed estimates, while the theoretical bounds related to the tail will be proportional to $\frac{1}{\textbf{M}+1}$. See Fig. 4 for a visual representation.

To exploit these observations, we decompose $\ell _\nu ^1({\mathbb {C}}^{n+m})$ (and hence the symmetric subspace) as a direct sum

$$\begin{aligned} \pi ^\textbf{M}(\ell _\nu ^1({\mathbb {C}}^{n+m}))\oplus \pi ^{\textbf{M}+1,\infty } (\ell _\nu ^1({\mathbb {C}}^{n+m})), \end{aligned}$$

where $\textbf{M}\ge M$, and $\pi ^{\textbf{M}+1,\infty }$ is now interpreted as the complementary projector to $\pi ^\textbf{M}$. In the subsections that follow, $\textbf{M}$ will be the *virtual padding dimension*. We therefore re-interpret $h=(h^\textbf{M},h^\rho ,h^\infty )$ and $g=(g^\textbf{M},g^\rho ,g^\infty )$ as being in the product space

$$\begin{aligned} \pi ^{\textbf{M}}{{\,\textrm{Symm}\,}}(\ell _\nu ^1({\mathbb {C}}^{n+m}))\times {\mathbb {R}}^{n+m+4} \times \pi ^{\textbf{M}+1,\infty }{{\,\textrm{Symm}\,}}(\ell _\nu ^1({\mathbb {C}}^{n+m})), \end{aligned}$$

which remains (isometrically) isomorphic to $\Omega$. Remark that the finite blocks $P_s,Q_s$ and $R_s$ coming from $A_s$ must now be re-interpreted as linear maps involving $\pi ^\textbf{M}{{\,\textrm{Symm}\,}}(\ell _\nu ^1({\mathbb {C}}^{n+m}))$, as appropriate. Similarly, the $\textbf{B}$ blocks can be replaced by

$$\begin{aligned} \textbf{Z}_1^{(1,3)}&=\pi ^\textbf{M}D_1\textbf{f}(\zeta (\hat{\psi }_s) \hat{z}_s,\hat{\rho }_s)\zeta (\hat{\psi }_s)\pi ^{\textbf{M}+1,\infty }\\ \textbf{Z}_1^{(3,1)}&=\pi ^{\textbf{M}+1,\infty } D_1\textbf{f}(\zeta (\hat{\psi }_s)\hat{z}_s,\hat{\rho }_s)\zeta (\hat{\psi }_s)\pi ^\textbf{M}\\ \textbf{Z}_1^{(3,2)}&=\pi ^{\textbf{M}+1,\infty } D_1\textbf{f}(\zeta (\hat{\psi }_s)\hat{z}_s,\hat{\rho }_s)\zeta '(\hat{\psi }_s) \hat{z}_s\psi (\cdot ) + \pi ^{\textbf{M}+1,\infty } D_2\textbf{f} (\zeta (\hat{\psi }_s)\hat{z}_s,\hat{\rho }_s)\\ \textbf{Z}_1^{(3,3)}&=\pi ^{\textbf{M}+1,\infty } D_1\textbf{f}(\zeta (\hat{\psi }_s)\hat{z}_s,\hat{\rho }_s)\zeta (\hat{\psi }_s) \pi ^{\textbf{M}+1,\infty }, \end{aligned}$$

In our implementation, this virtual padding is implemented automatically on an as-needed basis.

Related to this virtual padding are the block projection operators $\pi ^{j_1,j_2}:\ell _\nu ^1({\mathbb {C}}^{n+m})\rightarrow \ell _\nu ^1({\mathbb {C}}^{n+m})$ defined according to

$$\begin{aligned} (\pi ^{j_1,j_2}z)_k = \left\{ \begin{array}{ll} z_k,&{}j_1\le |k| \le j_2 \\ 0 &{} \text{ otherwise. } \end{array}\right. \end{aligned}$$

These will be needed in subsequent sections, since the padding dimension and the degree of the polynomial nonlinearities will play a role in the amount of data that must be stored to capture the $Z_1$ and $Z_2$ bounds. We define a related interval sequence $\textbf{1}_{j_1,j_2}\subset \ell _\nu ^1({\mathbb {C}}^{n+m})$ as

$$\begin{aligned} (\textbf{1}_{j_1,j_2})_k&=\left\{ \begin{array}{ll} {[}\textbf{1}_{\mathbb {C}}]^{n+m}\nu ^{-|k|},&{}j_1\le |k|\le j_2 \\ 0 &{} \text{ otherwise }, \end{array}\right. \end{aligned}$$

where $[\textbf{1}_{\mathbb {C}}]^{n+m}\subset {\mathbb {C}}^{n+m}$ is the unit interval vector in ${\mathbb {C}}^{n+m}$. This sequence generates an *implementable* enclosure of the image of the closed unit ball in $\ell _\nu ^1({\mathbb {C}}^{n+m})$ under $\pi ^{j_1,j_2}$.

#### The Norm $||g^\textbf{M}+g^\infty ||$

Recall that the quantity *g* that we must bound to obtain $Z_1$ is decomposed into three parts: $g=(g^\textbf{M},g^\rho ,g^\infty )$; see (31) and the subsequent discussion. Here we compute the bound of the Fourier part.

Before we begin deriving the bound, note that the sum $g^\textbf{M}+g^\infty$ can be written

32 $$\begin{aligned} g^\textbf{M}+g^\infty&=P_s\textbf{Z}_1^{1,3}h^\infty + i\hat{\psi }_s^{-1} (I - Q_s D_3\tilde{{\mathcal {F}}}_s^{(2)}(\hat{u}_s))(K\pi ^{\textbf{M}+1:\infty })^{-1}\nonumber \\&\quad (\textbf{Z}_1^{3,1}h^\textbf{M}+ \textbf{Z}_1^{3,2}h^\rho + \textbf{Z}_1^{3,3}h^\infty ) \nonumber \\&=P_s\textbf{Z}_1^{1,3}h^\infty + i\hat{\psi }_s^{-1}(I - Q_s T (\hat{u}_s,\cdot ))(K\pi ^{\textbf{M}+1:\infty })^{-1}\nonumber \\ {}&\quad (\textbf{Z}_1^{3,1} h^\textbf{M}+ \textbf{Z}_1^{3,2}h^\rho + \textbf{Z}_1^{3,3}h^\infty ) \end{aligned}$$

where *T* is defined as

$$\begin{aligned} T(u,w)&= \left( \begin{array}{c} 0_{{\mathbb {R}}^m} \\ 0_{\mathbb {R}}\\ 0_{\mathbb {R}}\\ D_1\theta _{BC}\left( \sum _k z_k,x,a,\alpha ,\beta ,\eta \right) \sum _j w_j \\ 0_{\mathbb {R}}\\ 0_{\mathbb {R}}\end{array} \right) , \end{aligned}$$

and $$u=(z,(x,a,\alpha ,\beta ,\eta ))\in \ell _\nu ^1 ({\mathbb {C}}^{n+m})\times {\mathbb {R}}^{n+m+4}$$.

##### Lemma 9

Let $z\mapsto \textbf{f}(z,\cdot )$ be a convolution polynomial of degree *q*. Let^3^$\psi _\textbf{1} = [-1,1]\cdot ||(0,(0,0,1,0,0))||_{\Omega ^M}$. Then

$$\begin{aligned} ||g^\textbf{M}+ g^\infty ||&\le ||P_s\pi ^\textbf{M}D_1 \textbf{f}(\zeta (\hat{\psi }_s)\hat{z}_s,\hat{\rho }_s) \textbf{1}_{\textbf{M}+1,q\textbf{M}}||\\ {}&\quad + \frac{1}{\textbf{M}+1}|\hat{\psi }_s|^{-1}||I -Q_sT(\hat{u}_s,\textbf{v}_s + \textbf{w}_s)||, \end{aligned}$$

where the finitely-supported interval sequences $\textbf{v}_s,\textbf{w}_s\subset \ell _\nu ^1({\mathbb {C}}^{n+m})$ are defined according to

$$\begin{aligned} \textbf{w}_s&=\pi ^{\textbf{M}+1,qM}\Big (D_1\textbf{f} (\zeta (\hat{\psi }_s)\hat{z}_s,\hat{\rho }_s)\zeta ' (\hat{\psi }_s)\hat{z}_s\psi _\textbf{1}^{-1} + D_2 \textbf{f}(\zeta (\hat{\psi }_s)\hat{z}_s, \hat{\rho }_s)[\textbf{1}_{\mathbb {R}}]^{n+m+4}\Big )\\ (\textbf{v}_s)_{j,k}&= \left\{ \begin{array}{ll} ([\textbf{1}_{\mathbb {C}}]^{n+m})_j||D_1\textbf{f}_j (\zeta (\hat{\psi }_s)\hat{z}_s,\hat{\rho }_s)||_{L(\ell _\nu ^1({\mathbb {C}}^{n+m}), \ell _\nu ^1({\mathbb {C}}))},&{} j=1,\dots ,m+n, k=0\\ 0 &{} j=1,\dots ,m+n, k\ne 0 \end{array}\right. \end{aligned}$$

##### Proof

Recall the decomposition (32). Since $z\mapsto \textbf{f}(z,\cdot )$ is a convolution polynomial of degree *q*, we have $(D_1\textbf{f}(\zeta (\hat{\psi }_s)\hat{z}_s,\hat{\rho }_s) e_k)_j = 0$ for all $|j|\le \textbf{M}$ whenever $|k|> q\textbf{M}$. It follows that the support of $w\mapsto \pi ^\textbf{M}D_1\textbf{f}(\zeta (\hat{\psi }_s)\hat{z}_s,\hat{\rho }_s)w$ is contained in the range of $\pi ^{0,q\textbf{M}}$. Restricting to the range of $\pi ^{\textbf{M}+1,\infty }$,

$$\begin{aligned} ||P_s\textbf{Z}_1^{1,3}h^\infty ||\le \left| \left| P_s\pi ^\textbf{M}D_1\textbf{f}(\zeta (\hat{\psi }_s)\hat{z}_s,\hat{\rho }_s) \textbf{1}_{\textbf{M}+1,q\textbf{M}}\right| \right| . \end{aligned}$$

Next, the range of $h^\rho \mapsto \textbf{Z}_1^{(3,2)}h^\rho$ is contained in that of $\pi ^{0,qM}$ (recall that while we have used a different padding dimension $\textbf{M}$ for the computation of norms, our data $\hat{z}_s$ is still band-limited to *M* modes). We have

$$\begin{aligned}&||\textbf{Z}_1^{3,1}h^\textbf{M}+ \textbf{Z}_1^{3,3}h^\infty ||=||D_1\textbf{f} (\zeta (\hat{\psi }_s)\hat{z}_s,\hat{\rho }_s)\zeta (\hat{\psi }_s) (h^\textbf{M}+h^\infty )|| \le ||\textbf{v}_s||,\\&\textbf{Z}_1^{3,2}h^\rho =\pi ^{\textbf{M}+1,\infty }\big (D_1\textbf{f} (\zeta (\hat{\psi }_s)\hat{z}_s,\hat{\rho }_s)\zeta '(\hat{\psi }_s) \hat{z}_s\psi (\cdot ) + D_2\textbf{f}(\zeta (\hat{\psi }_s) \hat{z}_s,\hat{\rho }_s)\big )h^\rho \in \textbf{w}_s \end{aligned}$$

Using these bounds/inclusions, the fact that $\textbf{v}_s$ is supported on the zeroth Fourier index, and the properties of the map *T*, we get

$$\begin{aligned}&||(I - Q_s T(\hat{u}_s,\cdot ))(K\pi ^{\textbf{M}+1:\infty })^{-1}(\textbf{Z}_1^{3,1} h^\textbf{M}+ \textbf{Z}_1^{3,2}h^\rho + \textbf{Z}_1^{3,3}h^\infty )||\\&\quad \le \frac{||I-Q_sT(\hat{u}_s,\textbf{v}_s + \textbf{w}_s)||}{\textbf{M}+1}. \end{aligned}$$

Combining the previous bounds, we get the result claimed in the lemma. $\square$

##### Remark 12

While perhaps symbolically intimidating, all of the quantities appearing in the statement of Lemma 9 are explicitly machine-computable, so a rigorous uniform (in *s* and *h*) bound for $||g^\textbf{M}+ g^\infty ||$ can indeed be computed.

#### The Norm $||g^\rho ||$

In this section, the norm $||\cdot ||$ on ${\mathbb {R}}^{n+m+4}$ is identified with $x\mapsto ||(0,x,0)||_\Omega$. The bound for $||g^\rho ||$ bears a lot of similarity to the one for $||g^\textbf{M}+ g^\infty ||$, and its derivation is similar. We omit the proof of the following lemma.

##### Lemma 10

With the same notation as in Lemma 9, we have the bound

$$\begin{aligned} ||g^\rho || \le ||R_s\pi ^\textbf{M}D_1\textbf{f}(\zeta (\hat{\psi }_s) \hat{z}_s,\hat{\rho }_s)\textbf{1}_{\textbf{M}+1,q\textbf{M}}|| + \frac{1}{\textbf{M}+1}|\hat{\psi }_s|^{-1}||S_sT(\hat{u}_s, \textbf{v}_s + \textbf{w}_s)||. \end{aligned}$$

#### Summary of the $Z_1$ Bound

Combining the results of Lemma 9 and Lemma 10, it follows that if we choose $Z_1$ such that

$$\begin{aligned} Z_1&\ge \max \left\{ ||P_s\pi ^\textbf{M}D_1\textbf{f} (\zeta (\hat{\psi }_s)\hat{z}_s,\hat{\rho }_s)\textbf{1}_{\textbf{M}+1,q\textbf{M}}|| +\frac{1}{\textbf{M}+1}|\hat{\psi }_s|^{-1}||I-Q_sT(\hat{u}_s,\textbf{v}_s +\textbf{w}_s)||\ , \right. \\&\qquad \quad \left. ||R_s\pi ^\textbf{M}D_1\textbf{f} (\zeta (\hat{\psi }_s)\hat{z}_s,\hat{\rho }_s)\textbf{1}_{\textbf{M}+1,q\textbf{M}}|| +\frac{1}{\textbf{M}+1}|\hat{\psi }_s|^{-1}||S_sT(\hat{u}_s,\textbf{v}_s +\textbf{w}_s)|| \right\} \end{aligned}$$

for all $s\in \Delta$, then $Z_1$ will satisfy the bound (11) for our validated continuation problem. In the above bound, one must remember that the norms $||\cdot ||$ are actually restrictions of the norm $||\cdot ||_\Omega$ to various subspaces; see the preamble before Lemma 9 and Lemma 10.

### The Bound $Z_2$

It is beneficial to perform a further splitting of the expression that defines the $Z_2$ bound. Recall that $Z_2(r)$ should satisfy

33 $$\begin{aligned} ||A_s(D{\mathcal {F}}_s(\hat{u}_s+\delta ) - D{\mathcal {F}}_s (\hat{u}_s))||_{B(\Omega ,\Omega )}\le Z_2(r) \end{aligned}$$

uniformly for $s\in \Delta$ and $\delta \in \overline{B_r(\hat{u}_s)}$. From Remark 11, $D{\mathcal {F}}_s(\cdot )$ is not itself differentiable, so appealing to a “typical” second derivative estimate for this $Z_2$ bound is not going to work. To handle this, we use the strategy of [31] and split the norm to be computed using a triangle inequality. Given $\delta \in \overline{B_r(\hat{u}_s)}$, split it as $\delta =\delta _1+\delta _2$, where the components of $\delta _1$ are all zero *except for the frequency component*, $\psi$, and $\delta _2$ has zero for its frequency component. This decomposition is uniquely defined. Then, consider two new bounds to be computed:

$$\begin{aligned} ||A_s(D{\mathcal {F}}_s(\hat{u}_s + \delta _1) - D{\mathcal {F}}_s (\hat{u}_s))||_{B(\Omega ,\Omega )}&\le Z_{2,1}(r)\\ ||A_s(D{\mathcal {F}}_s(\hat{u}_s + \delta _1+\delta _2) -D{\mathcal {F}}_s(\hat{u}_s + \delta _1))||_{B(\Omega ,\Omega )}&\le Z_{2,1}(r) \end{aligned}$$

uniformly for $s\in \Delta$ and $\delta \in \overline{B_r(\hat{u}_s)}$. If we define $Z_2(r)\overset{\textrm{def}}{=}Z_{2,1}(r)+Z_{2,2}(r)$, then (33) will be satisfied. It turns out that this decomposition is effective. We will elaborate on this now.

#### The Bound $Z_{2,1}$

The partial derivative $\frac{d}{d\psi (\rho )} D{\mathcal {F}}_s(\hat{z}^M_s,\hat{\rho }_s,0)$ exists and is continuous. This is a consequence of Lemma 7 (n.b. the inputs are band-limited) and the structure of ${\mathcal {F}}_s$; see (20). As $A_s$ is bounded, we can use the fundamental theorem of calculus (in Banach space) to obtain

34 $$\begin{aligned} ||A_s(D{\mathcal {F}}_s(\hat{u}_s+\delta _1)-D{\mathcal {F}}_s(\hat{u}_s))h||_\Omega \le \sup _{t\in [0,1]}\left| \left| A_s\left( \frac{d}{d\psi (\rho )}D {\mathcal {F}}_s(\hat{u}_s + t\delta _1)h\right) \psi (\delta _1)\right| \right| _\Omega \end{aligned}$$

for any $h\in \Omega$, $||h||_\Omega \le 1$. Due to the structure of ${\mathcal {F}}_s$, the derivative term in the large parentheses will have

- In the $\ell _\nu ^1({\mathbb {C}}^{n+m})$ components: convolution polynomials of the form (29) involving Fourier components coming from the set
  $$\begin{aligned} \{\zeta (\hat{\psi }_s +\psi (\delta _1))\hat{z}_s\}\cup \{i\zeta (\hat{\psi }_s +\psi (\delta _1))K\hat{z}_s\}\cup \{-\zeta (\hat{\psi }_s +\psi (\delta _1))K^2\hat{z}_s\}\cup \{h\} \end{aligned}$$
  with coefficients in $\hat{\rho }_s$;
- In the scalar components: finite-dimensional range functions of $\sum _{|k|\le M}(\hat{z}_s)_k$ and $\psi (\delta _1)$.

To obtain the tightest possible upper bound for (34), one would want to exploit as much structure of $D{\mathcal {F}}_s$ (and the directional derivative in the frequency direction) as possible. However, to get a general-purpose *implementable* bound, we can apply the following operations to $\frac{d}{d\psi (\rho )}D{\mathcal {F}}_s(\hat{u}_s + t\delta _1)h\psi (\delta _1)$.

1. Replace all instances of $\zeta (\hat{\psi }_s + \psi (\delta _1))$ with the interval $[-1,1]$;
2. Replace the component of *h* in ${\mathbb {R}}^{n+m+4}$ with the unit interval vector^4^ in $\times {\mathbb {R}}^{n+m+4}$;
3. Replace all other variables^5^ described in the bulleted list above with bounds for their norms, multiplied by the zero-supported basis element of the relevant space (e.g. for $\ell _\nu ^1$ elements, the identity $e_0$ of the Banach algebra; for $\ell _\nu ^1({\mathbb {C}}^{n+m})$, the vectorized version of $e_0$), multiplied by the interval $[-1,1]$;
4. Compute operator norms of the blocks of $A_s$ in (26);
5. Complete block multiplications, taking the norm of the result, followed by an interval supremum (note, $t\in [0,1]$ is also replaced by an interval).

We emphasize in the algorithm above, every object is a finite-dimensional quantity, so operations can indeed be performed in a suitable complex vector space on the computer. This strategy produces a true bound for (34) largely because of the Banach algebra and the interval arithmetic. For example, consider the impact of this computation on the quantity

$$\begin{aligned} -\hat{z}_s*h + (e^{iK(\hat{\psi }_s + \psi (\delta _1))} \hat{z}_s)*(iKe^{iK(\hat{\psi }_s+\psi (\delta _1)}\hat{z}_s)*h \end{aligned}$$

in $\ell _\nu ^1$. From the Banach algebra, if $||h||_\nu \le 1$, then a triangle inequality produces

$$\begin{aligned} ||\hat{z}_s|| + ||e^{iK(\hat{\psi }_s + \psi (\delta _1)} \hat{z}_s||_\nu \cdot ||iKe^{iK(\hat{\psi }_s + \psi (\delta _1)} \hat{z}_s||_\nu&= ||(ae_0)*(ce_0)||_\nu \\ {}&\quad + ||(ae_0)*(be_0)*(ce_0)||\\&= ac + abc, \end{aligned}$$

where $a = ||\hat{z}_s||_\nu$, $b = ||iK\hat{z}_s||_\nu$, $c=||h||_\nu =1$, and $e_0$ is the identity element in the Banach algebra $\ell _\nu ^1$. Now, define $\textbf{1} = [-1,1]$ and compare the result to

$$\begin{aligned} -(\textbf{1}ae_0)*(\textbf{1}ce_0) + (\textbf{1}a e_0)* (\textbf{1}be_0)*(\textbf{1}ce_0), \end{aligned}$$

which is what our algorithm would produce. The support of this sequence is the zeroth index, and we can explicitly compute the resulting interval. It is precisely $\textbf{1}\cdot (ac + abc)e_0$. The supremum of this interval is $ac + abc$, which matches the analytical triangle inequality / Banach algebra bound computed previously.

##### Remark 13

The bound obtained by applying the strategy above is incredibly crude; in effect, we use the triangle inequality for everything. However, producing fully general code to compute the second derivatives for this class of problems (polynomial delay differential equations with arbitrary numbers of delays) would be a messy programming task. Even with the present implementation, where we need only compute second derivatives evaluated at sequences that are supported on the zeroth Fourier mode, the implementation was far from trivial. Long term, it would be beneficial to implement second derivatives, as this would also allow for improvements to the $Y_0$ bound; see Sect. 4.2. The good news is that, theoretically, the computed bound will be *O*(*r*) for *r* small enough, due to the linear multiplication of $\psi (\delta _1)=O(r)$ appearing in (34).

#### The Bound $Z_{2,2}$

Let $d_{\delta _2}$ denote the Gateaux derivative of $D{\mathcal {F}}_s(\cdot )$ in the direction $\delta _2$. We claim

$$\begin{aligned} d_{\delta _2}D{\mathcal {F}}_s(\hat{u}_s+\delta _1+t\delta _2) \end{aligned}$$

exists and is continuous for $t\in [0,1]$. To see why, observe that that $D{\mathcal {F}}_s^{(2)}$ is continuously differentiable, so we need only worry about the part in $\ell _\nu ^1({\mathbb {C}}^{n+m})$, namely the components $D{\mathcal {F}}_s^{(1)}$ and $D{\mathcal {F}}_s^{(3)}$ that come from the vector field. The result now follows from Lemma 7. Indeed, a band-limited input is only required for the double derivative with respect to the frequency variable, and $\delta _2$ has zero for its frequency component. By the fundamental theorem of calculus,

35 $$\begin{aligned}&||A_s(D{\mathcal {F}}_s(\hat{u}_s+\delta _1+\delta _2)-D{\mathcal {F}}_s(\hat{u}_s +\delta _1))h||_\Omega \nonumber \\ {}&\quad \le \sup _{t\in [0,1]} \left| \left| A_s\left( d_{\delta _2}D{\mathcal {F}}_s(\hat{u}_s + \delta _1 + t\delta _2)h\right) \right| \right| _\Omega \end{aligned}$$

where we take $h\in \Omega$, $||h||_\Omega \le 1$.

To bound (35), we make use of a very similar strategy to the one from Sect. 4.5.1 for $Z_{2,1}$. The difference here is that the convolution polynomials in the $\ell _\nu ^1({\mathbb {C}}^{n+m})$ part of the Gateaux derivative have a more difficult structure. The main problem is in the mixed derivatives with respect to frequency and Fourier space; see the derivatives $\frac{d}{dz_q}\frac{d}{d\psi (\rho )}$ in Lemma 7. These derivatives involve the action of the derivative operator *K* on the sequence part of *h*, which is not necessarily band-limited. If $h=(h_z,h_\rho )$ for $h_z\in \ell _\nu ^1$, and this sequence is not band-limited, then generally $Kh_z$ will not have a finite $\ell _\nu ^1$-norm. To combat this problem, we remark that *only one such term can appear in any given convolution polynomial*. This can be exploited as follows.

To begin, we factor $A_s$ as follows.

$$\begin{aligned} A_s = \left( \begin{array}{ccc} (M+1) P_s &{} Q_s &{} V_s^{(1)} \\ (M+1) R_s &{} S_s &{} V_s^{(2)} \\ 0&{}0&{}V_s^{(3)} \end{array}\right) \left( \begin{array}{ccc} I\frac{1}{M+1}&{}0&{}0\\ 0&{}I&{}0\\ 0&{}0&{}(K\pi ^\infty )^{-1} \end{array}\right) \overset{\textrm{def}}{=}\textbf{A}_s \textbf{K}^{-1}. \end{aligned}$$

Note that $\textbf{A}_s$ is obtained from $A_s$ by multiplying (on the right) the first “column” by $(M+1)$, and the last “column” by $K\pi ^\infty$. The following lemma is a specific case of a lemma of van den Berg, Groothedde and Lessard [31].

##### Lemma 11

Define an operator $\tilde{K}^{-1} = \frac{1}{M+1}\pi ^M +K^{-1}\pi ^\infty$ on $\ell _\nu ^1$. Let $u,v,\in \ell _\nu ^1$. Then

$$\begin{aligned} ||\tilde{K}^{-1}(Ku*v)||_\nu&\le C_\nu ||u||_\nu ||v||_\nu , \qquad C_\nu = \left\{ \begin{array}{ll} \frac{\nu ^{2M+2}}{e\log \nu ^{2M+2}},&{}\nu ^{2M+2}<e \\ 1,&{}\nu ^{2M+2}\ge e. \end{array}\right. \end{aligned}$$

Now consider the product $\textbf{K}^{-1}d_{\delta _2}D{\mathcal {F}}_s(\hat{u}_s + \delta _1 + t\delta _2)h$. The part in $\ell _\nu ^1({\mathbb {C}}^{n+m})$ of the Gateaux derivative $d_{\delta _2}D{\mathcal {F}}_s(\hat{u}_s + \delta _1 + t\delta _2)h$ will be multiplied by the diagonal operator $(\tilde{K}^{-1},\dots ,\tilde{K}^{-1})$. So consider how we might bound a term of the form

36 $$\begin{aligned} \tilde{K}^{-1}\left( \alpha iKh_z*\prod _{j} e^{iK\psi (\hat{\rho }_s + \psi (\delta _1))\mu _j} (\hat{z}_s + tz(\delta _2))_j \right) \in \ell _\nu ^1({\mathbb {C}}) \end{aligned}$$

in a given convolution polynomial that contains a factor $Kh_z$, for $h_z\in \ell _\nu ^1$ and $||h_z||_\nu \le 1$. Here, $\alpha$ is a scalar that could depend on $\hat{\rho }_s$, the scalar components of $t\delta _2$, and the delays, while $z(\delta _2)$ is the part of $\delta _2$ in $\ell _\nu ^1({\mathbb {C}}^{n+m})$. Note that by permutation invariance of the convolution, we may assume that $Kh_z$ appears as the first term on the left, as we have done here. By Lemma 9, the above is bounded above by

$$\begin{aligned} C_\nu \left| \left| \alpha \prod _{j}e^{iK\psi (\hat{\rho }_s +\psi (\delta _1))\mu _j}(\hat{z}_s + tz(\delta _2))_j\right| \right| _\nu . \end{aligned}$$

We can achieve the same effect by replacing $Kh_z$ in (36) with $C_\nu e_0$, for $e_0$ being the identity in the convolution algebra, and taking the $\ell _\nu ^1$-norm. When $\tilde{K}^{-1}$ is multiplied by a polynomial term that does *not* contain a factor $Kh_z$, then a straightforward calculation shows that $||\tilde{K}^{-1}||_{B(\ell _\nu ^1,\ell _\nu ^1)}=(M+1)^{-1}$. The net result is that the impact on the norm of the polynomial term is a scaling by $(M+1)^{-1}$. Therefore, adapting the algorithm from the $Z_{2,1}$ bound calculation, it suffices to apply the following operations to $d_{\delta _2}D{\mathcal {F}}_s(\hat{u}_s + \delta _1 +t\delta _2)h$.

1. Multiply the part of $d_{\delta _2}D{\mathcal {F}}_s(\hat{u}_s +\delta _1 + t\delta _2)h$ in $\ell _\nu ^1({\mathbb {C}}^{n+m})$ by $\frac{1}{M+1}$;
2. Replace all instances of $\zeta (\hat{\psi }_s +\psi (\delta _1))$ with the interval $[-1,1]$;
3. Replace all (remaining) instances of *K* with $(M+1)C_\nu e_0\cdot [-1,1]$;
4. Replace the component of *h* in ${\mathbb {R}}^{n+m+4}$ with the unit interval vector^6^ in ${\mathbb {R}}^{n+m+4}$;
5. Replace all other variables ($\hat{z}_s$, $\hat{\rho }_s$, $\delta _1$, $\delta _2$, the part of *h* in $\ell _\nu ^1({\mathbb {C}}^{n+m})$, and their relevant projections^7^) with bounds for their norms, multiplied by the zero-supported basis element of the relevant space, multiplied by the interval $[-1,1]$;
6. Compute operator norms of the blocks of $\textbf{A}_s$;
7. Complete block multiplication of $\textbf{A}_s$ on the left, take the $\Omega$-norm of the result, followed by an interval supremum (note: $t\in [0,1]$ is also replaced by an interval).

The result is a (crude) upper bound of $\sup _{t\in [0,1]}||A_sd_{\delta _2}D{\mathcal {F}}_s(\hat{u}_s +\delta _1+t\delta _2)h||_\Omega$ for $||h||_\Omega \le 1$. This bound is expected to be *O*(*r*) for *r* small, since the Gateaux derivative $d_{\delta _2}D{\mathcal {F}}_s(\hat{u}_s+\delta _1+t\delta _2)h$ is $O(||\delta _2||)$ for $||\delta _2||\le r$ small.

##### Remark 14

In step 3, the variable replacement negates (by multilinearity of the convolution) the multiplication of the relevant polynomial term by $(M+1)^{-1}$ that was done in step 1, while propagating forward the correct bound $C_\nu$ that results from the interaction between $\tilde{K}^{-1}$ and the $Kz_h*\prod _j(\cdots )$ polynomial term. This somewhat roundabout way of introducing the bound $C_\nu$ in the correct locations is done in order to make the process implementable in generality.

## Specification to Ordinary Differential Equations

In Sect. 3, we demonstrated how rigorous two-parameter continuation of periodic orbits in delay differential equations can be accomplished in such a way that the continuation can pass through degenerate Hopf bifurcations. As ordinary differential equations are a special case—that is, where any delays are identically zero—the theory of the previous sections naturally applies equally to them. However, the formulation of the map (17) can be greatly simplified. Indeed, the reader familiar with numerical methods for periodic orbits has likely noticed that we have not performed the “usual” scaling out by the frequency, so that the period can be abstractly considered as $2\pi$. With delay differential equations, this is not beneficial because it merely moves the frequency dependence into the delayed variables. Additionally, it is the presence of the delays that requires a more subtle analysis of the $Z_2$ bound; see Sect. 4.5.

To compare, with ordinary differential equations *without delays*, the technicalities with the $Z_2$ bound are absent. At the level of implementation, computing second and even third derivatives of the vector field in the Fourier representation is also much easier. In this section, we will present an alternative version of the zero-finding problem of Sect. 3.2 and analogous map *G* from (17). However, we will not discuss the general implementation of the technical bounds *Y* and *Z*, since they are both simpler than the ones we have previously presented for the delay differential equations case, and can be obtained by fairly minor modifications of the bounds from [33]. We have implemented them in general in the codes at [5].

### Desingularization, Polynomial Embedding and Phase Isolation

The set-up now starts with an ordinary differential equation

37 $$\begin{aligned} \dot{y}(t)=f(y(t),\alpha ,\beta ) \end{aligned}$$

again depending on two real parameters $\alpha$ and $\beta$. Performing the same blowup procecure as we did for the delay equations, we define

$$\begin{aligned} \tilde{f}(z,x,a,\alpha ,\beta )=\left\{ \begin{array}{ll} a^{-1}(f(x+a z,\alpha ,\beta )-f(x,\alpha ,\beta )),&{}a\ne 0\\ d_{y}f(x,\alpha ,\beta )z,&{}a=0. \end{array}\right. \end{aligned}$$

The goal is therefore to find a pair (*x*, *z*) such that

$$\begin{aligned} f(x,\dots ,x,\alpha ,\beta )&=0\\ \dot{z}(t)&= \tilde{f}(z(t),x,a,\alpha ,\beta ),\\ ||z||&=1 \end{aligned}$$

where *z* is $\omega$-periodic for an unknown period $\omega$; equivalently, the frequency of *z* is $\psi = \frac{2\pi }{\omega }$. Let $\mu =\psi ^{-1}$ denote the reciprocal frequency. Define $\tilde{z}(t) = z(t\mu )$. Then $\tilde{z}(t)$ is $2\pi$-periodic. Substituting this into the differential equation above and dropping the tildes, we obtain the modified vector field

$$\begin{aligned} \dot{z}(t)&= \mu \tilde{f}(z(t),x,a,\alpha ,\beta ), \end{aligned}$$

where now the scope is that *z* should be $2\pi$-periodic.

If a polynomial embedding is necessary to eliminate non-polynomial nonlinearities, we allow the inclusion of *m* additional scalar equations that must be simultaneously solved, where we introduce an appropriate number *m* of unfolding parameters, $\eta \in {\mathbb {R}}^m$, to balance them. We again use the symbol $\theta _{BC}$ to function that defines these boundary conditions, with the equation being $\theta _{BC}(z(0),x,a,\alpha ,\beta ,\eta )=0$. We use the same anchor condition to handle the lack of isolation from phase shifts.

### Zero-Finding Problem

Abusing notation and assuming now that $\tilde{f}$ is polynomial after the embedding has been taken into account, the zero-finding problem is

38 $$\begin{aligned} {\left\{ \begin{array}{ll} \dot{z} = \mu \tilde{f}(z(t),x,a,\alpha ,\beta ,\eta ),&{} \quad \text {(differential equations)}\\ \Vert z\Vert = 1, &{}\quad \text {(amplitude condition of scaled orbit)}\\ \int \langle z(s),\hat{z}'(s)\rangle ds=0, &{} \quad \text {(anchor condition)}\\ f(x,\alpha ,\beta )=0, &{}\quad (x\ \text {is a steady state})\\ \theta _{BC}(z(0),x,a,\alpha ,\beta ,\eta )=0.&{} \quad \text {(embedding boundary condition)} \end{array}\right. } \end{aligned}$$

where $\hat{z}$ is understood to be a candidate numerical solution. Expanding *z* as a Fourier series with period $2\pi$, we have $z(t) = \sum _{k\in {\mathbb {Z}}}z_ke^{ikt}$ for some complex vectors $z_k$ obeying the symmetry $z_k = \overline{z_{-k}}$. If we now allow $\textbf{f}(z,x,a,\alpha ,\beta ,\eta )$ to be the representation of $\tilde{f}$ as a convolution polynomial in the coefficients $z=(z_k)_{k\in {\mathbb {Z}}}$, then an analogous derivativation to the delay differential equations case produces the map

$$\begin{aligned} G(z,x,a,\psi ,\eta ,(\alpha ,\beta ))&=\left( \begin{array}{c} -iKz + \mu \textbf{f}(z,x,a,\alpha ,\beta ,\eta ) \\ f(x,\alpha ,\beta ) \\ \langle z,K^2\hat{z}\rangle - 1 \\ \langle z,iK\hat{z}\rangle \\ \theta _{BC}(\sum _k z_k,x,a,\alpha ,\beta ,\eta ) \end{array}\right) \end{aligned}$$

defined on the same Banach spaces, with the same codomain. However, this time, one can show that if *f* is $C^k$, then *G* is also $C^{k}$.

## Proving Bifurcations and Bubbles

Modulo non-resonance conditions, we would generically expect Hopf bifurcations to occur on the level curve at amplitude zero of the computed 2-manifolds of Sect. 3. Hopf bubbles are equivalent to curves in the manifold that intersect the amplitude zero surface at two distinct points. As for bubble bifurcations, we can describe these in terms of a the existence of a relative local minimum of a projection of the computed 2-manifold, represented as the graph over amplitude and a distinguished parameter. We develop these points in this section, demonstrating how post-processing of data from the computer-assisted proofs can be used to prove the existence of Hopf bifurcations, bubbles, and degenerate bifurcations.

We should emphasize that to prove the existence of a *single* Hopf bubble, it suffices to identify a curve connecting two points at amplitude zero in the projection of the proven manifold in $\alpha \times \beta \times \text{ amplitude }$ space, for one of $\alpha$ or $\beta$ being fixed. While this does require verifying two Hopf bifurcations, the rest of the proof of an isolated bubble is fairly trivial, requiring only determining a sequence of simplices that enclose the curve.

To simplify the presentation, we will assume throughout this section that the norm on $\Omega$ is such that $||(0,(0,a,0,0,0),0)||=|a|$. That is, the amplitude component is unit weighted relative to the absolute value.

### Hopf Bifurcation Curves

In delay differential equations, the sufficient conditions for the existence of a Hopf bifurcation include the non-resonance check, which involves counting all eigenvalues all eigenvalues of the lienarization on the imaginary axis. This is somewhat beyond the scope of our work here, although there is literature on how this can be done using computer-assisted proofs [4, 19]. In this paper, we will consider the following related notion.

#### Definition 1

The delay differential Eq. (3) has a *Hopf bifurcation curve* $\Theta _H\in \Delta$ if there exists a $C^1$ parametrization $\Delta \ni s\mapsto (\alpha (s),\beta (s),x(s),y(s))$ such that *x*(*s*) is a steady state solution and *y*(*s*) is a periodic orbit at parameters $(\alpha (s),\beta (s))$, such that:

- $\Theta _H(0)$ and $\Theta _H(1)$ are on the boundary of $\Delta$, and $\Theta _H(t)$ is in the interior of $\Delta$ for $t\in (0,1)$.
- $x\circ \Theta _H=y\circ \Theta _H$; in other words, *y* is a steady state on restriction to the image of $\Theta _H$.
- For $s\in \Delta \setminus \{\Theta _H(t):t\in [0,1]\}$, *y*(*s*) is not a steady state.

Contrary to the usual Hopf bifurcation, we do not reference the *direction* of the bifurcation, even along one-dimensional curves in the manifold of periodic orbits.

**Fig. 5:**
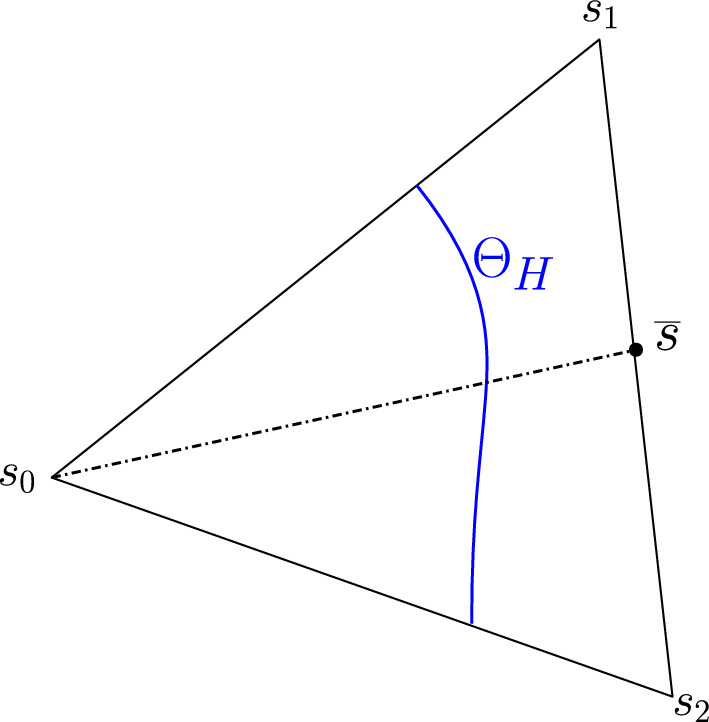
Briefly, the partial derivatives of the amplitude of the family of periodic orbits, with respect to the simplex parametrization variables, have the same sign. From the assumptions of the proposition, the sign of the amplitude along the edge connecting $s_1$ to $s_2$ is opposite to that of the amplitude at $s_0$. Forming a ray (dashed-dotted line) connecting $s_0$ to some point $\overline{s}$ on the edge connecting $s_1$ to $s_2$, the mean-value theorem guarantees the existence of a unique point along the ray at which the amplitude is zero. Parameterizing over all $\overline{s}$ on the edge $s_1$ to $s_2$, the Hopf curve $\Theta _H$ (blue) is constructed (Color figure online)

The existence of a Hopf bifurcation curve can be proven using post-processing of a validated contiuation on given simplex on which one *expects* a Hopf bifurcation to occur. The idea is that on a simplex that encloses a Hopf bifurcation, the amplitude in the blown-up variables (see Sect. 3.1) is expected to cross through zero. Since we are working in two-parameter continuation, such a crossing point should persist as a curve. A geometric construction based on the partial derivatives of the amplitude with respect to the simplex paramaterization can be used to construct this curve. See Fig. 5 for a visualization.

To establish a more constructive proof, we need to introduce a few projection maps. Let $u=(z^M,\rho )\in \Omega ^M$, $\rho =(x,a,\psi ,\eta ,\alpha ,\beta )$. Denote $\pi ^a u = a$ the projection to the amplitude component, and $\pi ^{(\alpha ,\beta )}u =(\alpha ,\beta )$ the projection onto the parameters. Similarly, write $\pi ^a\rho = a$. Denote the three vertices of the standard simplex $\Delta$ as $s_0 = (0,0)$, $s_1 = (1,0)$ and $s_2 = (0,1)$.

#### Proposition 12

Suppose a simplex containing the interpolated numerical data $\hat{u}_s$ has been validated, for $s\in \Delta .$ Let $r>0$ be an associated validation radius from the radii polynomial. Let $c=Z_0+Z_1 + Z_2(r)$. Suppose the following are satisfied.

- For $$k=1,2$$,
  $$\begin{aligned} {[}\pi ^a(\hat{u}_{s_0})-r,\pi ^a(\hat{u}_{s_0}) +r] \cdot [\pi ^a(\hat{u}_{s_k})-r,\pi ^a(\hat{u}_{s_k}) +r]<0, \end{aligned}$$
  where the multiplication is in the sense of interval arithmetic.
- The sign of the interval $[\pi ^a(\hat{u}_s) - r, \pi ^a (\hat{u}_s)+r]$ is constant for *s* on the edge of $\Delta$ not incident with $s_0$; that is, on the edge connecting $s_1$ with $s_2$.
- Let $\partial _s$ denote the Fréchet derivative operator with respect to the variable $s\in \Delta$. For $h\in {\mathbb {R}}^2$, define
  39 $$\begin{aligned} \tilde{\partial }_s a(s)h&\overset{\textrm{def}}{=}\pi ^a\left( -S_s\partial _s[\textbf{J}_s] (\hat{u}_s + \delta _r)h\right) ,\qquad \tilde{\partial }_s u(s) \overset{\textrm{def}}{=}-A_s\partial _s [{\mathcal {F}}_s](\hat{u}_s + \delta _r), \nonumber \\ \gamma&= \frac{c}{1-c}\sup _{s\in \Delta }||\tilde{\partial }_s u(s)||, \end{aligned}$$
  where $\delta _r=\overline{B_r(0)}\subset \Omega ^M$. That is, $\gamma$ is a real interval and $\tilde{\partial }_s a(s)$ is an interval in $({\mathbb {R}}^2)^*$. Let $\hat{\gamma } = \sup \gamma$. The components of $\tilde{\partial }_s a(s) + \hat{\gamma }[-1,1]^2$ have the same sign for $s\in \Delta$.

Then there is a *unique* (up to parameterization) Hopf bifurcation curve $\Theta _H:[0,1]\rightarrow \Delta$, and each of $\Theta _H(0)$ and $\Theta _H(1)$ lie on the edges of $\Delta$ that are incident with $s_0$.

#### Proof

Suppose the radii polynomial proves the existence of $u_s\in B_r(\hat{u}_s)$ such that ${\mathcal {F}}_s(u_s)=0$. The $C^1$ parametrization of the steady state (*x*(*s*)), periodic orbit (*y*(*s*)) and amplitude parametrization is a consequence of the computer-assisted proof. $\partial _s u_s$ exists, and $\partial _s {\mathcal {F}}_s(u_s) + D{\mathcal {F}}_s(u_s)\partial _s u_s = 0$. We claim $D{\mathcal {F}}_s(y)$ is boundedly invertible for $y\in \overline{B_r(\hat{u}_s)}$. First,

$$\begin{aligned} I-A_sD{\mathcal {F}}_s(y) = (I-A_sA_s^\dagger ) + A_s(A_s^\dagger -D{\mathcal {F}}_s(\hat{u}_s)) + A_s(D{\mathcal {F}}_s(\hat{u}_s) - D{\mathcal {F}}_s(y)) \end{aligned}$$

whose norm is bounded above by $Z_0 + Z_1 + Z_2(r)=c$. Since the radii polynomial has validated $\hat{u}_s$, it follows that $c<1$. By the Neumann series, $A_sD{\mathcal {F}}_s(y)$ is boundedly invertible, with norm bounded by $(1-c)^{-1}$. Since $A_s$ is boundedly invertible by its construction, the same is true of $D{\mathcal {F}}_s(y)$.

It follows that $\partial _s u_s =-D{\mathcal {F}}_s(u_s)^{-1} \partial _s{\mathcal {F}}_s(u_s)$. Observe that

$$\begin{aligned} (A_s-D{\mathcal {F}}_s(u_s)^{-1})\partial _s{\mathcal {F}}_s(\hat{u}_s + \delta _r) =(AD{\mathcal {F}}_s(u_s))^{-1}(AD{\mathcal {F}}_s(u_s)-I)A_s\partial _s{\mathcal {F}}_s(\hat{u}_s + \delta _r) \end{aligned}$$

By previous estimates, the above is bounded in norm by $\gamma$. Since $\partial _s[{\mathcal {F}}_s](u_s)\in \partial _s[{\mathcal {F}}_s](\hat{u}_s +\delta _r)$—see Sect. 4.2—we have

40 $$\begin{aligned} \partial _s u_s \in -A_s\partial _s {\mathcal {F}}(\hat{u}_s + \delta _r) + \overline{B_\gamma (0)}. \end{aligned}$$

Note that $\tilde{\partial }_s a(t)h$ is precisely the amplitude component of $\partial _s (u_s h)|_{s=t}$.

Consider the line $[0,1]\mapsto t\mapsto s_1 + t(\overline{s} -s_1)$, where $\overline{s}$ is any point on the edge connecting $s_1$ to $s_2$ in $\Delta$. Consider the function $g(t) = \pi ^a u_{s_1 + t(\overline{s} - s_1)}$. By the assumptions of the proposition and the inclusion (40), we have $g(0)g(1)<0$ and $\frac{d}{dt}g(t)\ne 0$ for $t\in (0,1)$. By the mean-value theorem, there is a unique $t^*\in (0,1)$ such that $g(t^*)=0$. Moreover, $t^*=t^*(\overline{s})$ depends continuously on $\overline{s}$. Letting $p:[0,1]\rightarrow \Delta$ be a $C^1$ parametrization of the edge connecting $s_1$ to $s_2$, it follows by the definition of the map ${\mathcal {F}}_s$ that $\Theta _H=t^*\circ p$ is a Hopf bifurcation curve. $\square$

We emphasize that most of the sufficient conditions of Proposition 12 can be checked using the output of the validation of a simplex. We also mention specifically that in the second point, the sign of $[\pi ^a(\hat{u}_s) - r, \pi ^a (\hat{u}_s)+r]$ need only be verified to match at $s=s_1$ and $s=s_2$, due to linearity of $\hat{u}_s$. The exception is the computation of $\tilde{\partial }_s a$ and $\gamma$. The former is a finite computation, and the latter can be bounded in a straightforward manner; see Sect. 4.2 for details concerning the structure of $\partial _s{\mathcal {F}}_s$.

#### Remark 15

Proposition 12 is stated in such a way that we assume $s_0$ and the edge connecting $s_1$ to $s_2$ lie on the opposite sides of Hopf curve $\Theta _H$. This is done *almost* without loss of generality. First, if the Hopf curve should bisect the simplex in such a way that $s_1$ or $s_2$ is separated from its opposing edge, a suitable re-labeling of the nodes transforms the problem into the form of the proposition. Second, a problem can arise if the Hopf curve intersects one of the vertices $s_j$, $j=0,1,2$. Similarly, numerical difficulties can arise if the intersections of $\Theta _H$ with the edges of $\Delta$ occur very close to the vertices. These problems can be alleviated by a careful selection of numerical data.

#### Remark 16

A Hopf bifurcation curve necessarily generates a continuum of Hopf bifurcations. Along any curve in $\Delta$, one will find a Hopf bifurcation at each transversal intersection with $\Theta _H$.

### Degenerate Hopf Bifurcations

In this section, we consider how one might prove the existence of a degenerate Hopf bifurcation, be it a bubble bifurcation or otherwise. For a first definition, we consider the bubble bifurcation.

#### Definition 2

A *bubble bifurcation (with quadratic fold)* occurs at $(\alpha ^*,\beta ^*)$ in (13) if there exists a Hopf curve $\Theta _H$ with $(\pi ^\alpha u_{\Theta _H(t^*)},\pi ^\beta u_{\Theta _H(t^*)})=(\alpha ^*,\beta ^*)$ for some $t^*\in (0,1)$, such that

- The projection of $\Theta _H$ into the $(\alpha ,\beta )$ plane can be realized as a $C^2$ graph $\beta = \beta (\alpha )$.
- $$\beta (\alpha ^*)=\beta ^*$$, $$\beta '(\alpha ^*)=0$$, and $$\beta ''(\alpha ^*)\ne 0$$.
- There is a $C^2$ diffeomorphism $h:D\rightarrow h(D)$ defined on a neighbourhood *D* of $\Theta _H(t^*)\in \Delta$, such that $h(\Theta _H(t^*))=(\alpha ^*,0)$, and the periodic orbit *y* (see Definition 1) is a steady state if and only if $\pi _2 h(s)=0$, where $\pi _2$ is the projection onto the second factor. Also, the projection $\pi _1$ onto the first factor satisfies $\pi _1 h(s) = \pi ^a u_{s}$ for all $s\in D$.
- $(\alpha ^*,0)$ is a strict local extremum of the map $(\alpha ,a)\mapsto \pi ^\beta u_{h^{-1}(\alpha ,a)}$.

Our perspective is that a bubble bifurcation is characterized by the existence of a manifold of periodic orbits, parameterized in terms of amplitude and a parameter, such that at an isolated critical point, the projection of the manifold onto the other parameter has a local extremum. The parametrization of $\beta$ near $\alpha ^*$ and the local minimum condition reflects the observation that a fold in the curve of Hopf bifurcations is sufficient condition for the birth of the bubble. That is, we have a family of bubbles (loops of Hopf bifurcations) that can be parameterized by $\beta$ in an interval of the form $(\beta ^*-\delta ,\beta ^*]$ or $[\beta ^*,\beta ^*+\delta )$, for $\delta >0$ small. This is a consequence of the $(\alpha ,a)$-parametrization of the simplex.

#### Remark 17

We include the adjective “quadratic” to describe the fold in the Hopf curve to contrast with a related definition in Sect. 6.2.3, where the condition $\beta ''(\alpha ^*)\ne 0$ will be weakened.

#### Preparations

It will facilitate the presentation of the bubble bifurcation if we are able to construct the first and second derivatives of $s\mapsto u_s$, the zeroes of (20), with respect to the simplex parameter *s*. This was partially done in the proof of Proposition 12, but we will elaborate further here.

To compute the derivatives, the idea is to formally differentiate $s\mapsto {\mathcal {F}}_s(u_s)$ with respect to *s* twice, allowing for the introduction of $\partial _s u_s$ and $\partial _s^2 u_s$. The result is a system of three nonlinear equations, and solving for $(u_s,\partial _s u_s,\partial _s^2 u_s)$ amounts to computing zeroes of a map

41 $$\begin{aligned} \Omega ^3\ni (u_s,\partial _s u_s) \mapsto ({\mathcal {F}}_s(u_s), {\mathcal {F}}_s^{[1]}(u_s,\partial _s u_s),{\mathcal {F}}_s^{[2]} (u_s,\partial _s u_s,\partial _s^2 u_s)) \end{aligned}$$

##### Remark 18

It may be unclear whether the second derivative of $s\mapsto {\mathcal {F}}_s(u_s)$ can be given meaning. Indeed, as we have previously explained in Remark 11, the second Fréchet derivative of $u\mapsto {\mathcal {F}}_s(u)$ does not, in general, exist. The (formal) second derivative of both sides of ${\mathcal {F}}_s(u_s)=0$ produces

$$\begin{aligned} D{\mathcal {F}}_s(u_s)\partial _s^2 u_s + \partial _s^2{\mathcal {F}}_s(u_s) + 2\partial _s [D{\mathcal {F}}_s](u_s)\partial _s u_s + D^2 {\mathcal {F}}_s(u_s)[\partial _s u_s,\partial _s u_s]=0, \end{aligned}$$

and we can see that it is in fact possible to interpret $D^2{\mathcal {F}}_s(u_s)[\partial _s u_s,\partial _s u_s]$ as the Gateaux derivative of $w\mapsto D{\mathcal {F}}_s(w)\partial _s u_s$, at $w=u_s$ and in the direction $\partial _s u_s$. However, even this may not exist as an element of $K_\nu ({\mathbb {C}}^{n+m})$, since the Fourier components of $u_s$ are generally not band-limited. It is therefore necessary to specify the codomain of (41) a bit more carefully. We will not elaborate on this subtlety here; the ramifications of this will be the topic of some of our future work. However, in the case of *ordinary differential equations*, there is no major complication. When there are no delays (or when they are all zero), ${\mathcal {F}}_s$ is twice continuously differentiable provided the same is true of *f*.

Regardless how the partial derivatives are enclosed, the following equivalent version of Proposition 12 is available.

##### Proposition 13

Assume the existence of a family of zeroes of (41) parameterized by $s\in \Delta$ and close to a numerical interpolant $\hat{\textbf{u}}_s=(\hat{u}_s,\partial _s \hat{u}_s,\partial _s^2\hat{u}_s)$ has been proven, in the sense that we have identified $r>0$ such that there is a unique zero of (41), for each $s\in \Delta$, in the ball $B_r(\hat{\textbf{u}}_s)$. Assume the topology on this ball is such that the components $\hat{a}_s=\pi ^a \hat{u}_s$ satisfy

$$\begin{aligned} ||(\hat{a}_s^{[1]} - a_s^{[1]})h||\le r_a^{(1)}|h|, \end{aligned}$$

for $h\in {\mathbb {R}}^2$, and $||\hat{a}_s^{[0]} - a_s^{[0]}||\le r_a^{(0)}$. Suppose

- For $j=1,2$, $[\hat{a}^{[0]}_{s_0}-r_a^{(0)}, \hat{a}^{[0]}_{s_0}+r_a^{(0)}]\cdot [\hat{a}^{[0]}_{s_j} -r_a^{(0)},\hat{a}^{[0]}_{s_j}+r_a^{(0)}]<0$, where the multiplication is in the sense of interval arithmetic.
- The sign of $[\hat{a}^{[0]}_{s}-r_a^{(0)},\hat{a}^{[0]}_{s} +r_a^{(0)}]$ is constant for *s* on the edge of $\Delta$ not incident with $s_0$; that is, on the edge connecting $s_1$ to $s_2$.
- The components of the interval vector $\hat{a}_s^{[1]} +r_a^{(1)}[-1,1]^2$ in ${\mathbb {R}}^{2*}$ has the same sign for $s\in \Delta$.

Then there exists a Hopf bifurcation curve $\Theta _H:[0,1]\rightarrow \Delta$, each of $\Theta _H(0)$ and $\Theta _H(1)$ lie on the edges of $\Delta$ that are incident with $s_0$, and $\Theta _H$ is $C^2$. $\square$

Comments analogous to those appearing in Remark 15 apply here as well. Note that Proposition 13 requires only the first derivatives, which can be enclosed using (40). However, if the existence of the second derivatives are in question, then $\Theta _H$ can at best be proven to be $C^1$.

##### Definition 3

A triple of line segments $(\overline{v}_1, \overline{v}_*, \overline{v}_2)$ in $\Delta$ is $s_0$-*oriented* if there exist points $t_1,t_*,t_2$ on the edge $(s_1,s_2)$, such that

- $\overline{v}_1$ is a subset of the line connecting $s_0$ to $t_1$;
- $\overline{v}_*$ is a subset of the line connecting $s_0$ to $t_*$;
- $\overline{v}_2$ is a subset of the line connecting $s_0$ to $t_2$;
- Under the total order on the edge $(s_1,s_2)$ defined by $a\le b \Leftrightarrow ||s_1-a||_2\le ||s_1-b||_2$, we have $\overline{v}_1\le \overline{v}_*\le \overline{v}_2$.

In this case the associated *wedge cover* is the simplex in $\Delta$ with vertices $s_0,t_1$ and $t_2$, and it is denoted $W(\overline{v}_1,\overline{v}_*,\overline{v}_2)$.

These $s_0$-oriented line segments will be used to enclose potential bubble bifurcation points. Some finer control can be specified with the following.

##### Definition 4

Let $(\overline{v}_1,\overline{v}_*,\overline{v}_2)$ be an $s_0$-oriented triple in $\Delta$. Let $\Theta _H$ be a Hopf curve in $\Delta$ associated to a family $u_s$ of zeroes of ${\mathcal {F}}_s$. A *Hopf-bounding trapezoid in* $W(\overline{v}_1, \overline{v}_*,\overline{v}_2)$ is a trapezoid contained in $W(\overline{v}_1,\overline{v}_*,\overline{v}_2)$ whose edges include both $\overline{v}_1$ and $\overline{v}_2$, and such that the other edges, denoted $\overline{w}_+$ and $\overline{w}_-$, satisfy $\pi ^a u_s <0$ for $s\in \overline{w}_-$, and $\pi ^a u_s>0$ for $s\in \overline{w}_+$.

It is straightforward to verify that, under the assumptions of Proposition 13, a trapezoid in $W(\overline{v}_1,\overline{v}_*,\overline{v}_2)$ is Hopf-bounding provided two of its edges match $\overline{v}_1$ and $\overline{v}_2$, while

$$\begin{aligned} \hat{a}_s^{[0]}+r_a^{(0)}&<0,\quad s\in \overline{w}_-\\ \hat{a}_s^{[0]}-r_a^{(0)}&>0,\quad s\in \overline{w}_+. \end{aligned}$$

#### Enclosure of a Bubble Bifurcation

The following proposition provides verifiable conditions for the existence of a bubble bifurcation. Some are more explicit than others.

##### Proposition 14

Let the hypotheses of Proposition 13 be satisfied. Let $(\overline{v}_1,\overline{v}_*,\overline{v}_2)$ be an $s_0$-oriented triple of line segments in $\Delta$. Suppose the topology on $B_r(\hat{\textbf{u}}_s)$ is such that the components $\pi ^\alpha \hat{u}_s^{[k]} = \hat{\alpha }_s^{[k]}$, $\pi ^\beta \hat{u}_s^{[k]} = \hat{\beta }_s^{[k]}$, $\pi ^a\hat{u}_s^{[k]} = \hat{a}_s^{[k]}$ satisfy

$$\begin{aligned} ||(\hat{\alpha }^{[k]}_s - \alpha ^{[k]}_s)[h_1,\dots ,h_k]||&\le r_\alpha ^{(k)}|h_1|\cdots |h_k|\\ ||(\hat{\beta }^{[k]}_s - \beta ^{[k]}_s)[h_1,\dots ,h_k]||&\le r_\beta ^{(k)}|h_1|\cdots |h_k|\\ ||(\hat{a}^{[k]}_s - a^{[k]}_s)[h_1,\dots ,h_k]||&\le r_a^{(k)}|h_1|\cdots |h_k| \end{aligned}$$

for *k*-tuples $h_1,\dots ,h_k\in {\mathbb {R}}^2$, and $k=0,1,2$. With the empty tuple ($k=0$), the norm reduces to absolute value on ${\mathbb {R}}$. Let $\textbf{r}_\kappa$, for $\kappa \in \{\alpha ,\beta ,a\}$ be interval vectors in ${\mathbb {R}}^2$ such that $(\textbf{r}_\kappa )_j =[-1,1]r_\kappa ^{(1)}$, for $j=1,2$. Finally, given $s\in \Delta$, denote by $V_s$ the set

$$\begin{aligned} V_s = \{v\in {\mathbb {R}}^2: (\hat{a}_s^{[1]}+\textbf{r}_a)\cdot v=0, \ \ ||v||_2=1\}. \end{aligned}$$

Suppose the following are satisfied.

1. $(\hat{\alpha }_s^{[1]} + \textbf{r}_\alpha )\cdot v_s$ is bounded away from zero, for all $s\in \Delta$ and all $v_s\in V_s$.
2. $$\inf \hat{a}_{\overline{v}_i}^{[0]}+r_a^{(0)}<0<\sup \hat{a}_{\overline{v}_i}^{[0]}-r_a^{(0)}$$ for $$i=1,2$$.
3. $$\inf \hat{a}_{\overline{v}_*}^{[0]}+r_a^{(0)}<0<\sup \hat{a}_{\overline{v}_*}^{[0]}-r_a^{(0)}$$.
4. $$\hat{\beta }^{[0]}_{\overline{v}_*}+r_\beta ^{(0)} < \hat{\beta }^{[0]}_{\overline{v}_i}-r_\beta ^{(0)}$$ for $$i=1,2$$.
5. The components of $\hat{a}_s^{[1]}+\textbf{r}_a$ are bounded away from zero for all $s\in \Delta$.
6. The determinant of the $2\times 2$ interval matrix
  42 $$\begin{aligned} \left( \begin{array}{cc} (\hat{\alpha }^{[1]}_s)_1 + [-1,1]r_\alpha ^{(1)} &{} (\hat{\alpha }^{[1]}_s)_2 + [-1,1]r_\alpha ^{(1)} \\ (\hat{a}^{[1]}_s)_1 + [-1,1]r_a^{(1)} &{} (\hat{a}^{[1]}_s)_2 + [-1,1]r_a^{(1)} \end{array}\right) \end{aligned}$$
  is bounded away from zero for $s\in \Delta$.
7. Defining
  43 $$\begin{aligned} c_s = \frac{1}{||\hat{a}^{[1]}_s + \textbf{r}_a||^2} (\hat{\beta }^{[1]}_s+\textbf{r}_\beta )\cdot (\hat{a}^{[1]}_s +\textbf{r}_a), \end{aligned}$$
  it holds that the interval $\hat{\beta }^{[2]}_s[v_s,v_s] - c_s\hat{a}^{[2]}_s[v_s,v_s] + (r_\beta ^{(2)} + ||c_s||_2r_a^{(2)})[-1,1]$ is bounded away from zero for all $s\in \Delta$ and $v_s\in V_s$.
8. The matrix
  44 $$\begin{aligned} \left( \begin{array}{cc} \beta _s^{[2]}[\partial _\alpha s,\partial _\alpha s] + \beta _s^{[1]}\partial _\alpha ^2 s &{} \beta _s^{[2]}[\partial _a s, \partial _\alpha s] + \beta _s^{[1]}\partial _a\partial _\alpha s \\ \beta _s^{[2]}[\partial _a s,\partial _\alpha s] + \beta _s^{[1]} \partial _a\partial _\alpha s &{} \beta _s^{[2]}[\partial _a s, \partial _a s] + \beta _s^{[1]}\partial _a^2 s \end{array}\right) \end{aligned}$$
  is uniformly (for $s\in \Delta$) definite, where defining $h:\Delta \rightarrow {\mathbb {R}}^2$ by $h(s)=(\alpha _s,a_s)$, the partial derivatives in the matrix above are
  $$\begin{aligned} \left[ \begin{array}{cc} \partial _\alpha s&\partial _a s \end{array}\right]&=Dh(s)^{-1}\\ \partial _\alpha ^2 s&=-Dh(s)^{-1}D^2 h(s)[\partial _\alpha s, \partial _\alpha s]\\ \partial _a\partial _\alpha s&=-Dh(s)^{-1}D^2 h(s)[\partial _a s,\partial _\alpha s]\\ \partial _a^2 s&=-Dh(s)^{-1}D^2 h(s)[\partial _a s,\partial _a s]. \end{aligned}$$

Then, there exists a bubble bifurcation with quadratic fold at some $(\alpha ^*,\beta ^*)$ in the projection of $\Theta _H\cap W(\overline{v}_1,\overline{v}_*,\overline{v}_2)$ onto the $(\alpha ,\beta )$ plane.

##### Proof

By a suitable reparametrization, we may assume that $||\Theta _H'(t)||=1$ for all $t\in (0,1)$. Denote $a(s)=\pi ^a u_s$. The definition of the Hopf curve is that $a(\Theta _H(t))=0$ or all $t\in [0,1]$. As consequence, $(\partial _s a)\Theta _H'=0$, so that $\Theta _H'$ is dual to the orthogonal complement of $\partial _s a$. That is, $\Theta _H'\in \cup _s V_s$. Now,

$$\begin{aligned} \frac{d}{dt}\alpha ^{[0]}_{\Theta _H(t)} = \alpha _{\Theta _H(t)}^{[1]} \Theta _H'\in \bigcup _{s\in \Delta }\left( \bigcup _{v_s\in V_s} (\hat{\alpha }_s^{[1]}+\textbf{r}_\alpha )\cdot v_s \right) , \end{aligned}$$

which is bounded away from zero by assumption (condition 1 of the proposition). It follows that $t\mapsto \alpha _{\Theta _H(t)}^{[0]}$ is monotone, so $\Theta _H$ can be parameterized by $\alpha$, for $\alpha$ in the monotone range of $t\mapsto \alpha _{\Theta _H(t)}^{[0]}$. Consequently, the projection of the Hopf curve $\Theta _H$ in the $(\alpha ,\beta )$ plane can be represented as a graph $\beta =\beta (\alpha )$.

Conditions 2–4 of the proposition guarantee that each of the segments $\overline{s}_1$, $\overline{s}_2$ and $\overline{t}$ enjoy the following properties:

- As one-dimensional manifolds, they enclose an intersection with the Hopf curve;
- The $\beta$-components of $\overline{v}_1$ and $\overline{v}_2$ are strictly greater than those of $\overline{v}_*$.

As consequence, $\beta =\beta (\alpha )$ possesses an internal (to its domain, relative to the previously-computed range) critical point which is a global minimizer. Let this point be $\alpha ^*$, so $\beta (\alpha ^*)\overset{\textrm{def}}{=}\beta ^*$. Let the associated point on the simplex be $\Theta _H(t^*)$.

We wish to show that $\beta ^*$ is a strict, isolated minimum of $\beta$. We will do that by proving $\beta ''(\alpha ^*)\ne 0$. It is enough to prove that $\frac{d^2}{dt^2}\beta ^{[0]}_{\Theta _H(t)}\ne 0$ whenever $\frac{d}{dt}\beta ^{[0]}_{\Theta _H(t)}=0$. If the latter is satisfied, then we have simultaneously

$$\begin{aligned} a_{\Theta _H(t)}^{[1]}\Theta _H'&=0\\ \beta _{\Theta _H(t)}^{[1]}\Theta _H'&=0. \end{aligned}$$

Since $\Theta _H'\ne 0$, it must be the case that $\alpha ^{[1]}_{\Theta _H(t)}$ and $\beta _{\Theta _H(t)}^{[1]}$ are colinear. By assumption 5, $\alpha ^{[1]}_{\Theta _H(t)}$ is bounded away from zero, so the quantity $c_s$ of (43) is well-defined and

45 $$\begin{aligned} \beta _{\Theta _H(t)}^{[1]}\in c_{\Theta _H(t)} a_{\Theta _H(t)}^{[1]}.\end{aligned}$$

On its own, (45) might not seem particular useful. However, consider that

$$\begin{aligned} \frac{d^2}{dt^2}\beta ^{[0]}_{\Theta _H(t)}&=\beta _{\Theta _H(t)}^{[2]}[\Theta _H',\Theta _H'] + \beta _{\Theta _H(t)}^{[1]}\Theta _H''\\ 0&=a_{\Theta _H(t)}^{[2]}[\Theta _H',\Theta _H'] + a_{\Theta _H(t)}^{[1]}\Theta _H'', \end{aligned}$$

where the second equation comes from taking a second derivative of the definition $a(\Theta _H(t))=0$ of the Hopf curve. Using the second equation together with (45), we can get the inclusion

$$\begin{aligned} \beta _{\Theta _H(t)}^{[1]}\Theta _H'' \in -c_{\Theta _H(t)} a_{\Theta _H(t)}^{[2]}[\Theta _H',\Theta _H'], \end{aligned}$$

thereby removing the dependence on the second derivative $\Theta _H''$ of the Hopf curve. Substituting into the expression for the second derivative of $t\mapsto \beta _{\Theta _H(t)}$, we get

$$\begin{aligned} \frac{d^2}{dt^2}\beta ^{[0]}_{\Theta _H(t)}&\in \beta _{\Theta _H(t)}^{[2]}[\Theta _H',\Theta _H'] -c_{\Theta _H(t)}a_{\Theta _H(t)}^{[2]}[\Theta _H',\Theta _H']\\&\subset \bigcup _{s\in \Delta }\left( \bigcup _{v_s\in V_s} \hat{\beta }^{[2]}_s[v_s,v_s] - c_s\hat{a}^{[2]}_s[v_s,v_s] + (r_\beta ^{(2)} + ||c_s||_2r_a^{(2)})[-1,1]\right) , \end{aligned}$$

which is bounded away from zero by condition 7. Therefore, $\beta ''(\alpha ^*)\ne 0$.

Next, we need to verify the local parametrization of the simplex near $\Theta _H(t^*)$ in terms of $(\alpha ,a)$. This is a fairly direct consequence of the inverse function theorem, using the condition 6 of the proposition. This shows that the function *h* defined in condition 8 of the proposition defines a local diffeomorphism near $\Theta _H(t^*)$. The Hessian of $\Gamma :(\alpha ,a)\mapsto \beta _{h^{-1}(\alpha ,a)}$ can be computed by implicit differentiation; if $h(s)=(\alpha ,a)$, then the Hessian is precisely the $2\times 2$ matrix of condition 8. Since this matrix is uniformly definite on the simplex $\Delta$, every critical point must be a local extremum. We already know that $\partial _\alpha \Gamma (\alpha ^*,0)=\beta '(\alpha ^*)=0$. The other partial derivative $\partial _a\Gamma (\alpha ^*,0)$ is zero due to the amplitude symmetry of periodic orbits. Therefore, $(\alpha ^*,0)$ is a strict local extremum of the map $(\alpha ,a)\mapsto \pi ^\beta u_{h^{-1}(\alpha ,a)}$. $\square$

##### Remark 19

Checking conditions 2–6 of the proposition is fairly routine. Conditions 1 and 7, however, deserve some extra attention. If the step size is small, we should expect the *derivatives* of the solution $s\mapsto u_s$ to be close to constant. In this way, the set $V_s$ should not vary too much (in a Hausdorff sense). $V_s$ geometrically consists of two arcs on the unit circle, and we expect the angles defining these arcs to be nearly constant over the simplex. Consequently, the interval computations of conditions 1 and 7 are, indeed, implementable, but the *feasibility* of the checks — that these intervals are bounded away from zero — will be strongly influenced by the size of the radius, *r*, and any weighting in the norm. As for condition 8, we have not included all of the associated radii, but since all of the intermediary derivatives appearing in the matrix (44) admit (by assumption) rigorous enclosures, the matrix is implementable. Therefore, condition 8 can be checked using a suitable package to compute eigenvalues of interval matrices.

##### Remark 20

Condition 4 is formulated in such a way that $\beta ^*$ is a local *minimum* of the curve $\beta =\beta (\alpha )$. This condition can be reformulated in a straightforward way to allow it instead to be a local maximum. Also, comments analogous to those appearing in Remark 15 apply here as well.

##### Corollary 15

It suffices to verify the conditions 6,7,8 of Proposition 14 for all *s* in a given Hopf-bounding trapezoid in $W(\overline{v}_1,\overline{v}_*,\overline{v}_2)$.

#### A Degenerate Hopf Bifurcation Without Second Derivatives

In Remark 18, we pointed out that, unfortunately, the lack of second-differentiability of the map ${\mathcal {F}}_s$ is a serious obstruction to computing second derivatives $\partial _s^2 u_s$ and, consequently, checking all the conditions of Proposition 14. While it is not a problem when all of the delays are zero (i.e. an ODE), we would like to provide a constructive result for delay equations. Along these lines, let us slightly weaken Definition 2.

##### Definition 5

A *degenerate Hopf bifurcation* occurs at $(\alpha ^*,\beta ^*)$ in (13) if there exists a Hopf curve $\Theta _H$ with $(\pi ^\alpha u_{\Theta _H(t^*)},\pi ^\beta u_{\Theta _H(t^*)})=(\alpha ^*,\beta ^*)$ for some $t^*\in (0,1)$, such that

- The projection of $\Theta _H$ into the $(\alpha ,\beta )$ plane can be realized as a $C^1$ graph $\beta = \beta (\alpha )$;
- $$\beta (\alpha ^*)=\beta ^*$$ and $$\beta '(\alpha ^*)=0$$;
- There is a $C^1$ diffeomorphism $h:D\rightarrow h(D)$ defined on a neighbourhood *D* of $\Theta _H(t^*)\in \Delta$, such that $h(\Theta _H(t^*))=(\alpha ^*,0)$, and the periodic orbit *z* (see Definition 1) is a steady state if and only if $\pi _2 h(s)=0$, where $\pi _2$ is the projection onto the second factor. Also, the projection $\pi _1$ onto the first factor satisfies and $\pi _1 h(s) = \pi ^a u_{s}$ for all $s\in D$.

The main difference between the above and Definition 2 is we no longer require that $\beta ^*$ is an extremum of $\beta$. We also do not make any specifications concerning the geometry of the implicit map $(\alpha ,a)\mapsto \beta$ near the Hopf bifurcation curve. Clearly, a bubble bifurcation with quadratic fold satisfies the conditions of the above definition. However, the new definition permits other types of degenerate Hopf bifurcations, including Bautin bifurcations. The following proposition can now be proven using the same ideas as Proposition 14.

##### Proposition 16

Assume a family of zeroes $u_s$ of the map ${\mathcal {F}}_s$ has been proven, in addition to the first derivatives $\partial _s u_s$, close to a numerical interpolant $\hat{\textbf{u}}_s=(\hat{u}_s,\partial _s \hat{u}_s)$, in the sense that we have identified $r>0$ such that there is a unique zero of (41), for each $s\in \Delta$, in the ball $B_r(\hat{\textbf{u}}_s)$. Suppose the topology on $B_r(\hat{\textbf{u}}_s)$ is such that the components $\pi ^\alpha \hat{u}_s^{[k]} = \hat{\alpha }_s^{[k]}$, $\pi ^\beta \hat{u}_s^{[k]} = \hat{\beta }_s^{[k]}$, $\pi ^a\hat{u}_s^{[k]} = \hat{a}_s^{[k]}$ satisfy

$$\begin{aligned} ||(\hat{\alpha }^{[k]}_s - \alpha ^{[k]}_s)[h_1,\dots ,h_k]||&\le r_\alpha ^{(k)}|h_1|\cdots |h_k|\\ ||(\hat{\beta }^{[k]}_s - \beta ^{[k]}_s)[h_1,\dots ,h_k]||&\le r_\beta ^{(k)}|h_1|\cdots |h_k|\\ ||(\hat{a}^{[k]}_s - a^{[k]}_s)[h_1,\dots ,h_k]||&\le r_a^{(k)}|h_1|\cdots |h_k| \end{aligned}$$

for *k*-tuples $h_1,\dots ,h_k\in {\mathbb {R}}^2$, and $k=0,1$. With the empty tuple ($k=0$), the norm reduces to absolute value on ${\mathbb {R}}$. Let $(\overline{v}_1,\overline{v}_*,\overline{v}_2)$ be an $s_0$-oriented triple of line segments in $\Delta$. Suppose conditions 1–3, 5 and 6 of Proposition 14 are satisfied. Then, there exists a degenerate Hopf bifurcation at some $(\alpha ^*,\beta ^*)$ in the projection of $\Theta _H\cap W(\overline{v}_1,\overline{v}_*,\overline{v}_2)$ onto the $(\alpha ,\beta )$ plane.

##### Corollary 17

It suffices to verify condition 6 of Proposition 16 for all *s* in a Hopf-bounding trapezoid in $W(\overline{v}_1,\overline{v}_*,\overline{v}_2)$.

## Examples

### Extended Lorenz-84 System

The extended Lorenz-84 system is the following system of four coupled ODEs:

$$\begin{aligned} \dot{u}_1&=-u_2^2 - u_3^2 - au_1 - af - bu_4^2\\ \dot{u}_2&= u_1u_2 - cu_1u_3 - u_2 + d\\ \dot{u}_3&= cu_1u_2 + u_1u_3 - u_3\\ \dot{u}_4&= -eu_4 + bu_4u_1 + \mu \end{aligned}$$

We consider the parameters $a = 0.25$, $b=0.987$, $d=0.25$, $e=1.04$, $f=2$ to be fixed, while $\mu$ and *c* are treated as parameters.

**Fig. 6:**
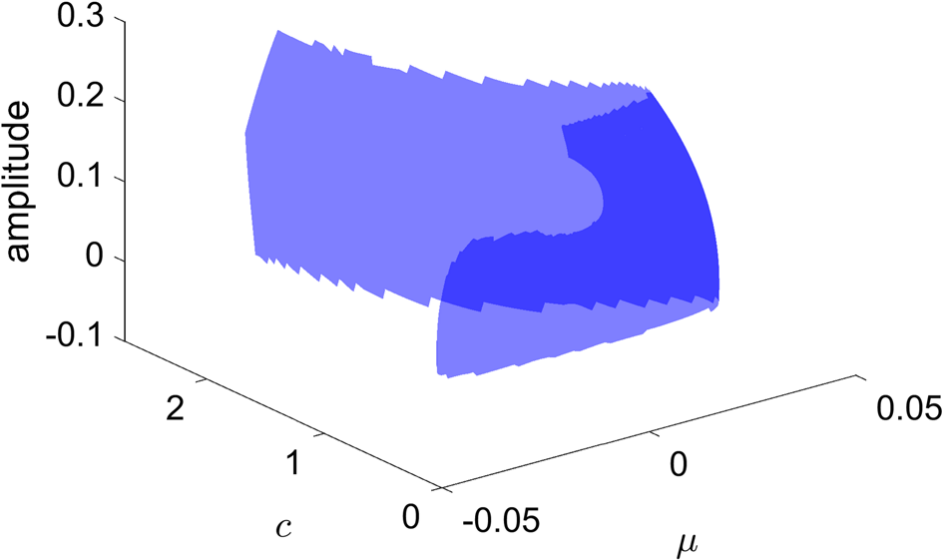
The manifold of (proven) periodic orbits in the extended Lorenz-84 system, in the projection of amplitude and the parameters $c,\mu$. Note the square-root curvature of amplitude near $\mu \approx 0.04$ (right side of plot), indicative of Hopf bubbles. There are over 10,000 simplices, so to provide a clean figure, we have not plotted the edges

**Fig. 7:**
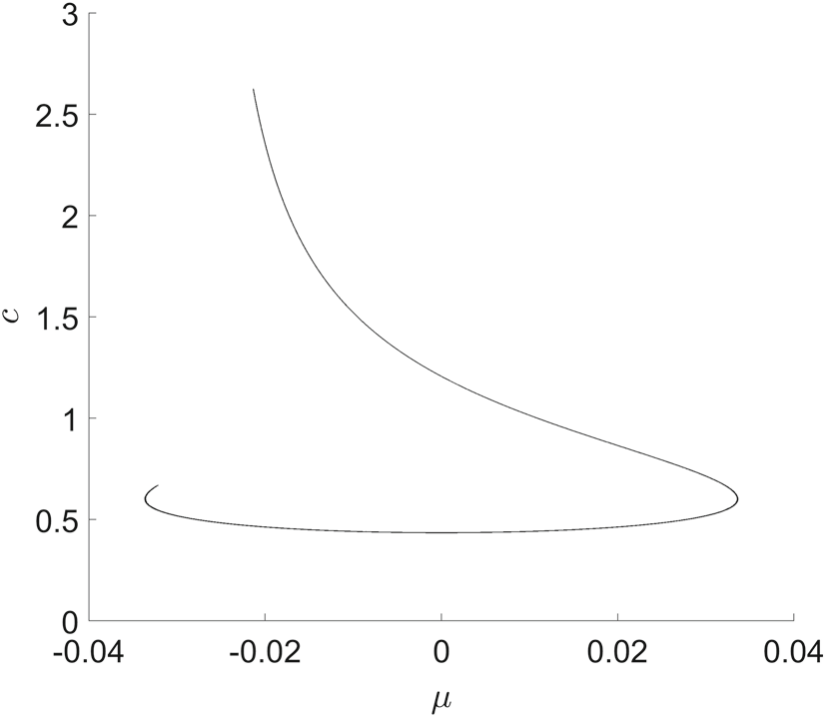
Intersection of the surface in Fig. 6 with the amplitude zero plane, which corresponds to the Hopf bifurcation curve. Hopf bubbles are present in the former figure near $\mu \approx 0.04$, so the fold in the present figure likely corresponds to a bubble bifurcation with quadratic fold. There is a symmetric fold near $\mu \approx -0.04$, and with respect to the other variable near $\mu \approx 0$, for a total of three bubble bifurcations

We started the continuation at $c=1$, close to a Hopf bifurcation. Using a step size of 0.02, we generated an approximate triangulation of the manifold with 11,928 simplices (including simplices created by adaptive refinement needed for proofs). We used $N=5$ Fourier modes. To capture a “wider” section of the manifold, we restricted the simplex growth to amplitude in the interval $[-0.1,0.3]$. Figure 6 is a plot of the triangulation, projected into amplitude and parameter space, while we restricted to the zero amplitude plane in Fig. 7 to generate a plot of the Hopf bifurcation curve. The former figure allows for visualization of the traditional square-root amplitude curvature near the Hopf bifurcation curve. Interesting, far from the bifurcation curve, there appears to be a near-circular “hole” in the manifold. We have not studied its structure in detail, and have no insight into its significance. The restriction of the amplitude to $[-0.1,0.3]$ results in the top and bottom edges appearing “ragged”, since simplices can not organically grow to produce hexagon patches. The latter Fig. 7 indicates the presence of three bubble bifurcations.

### Time-Delay SI Model

We consider the time-delay SI model

$$\begin{aligned} \dot{y}(t)=-y(t) + R_0e^{-py(t-\tau )}y(t)(1-y(t)), \end{aligned}$$

for which there is an analytical proof of a bubble bifurcation [18] near $(R_0,p)\approx (2.1474, 1.6617)$, for delay $\tau =10$. We will replicate this analysis using our rigorous two-parameter continuation.

#### Set-up

We begin by desingularizing the vector field. Writing $y = x + a\tilde{y}$, for *x* being a steady state, we get, dropping the tilde,

$$\begin{aligned} \dot{y}= -y + g(a,y_\tau p)R_0e^{-px}x(1-x) + R_0e^{-px} e^{-apy_\tau }(-a y^2 + y(1-2x)), \end{aligned}$$

where $y_\tau = y(t-\tau )$, and *g* is defined by

$$\begin{aligned} g(a,y)&=\left\{ \begin{array}{ll} a^{-1}(e^{-ay }-1),&{} a\ne 0 \\ -y,&{}a=0. \end{array}\right. \end{aligned}$$

Observe, $$\partial _y g(a,y)=-e^{-ay}$$ and

$$\begin{aligned} g(a,y)=-y\sum _{k=1}^\infty \frac{1}{k!}(-ay)^{k-1}. \end{aligned}$$

*g* is indeed analytic. Whenever *g* (or its derivatives) must be rigorously evaluated, we construct Taylor polynomials of sufficiently high order and propagate error from the remainder accordingly.

Now we polynomialize. Define $z_2=e^{-a py}$ and $z_1=g(a,yp)$. Then

$$\begin{aligned} \dot{z}_1&= -pz_2\big (-y + R_0e^{-px}z_1(t-\tau )x(1-x) + R_0e^{-px}z_2(t-\tau )(-a y^2 + y(1-2x)) \big )\\ \dot{z}_2&= -a p z_2\big (-y + R_0e^{-px}z_1(t-\tau ) x(1-x) + R_0e^{-px}z_2(t-\tau )(-a y^2 + y(1-2x)) \big ) \end{aligned}$$

They can be more compactly written as $\dot{z}_1 = -z_2\dot{z}_0$ and $\dot{z}_2=-apz_2\dot{z}_0$. We also have the implied boundary conditions

$$\begin{aligned} z_1(0)&=g(a,z_0(0)p)\\ z_2(0)&=e^{-ap z_0(0)}, \end{aligned}$$

where $z_0=y$. We need two unfolding parameters to compensate for the two extra boundary conditions.

##### Lemma 18

If *z* is a periodic solution of

$$\begin{aligned} \dot{z}_0(t)&=-z_0(t) + R_0\mu z_1(t-\tau )x(1-x) + R_0\mu z_2(t-\tau )(-az_0(t)^2 + z_0(t)(1-2x))\\ \dot{z}_1(t)&=-pz_2(t)\dot{z}_0(t) + \eta _1\\ \dot{z}_2(t)&=-apz_2(t)\dot{z}_0(t) + \eta _2 \end{aligned}$$

for some $\eta _1,\eta _2\in {\mathbb {R}}$, and *z* satisfies $z_1(0)=g(a,z_0(0)p)$ and $z_2(0)=e^{-apz_0(0)}$, then $\eta _1=\eta _2=0$.

##### Proof

First, suppose $\eta _2\ne 0$. Then necessarily, $z_2$ has constant sign because the differential equation for $z_2$ is affine-linear and $\eta _2\ne 0$. Since $z_2(0)>0$, we must have $z_2>0$. But this means that

$$\begin{aligned} \frac{\dot{z}_2(t)}{z_2(t)} + ap\dot{z}_0(t) = \frac{\eta _2}{z_2(t)}, \end{aligned}$$

a contradiction, since the left side is periodic and $\eta _2\ne 0$. We may therefore assume that $\eta _2=0$. Then $\dot{z}_2(t) = apz_2(t)\dot{z}_0(t)$, and it follows again that $z_2>0$. But this means

$$\begin{aligned} \frac{\dot{z}_1(t)}{z_2(t)} + p\dot{z}_0(t) =\frac{\eta _1}{z_2(t)}, \end{aligned}$$

and since the left-hand side is periodic, it follows that $\eta _1=0$. $\square$

To complete the polynomial embedding, we further polynomialize the parameters. This is done to ensure compatibility with the numerical implementation. We define $\mu = R_0e^{-px}$, so that the complete polynomialized vector field is

$$\begin{aligned} \dot{z}_0(t)&=-z_0(t) + \mu z_1(t-\tau )x(1-x) + \mu z_2(t-\tau )(-az_0(t)^2 + z_0(t)(1-2x))\\ \dot{z}_1(t)&=-pz_2(t)\dot{z}_0(t) + \eta _1\\ \dot{z}_2(t)&=-apz_2(t)\dot{z}_0(t) + \eta _2. \end{aligned}$$

The complete set of boundary conditions is

$$\begin{aligned} 0&=z_1(0) - g(a,z_0(0)p)\\ 0&=z_2(0) - e^{-apz_0(0)}\\ 0&=\mu - R_0e^{-px} \end{aligned}$$

In the terminology of Remark 5, the embedding dimension is $m=3$. The steady-state equation is scalar, and is given by

$$\begin{aligned} 0=-x + \mu x(1-x). \end{aligned}$$

#### Results

**Fig. 8:**
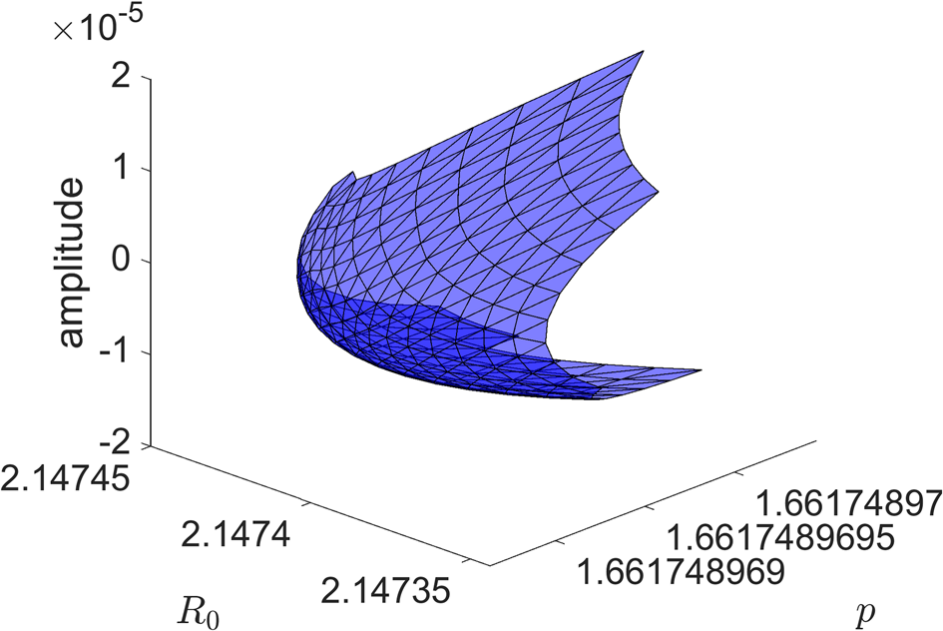
The manifold of (proven) periodic orbits in the time delay SI model. A very small step size was necessary to get proofs without too many adaptive refinement steps. The $Y_0$ bound is the clear bottleneck

**Fig. 9:**
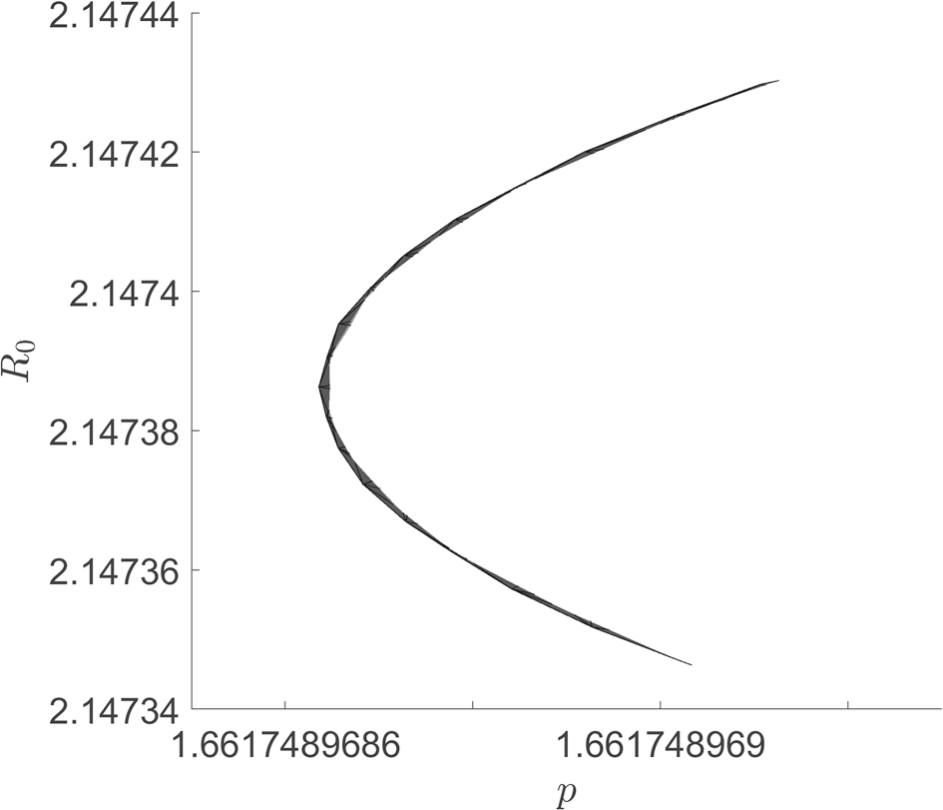
Intersection of the surface from Fig. 8 with the amplitude zero, which corresponds to a Hopf bifurcation curve in the time-delay SI model. The validation radius is $2\times 10^{-5}$ over the entire manifold, so the location $R_0\approx 2.1474$ of the bubble bifurcation is consistent with the analysis of Leblanc. A tighter validation radius could be obtained with a smaller step size. Because of how the curve is plotted, skew simplices from them 2-manifold have the effect of making the curve appear “thicker” in some parts than others

We validated a patch of manifold initially with 406 simplices at a step size of $5\times 10^{-6}$. In the validation of nearly every simplex, three layers of adaptive refinement were needed to keep the $Y_0$ bound under control. We have plotted the manifold in Fig. 8 without the refinements included. The projection into the $(R_0,p)$ plane is provided in Fig. 9. The results are consistent with the analysis of Leblanc [18].

### FitzHugh–Nagumo Equation

**Fig. 10:**
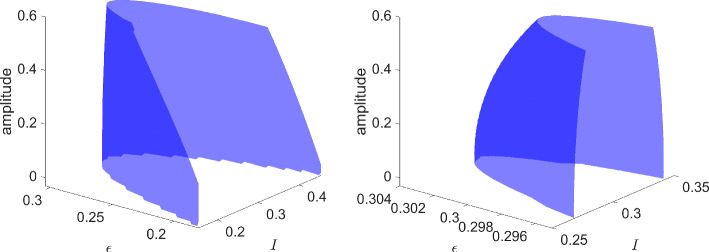
Left: Projection of the manifold of periodic orbits for the FitzHugh–Nagumo equation into $\epsilon \times I\times \text{ amplitude }$ space. At this level of scaling, the curvature of the manifold is not easily visible. Right: zoomed-in portion near the bubble bifurcation. Here, the curvature is more easily seen. There are over 9000 simplices in the left figure, so we have not plotted the edges

**Fig. 11:**
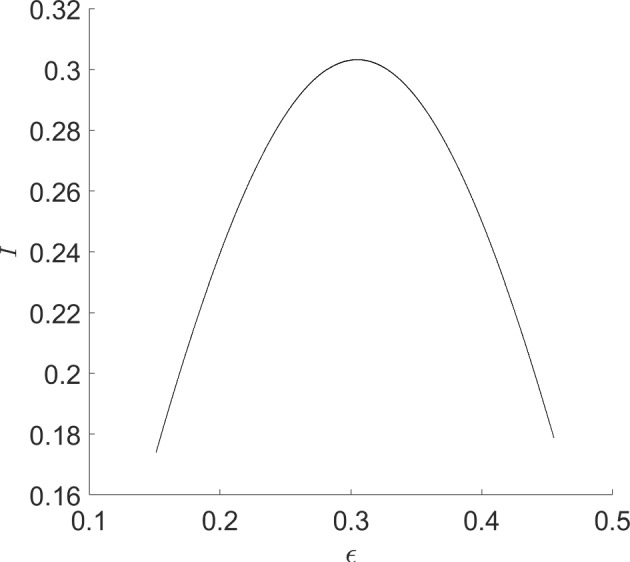
Intersection of the surface from the left pane of Fig. 10 with the amplitude zero plane. This corresponds to the Hopf bifurcation curve for the FitzHugh–Nagumo system. The curve resembles a parabola, with a bubble bifurcation at its vertex

The FitzHugh–Nagumo ODE is

$$\begin{aligned} \dot{u}&=u(u-\alpha )(1-u)-w+I\\ \dot{w}&=\epsilon (u-\gamma w) \end{aligned}$$

for scalar parameters $\alpha ,\epsilon ,\gamma ,I$. It is a cubic vector field in the state variables, and numerical simulations suggest the existence of Hopf bubbles (see Section 5.8 of [7]). We fix $\alpha = 0.1,\, \gamma = 1$, while leaving $\epsilon$ and *I* as parameters for the continuation. We start the continuation near $(\epsilon ,I)=(0.3,0.3)$ and compute a triangulation of the manifold with 9006 simplices (including those needed for adaptive refinement) at step size 0.01. For this example, we used $N=7$ Fourier modes. A plot of the proven simplices from the manifold is provided in Fig. 10. The Hopf bifurcation curve appears in Fig. 11.

### An ODE with a Periodic Orbit 2-Manifold Resembling a Fish

Consider the three-dimensional ODE system

46 $$\begin{aligned} \dot{y}_1&= \beta y_1 - y_2 - y_1 (y_1^2 + y_2^2 + y_3^2 + \alpha ^2) \end{aligned}$$

47 $$\begin{aligned} \dot{y}_2&= y_1 + \beta y_2 - y_2(y_1^2 + y_2^2 + \epsilon y_3^2 + \alpha ^2) \end{aligned}$$

48 $$\begin{aligned} \dot{y}_3&= -y_3^5 + 3y_3^3 - 0.01y_3 + 0.1\alpha + 0.01(y_1^2+y_2^2), \end{aligned}$$

for parameters $\alpha ,\beta$, and a real control parameter $\epsilon$. When $\epsilon =1$, a change of variables to cylindrical coordinates shows that periodic orbits are in one-to-one correspondence with solutions (*r*, *z*) of the set of algebraic equations

$$\begin{aligned} 0&=\beta - r^2 - \alpha ^2 - z^2\\ 0&=-z^5 + 3z^3 - 0.01z + 0.1\alpha + 0.01r^2. \end{aligned}$$

**Fig. 12:**
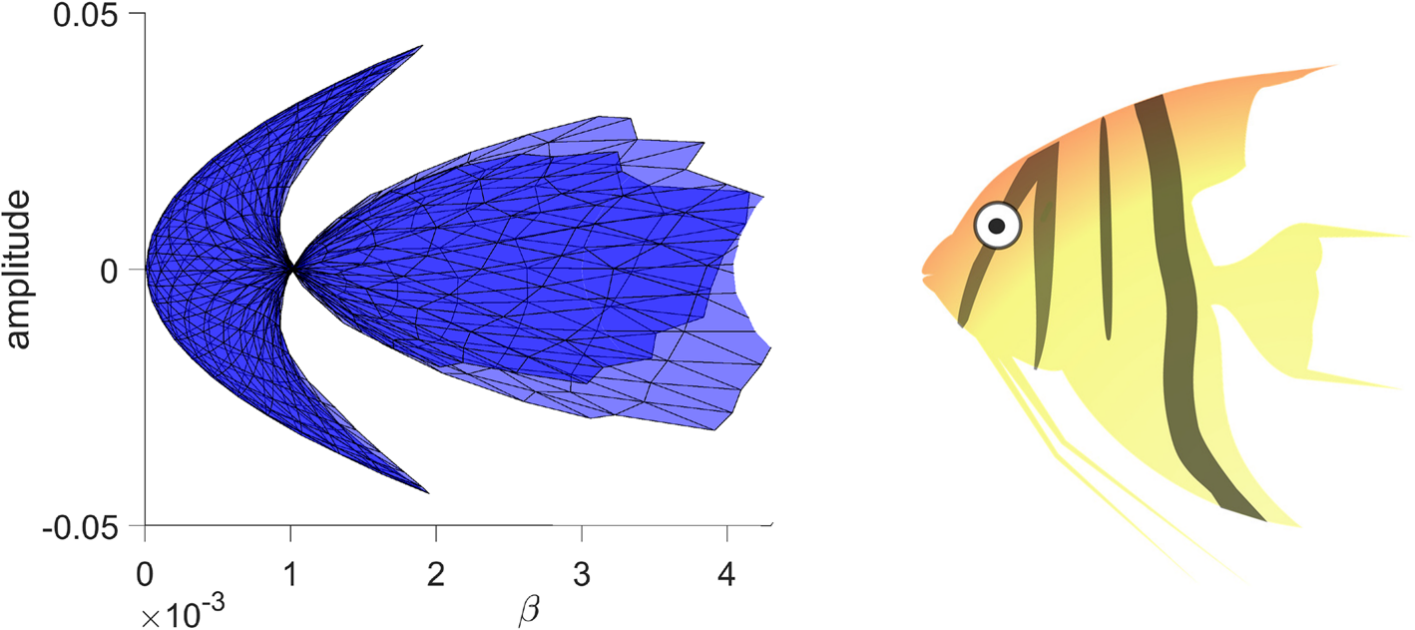
Left: Projection of the manifold of periodic orbits for the ODEs (46)–(48) into $\text{ amplitude } \times \alpha \times \beta$ space, viewed from the $\alpha$ axis. Note the “pinching” of the manifold, at which this projection becomes singular, near $\beta \approx 10^{-3}$. Right: an illustration of an angelfish, for comparison

**Fig. 13:**
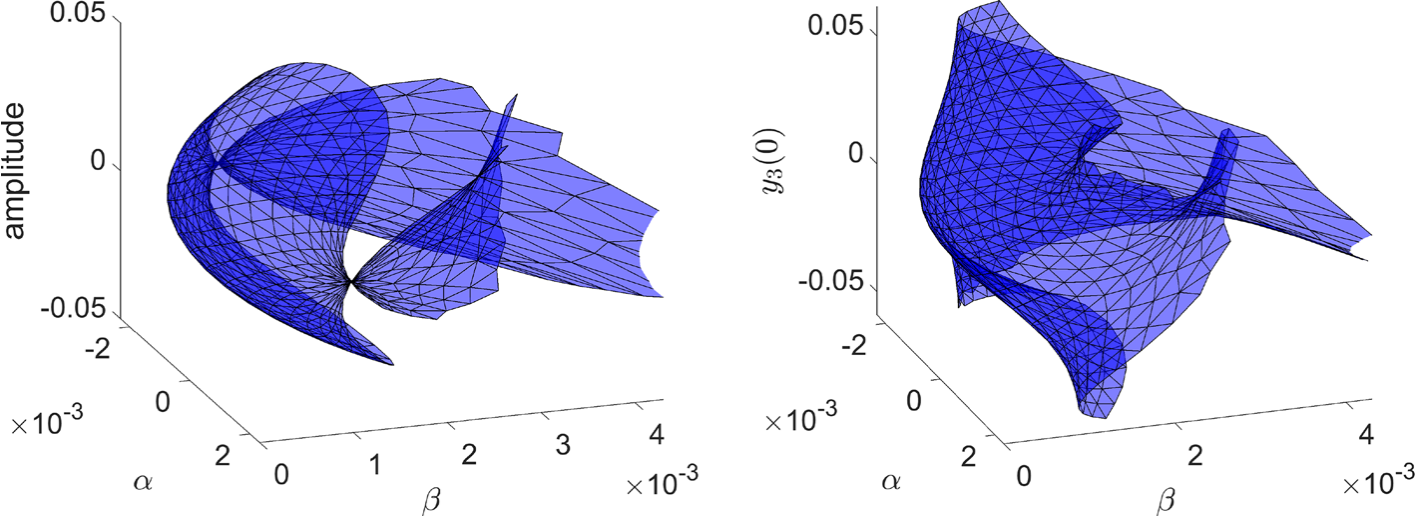
Left: Projection of the manifold of periodic orbits for the ODEs (46)–(48) into $\text{ amplitude } \times \alpha \times \beta$ space. There are Hopf bubbles prior to the pinching phenomenon that happens near $\beta \approx 10^{-3}$. Right: projection into $\alpha \times \beta \times y_3(0)$ space, where $y_3$ denotes the third component of the periodic orbit in the blown-up coordinates. The pinching point in the left figure projection is caused by a pair of simultaneous folds, clearly visible in the right figure projection

**Fig. 14:**
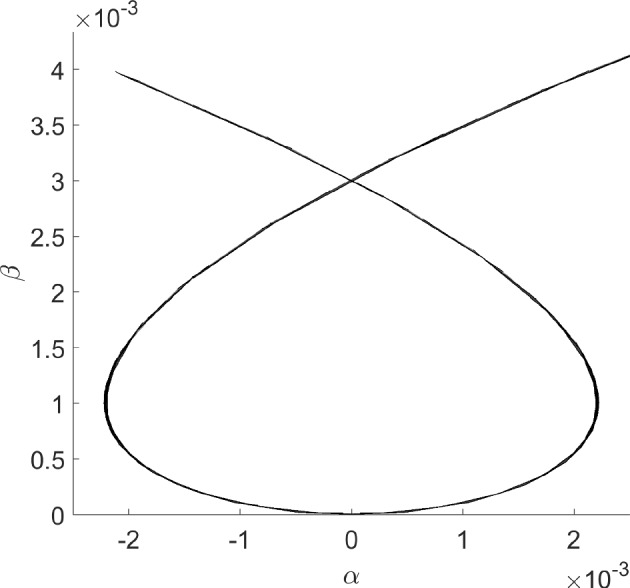
Intersection of the surface from the left pane of Fig. 13 with the amplitude zero, which corresponds to a Hopf bifurcation curve for the ODE system (46)–(48). There is a bubble bifurcation at $(\alpha ,\beta )=(0,0)$. The apparent self-intersection of the Hopf curve is a consequence of the projection, and does not represent a bifurcation point. Because of how the curve is plotted, skew simplices from them 2-manifold have the effect of making the curve appear “thicker” in some parts than others

When $\epsilon \ne 1$, the radial symmetry in $(y_1,y_2)$ is broken and this change of variables is no longer informative. We set $\epsilon =0.8$ in (46)–(48) and used our validated continuation scheme to rigorously compute a 2-manifold of periodic orbits. In the projection of amplitude and parameters $(\alpha ,\beta )$, the result is a figure that qualitatively resembles an angelfish. See Fig. 12. In this projection, the manifold has several folds and appears to exhibit a singularity where it pinches onto a single point. Plotting the manifold in a different projection more clearly allows us to see that this singularity is merely an artifact of the projection; see Fig. 13. The Hopf bifurcation curve is plotted in Fig. 14. For this example, we used $N=9$ Fourier modes and a step size 0.01. We computed a comparatively small portion of the manifold, since the interesting geometry was localized close to $(\alpha ,\beta )=(0,0)$. We computed and validated 1007 simplices. This example did not require any adaptive refinement.

## Discussion

We have proposed validated continuation as an alternative way of exploring degenerate Hopf bifurcations. In combination with rigorous numerics and additional *a posteriori* post-processing, one can prove the existence of Hopf bifurcation curves, bubble bifurcations and some other degenerate Hopf bifurcation. The library *BiValVe* is rather flexible, and with the additions of the present paper, can handle multiparameter continuation problems for periodic orbits in ordinary and delay differential equations, near and far from Hopf bifurcations.

Without access to second derivatives of solutions of the zero-finding problem (20), it is difficult to prove bubble bifurcations with quadratic folds. That is, we are only able to prove the weaker characterization of Definition 5. This is a major barrier in applying the method to delay equations. We believe that a suitable re-formulation of the zero-finding problem, taking into account the additional unbounded operators that result from derivatives with respect to frequency, could resolve the issue.

Another way to prove the “quadratic fold” part of the bubble bifurcation would be to compute the second derivatives of Hopf bifurcation curves without using the machinery of sequences spaces. Along these lines, it would be interesting to use pseudo-arclength continuation to continue Hopf bifurcation curves directly. Computer-assisted proofs of isolated Hopf bifurcations in delay differential equations are completed in [4], and with minimal changes, pseudo-arclength continuation could be used to do continuation of Hopf bifurcations. The map from [4] is finite-dimensional and as smooth as the delay vector field, so the derivatives of the Hopf curve could be rigorously computed that way instead. However, this trick can not be used to prove that $(\alpha ,a)\mapsto \pi ^\beta u_{h^{-1}(\alpha ,a)}$ has a strict local extremum at the bifurcation point. Indeed, the latter map is defined in terms of the periodic orbits themselves, rather than the algebraic properties of the vector field and the eigenvalues of the linearization.

As remarked in [31], the $Z_2$ bound associated to delay periodic orbit validation suffers from a fundamental limitation: it scales linearly with respect to the number of Fourier modes. Therefore, while we have not needed to use many Fourier modes in our examples, it would be very costly (or infeasible) to do continuation of a periodic orbit that required many modes to represent. This is because the $Y_0$ bound is naturally dependent on step size, so even if an isolated solution has an exceptionally good numerical defect, a very small step size might be needed to hedge against a large $Z_2$. In this way, while we can compute manifolds of periodic orbits with delay near (degenerate) Hopf bifurcations, we expect that in large-amplitude regimes or for complicated orbits, completing a validation would be difficult. To compare, the situation is far better for ordinary differential equations. The $Z_2$ bound is generally unharmed by having many Fourier modes, and second derivatives of the solutions can be computed by solving an auxiliary zero-finding problem using similar techniques from rigorous numerics.

There are other codimension-2+ bifurcations that could be studied from the point of view of validated multi-parameter continuation. For example, the cusp bifurcation should be amenable to this type of analysis, and is simpler than the present work because it involves only bifurcations of fixed points rather than periodic orbits. There is also the Bautin bifurcation, for which the analysis of Sect. 6 could be replicated. In fact, our continuation scheme is able to validate manifolds of periodic orbits passing through Bautin points. As a very brief final example, recall the normal form (3)–(4), which has a Bautin bifurcation at $(x,y)=(0,0)$ at the parameters $(\alpha ,\beta )=(0,0)$. Periodic orbits in this ODE are equivalent (by a change of variables to polar coordinates) to scalar solutions *r* of

49 $$\begin{aligned} 0=r(\beta + \alpha r^2 - r^4). \end{aligned}$$

**Fig. 15:**
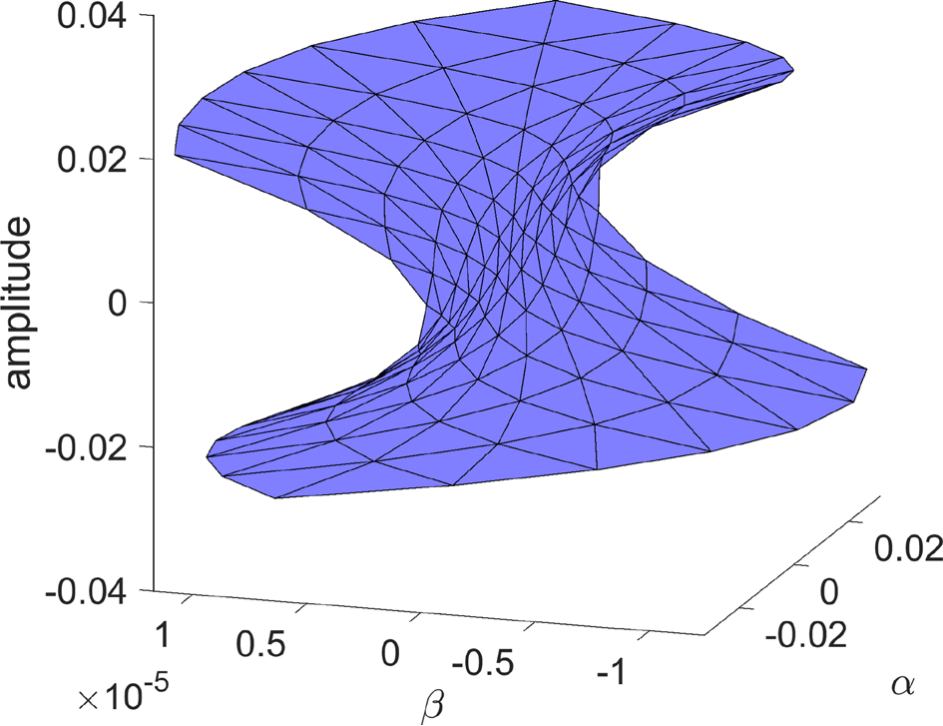
Proven section of the manifold of periodic orbits associated to the Bautin normal form. Note that the amplitude is in fact equal to *r* from equation (49)

Agnostic to this particular representation of the zeroes, our code is able to validate a large section of the manifold of periodic orbits directly from the ODEs. See Fig. 15. As expected, we were able to validate this manifold using very few Fourier modes: three, in this case.

Hopf bubbles have been observed in the Mackey-Glass equation [15] at the classical parameters, and some of our preliminary investigations suggest that the equation possesses a bubble bifurcation. It would be interesting to use a combination of polynomial embedding and blow-up to investigate this bifurcation. However, the added complexity of using both blow-up and polynomial embedding presents a challenge; the resulting (polynomial) delay vector field ends up being high-order with dozens of distinct nonlinear terms.

## Data Availability

The data sets generated during and/or analysed during the current study are available in the BiValVe repository, https://github.com/elenaquei/bubbles/releases/tag/v1.

## Footnotes

1 It becomes notationally cubmersome to include references to weights, or to explicitly label the different norms, so we will refrain from doing so unless absolutely necessary.

2 Including any weighting, which we recall is implicit.

3 The absolute value of $\psi _\textbf{1}$ is precisely the weight attributed to the frequency component, relative to the absolute value norm on ${\mathbb {R}}$.

4 Note that the components of this “unit” interval vector might be uneven due to weighting.

5 Note that if the norm on ${\mathbb {C}}^{n+m}$ associated to $\ell _\nu ^1({\mathbb {C}}^{n+m})$ is itself weighted, this must be taken into account.

6 Note that the components of this “unit” interval vector might be uneven due to weighting.

7 See footnote 5 concerning the projections of *h* in $\ell _\nu ^1({\mathbb {C}}^{n+m})$.
